# Contact‐free radar recordings of body movement can reflect ultradian dynamics of sleep

**DOI:** 10.1111/jsr.13687

**Published:** 2022-07-06

**Authors:** Hanne Siri Amdahl Heglum, Henning Johannes Drews, Håvard Kallestad, Daniel Vethe, Knut Langsrud, Trond Sand, Morten Engstrøm

**Affiliations:** ^1^ Department of Neuromedicine and Movement Science, Faculty of Medicine and Health Sciences Norwegian University of Science and Technology (NTNU) Trondheim Norway; ^2^ Novelda AS Trondheim Norway; ^3^ Department of Mental Health Norwegian University of Science and Technology Trondheim Norway; ^4^ Department of Public Health University of Copenhagen Copenhagen Denmark; ^5^ Division of Mental Health Care St Olavs University Hospital Trondheim Norway; ^6^ Department of Neurology and Clinical Neurophysiology St Olavs University Hospital Trondheim Norway

**Keywords:** actigraphy, LIDS, REM/NREM cycles, sleep monitoring, UWB radar

## Abstract

This work aimed to evaluate if a contact‐free radar sensor can be used to observe ultradian patterns in sleep physiology, by way of a data processing tool known as Locomotor Inactivity During Sleep (LIDS). LIDS was designed as a simple transformation of actigraphy recordings of wrist movement, meant to emphasise and enhance the contrast between movement and non‐movement and to reveal patterns of low residual activity during sleep that correlate with ultradian REM/NREM cycles. We adapted the LIDS transformation for a radar that detects body movements without direct contact with the subject and applied it to a dataset of simultaneous recordings with polysomnography, actigraphy, and radar from healthy young adults (*n* = 12, four nights of polysomnography per participant). Radar and actigraphy‐derived LIDS signals were highly correlated with each other (*r* > 0.84), and the LIDS signals were highly correlated with reduced‐resolution polysomnographic hypnograms (*r*_*radars*_ >0.80, *r*_*actigraph*_ >0.76). Single‐harmonic cosine models were fitted to LIDS signals and hypnograms; significant differences were not found between their amplitude, period, and phase parameters. Mixed model analysis revealed similar slopes of decline per cycle for radar‐LIDS, actigraphy‐LIDS, and hypnograms. Our results indicate that the LIDS technique can be adapted to work with contact‐free radar measurements of body movement; it may also be generalisable to data from other body movement sensors. This novel metric could aid in improving sleep monitoring in clinical and real‐life settings, by providing a simple and transparent way to study ultradian dynamics of sleep using nothing more than easily obtainable movement data.

## 1 INTRODUCTION

The gold standard of sleep monitoring, polysomnography (PSG), clearly displays the cycling of distinct physiological stages during sleep (Berry et al., 2020; Rechtschaffen & Kales, 1968). However, PSG is not always an ideal solution; it depends on multiple on‐body electrodes that can be experienced as uncomfortable, the equipment is expensive, and the interpretation laborious. For practical reasons, it is not considered a good option for long term monitoring or population studies (Kushida et al., 2005). In those situations where PSG is not desirable or feasible, wrist actigraphy has been the most well‐accepted alternative for getting an objective measure of sleep (Sadeh, 2011; Smith et al., 2018). But actigraphy today typically limits itself to identifying circadian patterns of rest and activity. Although earlier works have noted the presence of patterns in actimetric movement data during sleep and explored their relation to other aspects of sleep physiology (Dement & Kleitman, 1957; Muzet, Naitoh, Townsend, & Johnson, 1972; Naitoh, Muzet, Johnson, & Moses, 1973; Schulz & Salzarulo, 2012; Wilde‐Frenz & Schultz, 1983), in modern actigraphy this source of potentially valuable information is largely disregarded. However, in a fairly recent large‐scale analysis of movement during human sleep, Winnebeck et al. introduced a simple and transparent analysis tool designed to exploit more of the full potential of actigraphy (Winnebeck, Fischer, Leise, & Roenneberg, 2018). By converting locomotor inactivity to “Locomotor Inactivity During Sleep” (LIDS), they found that patterns of residual activity were exposed and enhanced in a manner that reflected the underlying ultradian sleep cycles.

In our previous work, we explored how a contact‐free radar sensor able to measure body movement can be used to differentiate between sleep and wake, using methods inspired by actigraphy (Heglum et al., 2021). Contact‐free monitoring has advantages over actigraphy in certain settings, that is, in a psychiatric hospital, where the use of on‐body sensor equipment can be challenging or even dangerous. A permanently mounted radar sensor can record continuously over an indefinite time, with no inconvenience whatsoever to the subject.

In the present work, we sought to investigate if more complex aspects of sleep can be studied with this radar sensor. Specifically, we sought to explore if the LIDS analysis technique can be modified for use with radar‐recorded movement data, to evaluate the similarity of the resulting radar‐derived LIDS signals to actigraphy‐derived LIDS and PSG dynamics, and to compare our work with the previously published results (Winnebeck et al., 2018). Using a data set from young healthy adults sleeping in a hospital environment and recorded simultaneously with PSG, actigraphy, and two radar sensors, we calculated and analysed inactivity profiles for each night with data from each sensor. For ease of comparison, we also introduced a transformation and smoothing of the PSG hypnograms intended to be analogous with LIDS.

The specific objectives of the analyses were: (1) Evaluate the similarity between LIDS signals derived from radar‐recorded and actigraphic activity data, and between LIDS signals and overall hypnogram dynamics (represented by PSG‐INH). (2) Identify the best‐fit single‐harmonic cosine model for each signal. Then, compare the parameters (amplitude, period, phase, and offset) estimated from the different signal types; in particular, compare LIDS‐derived with PSG‐derived parameters to evaluate if the former could be a possible estimate of the latter. (3) Evaluate if the oscillating ultradian patterns and trends found in aggregates of actigraphic LIDS (Winnebeck et al., 2018) would emerge in aggregates of LIDS signals derived from a contact‐free radar sensor.

## 2 METHODS

### 2.1 Data collection

The data used in this work were collected as part of a randomised cross‐over trial meant to evaluate the effect of the light conditions in an acute psychiatric hospital unit at St Olavs Hospital in Trondheim, Norway (Vethe et al., 2021), and are the same data used to develop and evaluate contact‐free sleep/wake classification models in our previous work (there referred to as Dataset 1) (Heglum et al., 2021). The study protocol was approved by the Regional Ethical Committee in Trondheim, (Central Norway; REK: 2017/916) and is registered on the ISRCTN website (reference number 12419665). Written informed consent was obtained from all participants.

The dataset consists of data from 12 healthy young adults (mean age ± SD: 23.0 ± 3.1 years, 5 male) who resided in the otherwise empty hospital ward for a total of 10 days each (the newly constructed unit had not yet opened for regular patient admissions). Each participant wore a Phillips Actiwatch (Actiwatch Spectrum, Philips Respironics Inc., Murrysville, PA) on the non‐dominant wrist for the duration of the study. Each room was outfitted with two radar sensors (XeThru model X4M200, Novelda AS, Oslo, Norway [Novelda AS, 2018]), one embedded in the ceiling and one placed on a nightstand; both recording continuously for the duration of the study. One ceiling radar malfunctioned, leading to some data loss. Each participant also underwent four nights of PSG, manually scored by a specialist in clinical neurophysiology according to the AASM manual for the Scoring of Sleep and Associated Events, version 2.4 Berry et al., (2017). One night of PSG was lost due to an equipment error.

The final dataset contains a total of 47 nights of consecutive recordings from PSG, actigraphy, and radar. Four nights lack ceiling radar data. Additionally, the dataset contains 125 nights of consecutively recorded nightstand radar and actigraphy data, with 117 of these also containing data from the ceiling radar. The study protocol, recording setup, and data preparation is described in greater detail in our previous works (Heglum et al., 2021; Vethe et al., 2021).

### 2.2 Radar signal processing

The radar used in the present work is an impulse radio ultra‐wideband (IR‐UWB) radar, which generates short pulses of radio signals in the 3.1–10.6 GHz frequency band and measures their reflections from objects in the environment. The combination of large bandwidth with high frequencies gives signals with high resolution despite very low power, that easily penetrate soft materials such as clothes and bedding while being reflected by more solid objects such as human bodies (Stone, 1997). The radar digital signal processing (DSP) used in the present work was provided by the manufacturer, and extracts both distance to and velocity of a reflecting object by measuring the time‐of‐flight and the doppler shift of the reflected radio pulses (Novelda AS, 2018). For the closest target where movement is detected above a configurable threshold, small low‐frequency oscillations and larger, faster movements are detected simultaneously, every second, by performing a Fast Fourier Transform over the preceding 20 and 6 s of data, respectively. In the present work, only the larger, faster movements, normalised for distance to target, are used for the calculation of LIDS. In this data, slow and small oscillations such as respiration are greatly dampened if they register above the detection threshold at all. For this reason, although LIDS is a transformation designed to amplify the impact of small signals, no additional steps were taken to eliminate low‐magnitude noise in this work.

### 2.3 Locomotor inactivity during sleep applied to radar data

Locomotor inactivity during sleep (LIDS) is a non‐linear transformation of activity data, meant to emphasise and enhance movement patterns and dynamics of ultradian sleep cycles (Winnebeck et al., 2018). Activity data are aggregated to bins of 10 min duration via summation, and then inverted from activity to inactivity by passing it through a simple inversion resulting in values from 0 to 100; $\text{inactivity}=\frac{100}{\text{activity}+1}$. LIDS is then found by smoothing these inactivity scores via a 30 min centred moving average.

In the present work, we aimed to stay as close as possible to the procedure described by Winnebeck et al. (2018), however, when working with radar data we found a few adaptations to be necessary:

1. A radar does not follow a participant as they move about their environment; whenever the room under observation is empty, the registered activity has a value of zero. The resulting LIDS signal is less meaningful, as it is pulled to high peaks both by actual locomotor inactivity and by an empty room. To rectify this, we utilise the “state” variable output by the radar, which indicates the presence or absence of people in the room (Novelda AS, 2018). Before applying the LIDS inversion formula to a radar activity recording, we set the radar activity in the time epochs where the “state” variable indicates “no presence” to a constant high value (chosen as the median of observed daily maximum activity values for the respective radar positions). This pushes the LIDS value toward zero at these times, representing an assumption that “room empty” means the subject is active and awake.
2. The data output from both radars and actigraphs are in the form of unitless aggregates of activity counts, but the devices operate at different scales; the 1 Hz normalised fast movement data type from the radar is several orders of magnitude larger than the 15 s epoched activity data from the actigraphs. These differences are further compounded when the movement data are summed into 10 min bins. The large absolute values of the radar activity data have the effect of pushing the inactivity formula closer to zero much more aggressively in the presence of movement than the comparatively smaller values from the Actiwatch. To alleviate this, the 10 min bins of aggregated radar data were rescaled to the range [0, 1000] using the MATLAB rescale function before they were passed through the inversion formula. To increase comparability of the sensor types, the same rescaling was applied, in the same way, to the actigraphy data.

### 2.4 Inverted numerical hypnogram

In a PSG hypnogram, sleep stages are usually plotted as categorical variables with wake on top, then descending through REM, N1, N2, to N3. To provide convenient comparison with LIDS, we converted these sleep profiles into inverted numerical hypnograms (PSG‐INH). The sleep stages were given numerical values corresponding to an inversion of their usual positions; Wake = 0, REM = 1, N1 = 2, N2 = 3, and N3 = 4. Then, to correspond with the 10 min resolution of LIDS signals, the 30 s epoch sleep profiles were aggregated into bins of 10 min duration by taking the mean. Finally, the inverted and aggregated sleep profiles were rescaled to match the LIDS signal range of [0, 100] (using the MATLAB rescale function).

### 2.5 Estimating the period of the **LIDS** cycle

Actigraphy‐derived LIDS signals have been used to determine the predominant, or mean, period of the sleep dynamics, by identifying the period of the first‐order cosine model whose parameters (phase, period, amplitude, and offset) minimised the difference between model and data (Winnebeck et al., 2018). We followed this procedure, with some modifications, for all signals (LIDS and PSG‐INH) from the nights with PSG data available. A separate cosine model was fitted for each individual signal, giving four sets of non‐identical parameters for each night of recordings in the dataset.

For the purpose of fitting the cosine models, data were first trimmed to exclude data from outside of the main sleep period; “PSG Lights Off” (set by user marker) to 7 a.m., the set rise time for the participants. The task of finding the best‐fit cosine model was then approached as the nonlinear optimisation problem of finding the four parameters a, b, c, and d (amplitude, period, phase, and offset) that minimise the cost function $f\left(t\right)={\left(y\left(t\right)-\hat{y}\left(t\right)\right)}^{2},$ where $y\left(t\right)$ is the original signal and $\hat{y}\left(t\right)=\text{asin}\left(\frac{2\pi }{b}\left(t-c\right)\right)+d$ is a single‐harmonic sinusoid. The starting points for amplitude, phase, and offset were set to $\text{range}\left(y\left(t\right)\right)$, zero, and $\text{mean}\left(y\left(t\right)\right)$, respectively. To mitigate the risk of the optimisation converging to a local minimum, the problem was then solved 30 times using the MATLAB function fminsearch with starting periods from 30 to 180 min in steps of 5. The set of resultant parameters giving the highest correlation coefficient between signal and cosine model (as given by the MATLAB function corrcoef) was chosen as the best fit, given that the period of this parameter set was between 30 and 180 min.

### 2.6 Average **LIDS** profiles

When individual LIDS signals are averaged over their original timelines the aggregate signal has been seen to be initially rhythmic but quickly dampened by the progressive desynchronisation of many different overlapping periodicities; however, this effect has been counteracted through a procedure for normalising and synchronising the individual LIDS signals prior to averaging (Winnebeck et al., 2018). We followed this procedure for all signals (LIDS and PSG‐INH) from the nights with PSG data available: The timelines for each individual signal was converted from “external” (real) time to an “internal” time for which their mean LIDS period is exactly 90 min by transforming them according to the formula $t_{\mathrm{int}}=\frac{t_{\mathrm{ext}}}{\text{optPer}}\times 90$, where optPer is the period of the best‐fit cosine model. The LIDS signals themselves were then correspondingly resampled from their “external” rate (10 min) to their “internal” rate ($\frac{\text{optPer}}{9}$ min) using the MATLAB resample function. The resampled internal‐time LIDS curves were then phase synchronised by identifying the first peak in their optimal cosine curve, then assigning this point as t=30 min (i.e., bin nr. 3) on a common timeline over which all recordings were placed before the average LIDS profile was calculated from the resulting matrix.

### 2.7 Statistical analysis

Similarity between LIDS signals derived from different types of activity data (actigraphy, nightstand‐, and ceiling radars), and between LIDS signals and PSG‐INH, was evaluated using the Pearson correlation coefficients (*r*) between the signals. For each set of concurrent recordings from each participant, the MATLAB function corrcoef was used with LIDS signals and PSG‐INH (where available) as columns and concurrent 10 min bins as rows. When PSG‐INH was not available, a series of NaN (Not a Number) values were generated to replace it. Other missing data were also replaced by NaN. Corrcoef was then instructed to compute each two‐column correlation coefficient on a pairwise basis, so that if one of the two columns contained a NaN, that row was omitted (MathWorks, 2022). The median of the resulting 4 × 4 matrices of correlation coefficients were then found for each participant, before finding the medians and quartiles of the individual values over the dataset. To evaluate within‐subject variability, the range of all *r* values for each participant were also calculated. The minimum and maximum values of within‐subject range of *r* were recorded, as well as their medians over the participants.

The Pearson correlation coefficient was also used to evaluate the goodness‐of‐fit of the single‐harmonic cosine models (GF‐*r*, “goodness‐of‐fit” correlation). The corrcoef function was used to calculate the GF‐*r* values between each signal (LIDS from three sensors and PSG‐INH) and their best‐fit model. The total number of nights for which the correlation between signal and best‐fit cosine model was at least moderately strong (*r* > 0.4) were counted. The medians and quartiles of GF‐*r* were calculated. To evaluate within‐subject variability, medians, and quartile values of within‐subject GF‐*r* range were recorded.

The means and standard deviations of the four estimated parameters (amplitude, period, phase, and offset) of the best‐fit cosine models were calculated for each signal type. The bias of LIDS parameters compared with PSG‐INH were calculated, including 95% confidence intervals, *p* values from paired‐sample Student's *t*‐tests on the hypothesis of zero bias, and 95% Limits of Agreement (calculated as bias ±1.96 × SD).

Mixed model analysis was performed to evaluate systematic differences between devices and overall trends of LIDS‐ and PSG‐INH over time. Only the nights with a simultaneous PSG recording available were included. A linear mixed effect regression model was computed with signal (LIDS and PSG‐INH) over normalised timelines as the target variable. As fixed effects we included device type, cycle number, and sex (nested by device), with PSG‐INH as the reference device and female as the reference sex. As random intercepts we included subject, night nested within subject, and cycle nested within night nested within subject. Finally, the cycle within each individual was included as a random slope.

## 3 RESULTS

**FIGURE 1:**
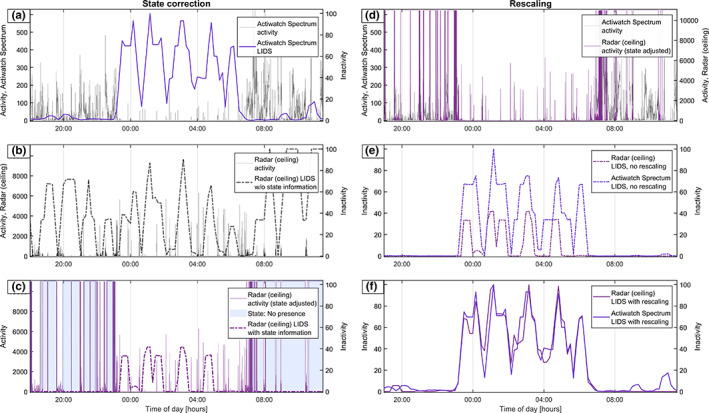
Application of the LIDS transform to simultaneously recorded radar and actigraphy movement data from one participant over one night, with step‐by‐step illustration of the preprocessing steps and their effect. (a) The Actiwatch Spectrum follows the participant throughout the day, so the resting period at night is clearly defined and the corresponding LIDS immediately meaningful. (b) The radar remains stationary, with “zero activity” throughout large portions of the day when the room is empty. Without including state information indicating the presence or absence of a person in the room where the radar is continuously recording, the resulting LIDS signal is not meaningful. (c) By replacing the activity values during the “no presence” epochs with the maximum value of radar activity, LIDS is forced toward zero at these times. The resulting signal is meaningful in terms of “rest and activity in the specific location being measured”. (d) The radar and the actigraph originally operate at different scales, as shown by the left (actigraph) and right (radar) *y*‐axes. (e) The difference in scales cause the radar‐derived LIDS to be pushed more aggressively toward zero in the presence of activity. (f) By rescaling the data prior to transformation, two initially different types of movement data produce comparable LIDS signals. LIDS, locomotor inactivity during sleep

**FIGURE 2:**
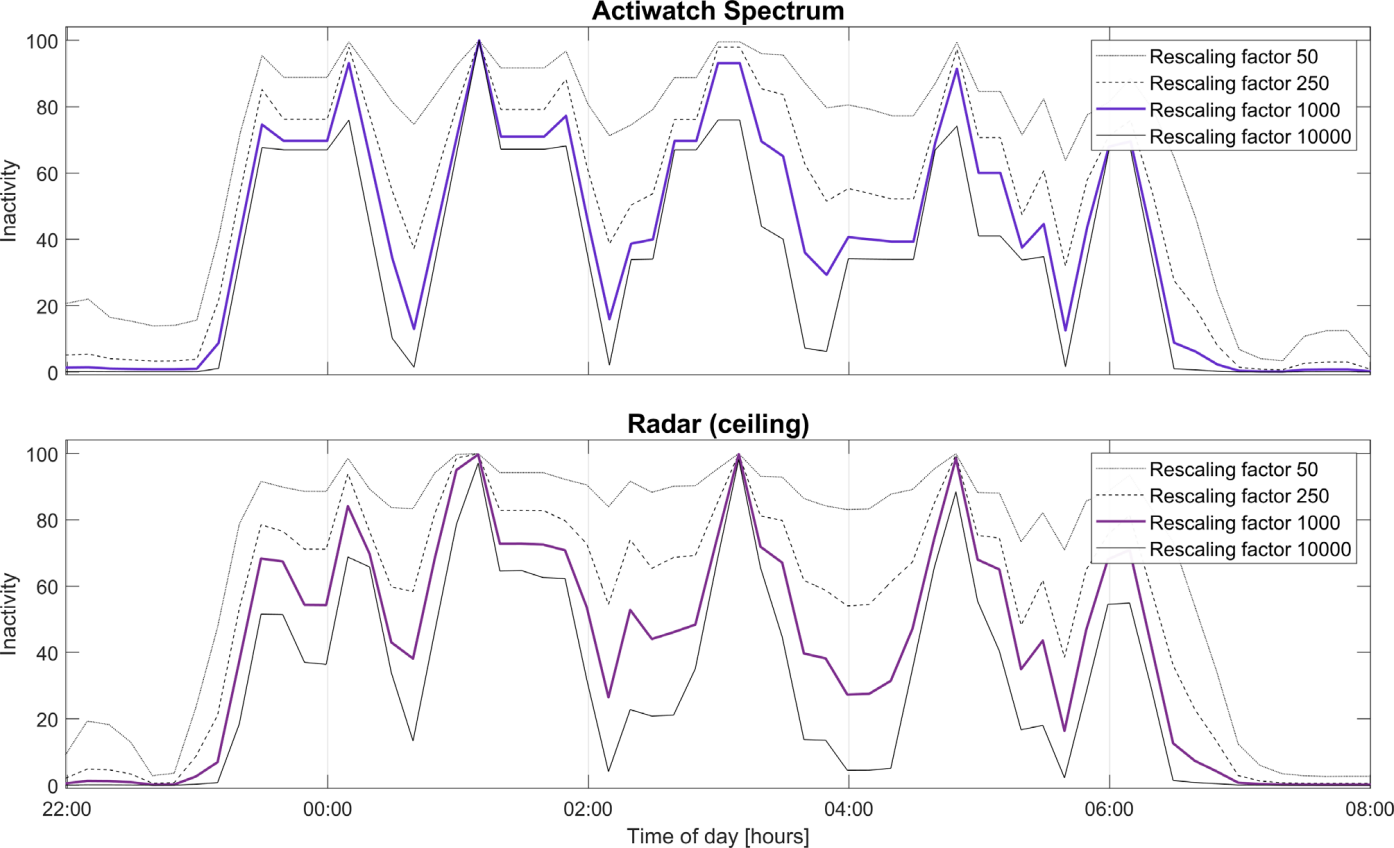
The effect of different rescaling factors on LIDS signals derived from simultaneously recorded radar and actigraphy movement data from one participant over one night. A lower rescaling factor will reach higher values of inactivity more easily and will resist being pushed down in the presence of activity. A higher rescaling factor will react more quickly to activity. LIDS, locomotor inactivity during sleep

**FIGURE 3:**
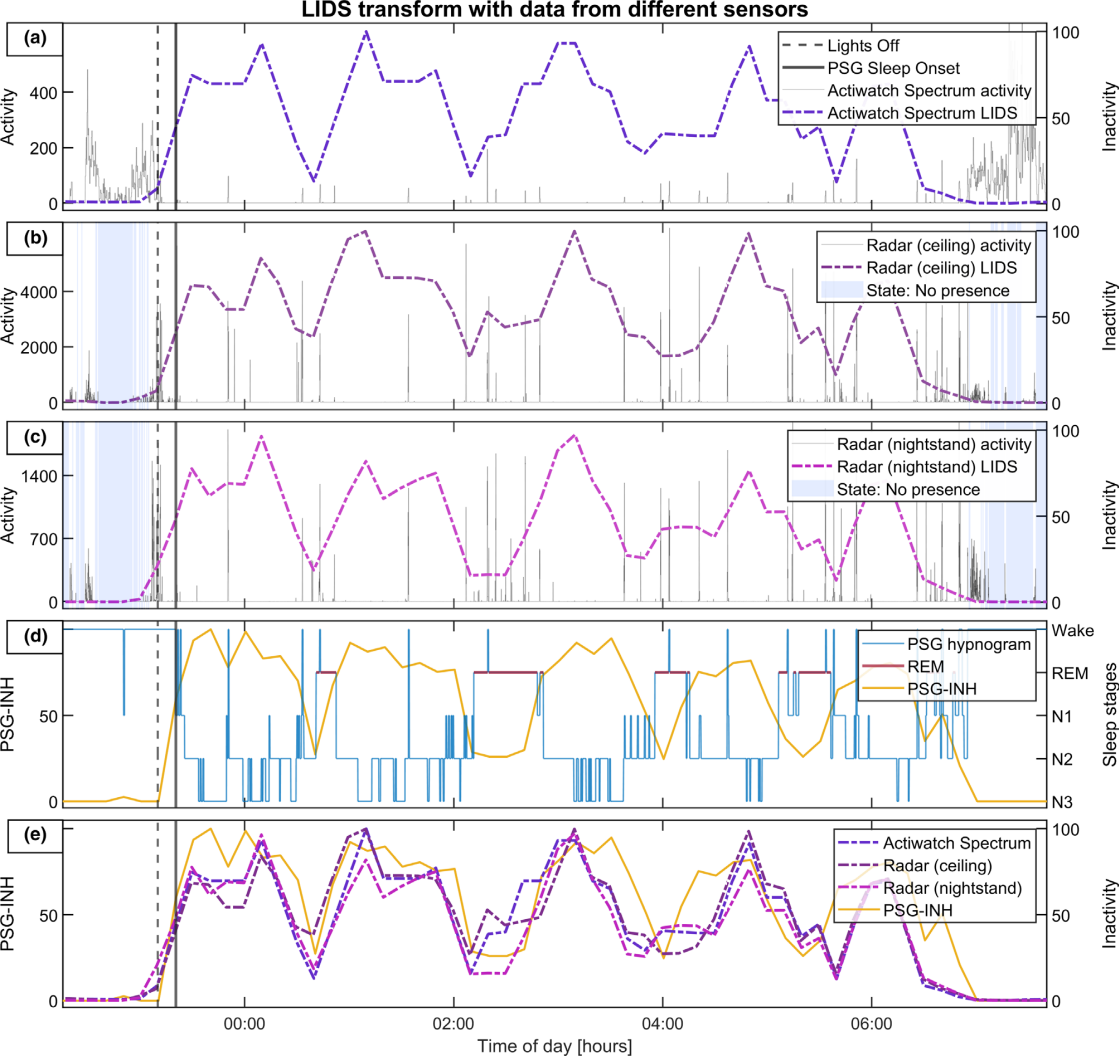
Parallel plots of simultaneous recordings with three activity sensors and PSG, from one participant over one night. The LIDS transform was applied to the activity sensors, and the PSG hypnogram was inverted and averaged into 10 min bins, then rescaled to a range of [0, 100], to correspond with the temporal resolution and numerical range of the LIDS transform. The final subplot shows this inverted numerical hypnogram (PSG‐INH) plotted together with the three sensor‐derived LIDS signals. PSG, polysomnography; LIDS, locomotor inactivity during sleep

Figures 1, 2, 3 illustrate the process of the LIDS transform, using data from one night of simultaneous recordings. In Figure 1, actigraphy‐derived LIDS is compared with radar‐derived LIDS, with step‐by‐step application of the two adaptations made to the process: state correction and rescaling. We see how, before state correction, radar‐LIDS is pulled upwards both by actual locomotor inactivity and by periods where the room is empty. After state correction the radar‐LIDS is pushed to zero at those times, resulting in a more meaningful signal with dynamics somewhat comparable to actigraphy‐LIDS. However, the magnitude of this radar‐LIDS remains significantly lower than its actigraphy‐derived counterpart. This is due to the differences in the scales the original data; the high absolute values of the radar activity data cause any activity to push radar‐LIDS toward zero too aggressively. This effect is alleviated by rescaling the aggregated activity data from different sensors to the same range prior to transformation. Figure 2 illustrates the effect of the choice of rescaling range. Figure 3 shows activity data and LIDS from all three activity sensors (actigraphy, ceiling, and nightstand radar), plus the corresponding PSG hypnogram for this night and its transformed and inverted form (PSG‐INH). In the final subplot of Figure 3, the three LIDS signals and PSG‐INH are plotted together.

The matrix of correlations between LIDS signals and PSG‐INH can be seen in Table 1. Median [Q1, Q3] *r* between PSG‐INH and nightstand and ceiling radars were 0.83 [0.82, 0.87] and 0.80 [0.76, 0.85], respectively, and 0.78 [0.70, 0.82] between PSG‐INH and actigraphy. Between the nightstand and ceiling radar positions median *r* was 0.94 [0.91, 0.95], and respectively, 0.88 [0.84, 0.90] and 0.84 [0.80, 0.87] between radars and actigraphy. Table 1 also includes median, minimum, and maximum of within‐subject range.

**TABLE 1:** Correlation matrix for LIDS^a^ signals derived from all available concurrent recordings of actigraphy, nightstand‐, and ceiling radars, and PSG‐INH^b^

Median *r* [Q1, Q3] within‐subject range (median [min, max])	Nightstand radar LIDS (*n* = 125)	Ceiling radar LIDS (*n* = 117)	Actigraphy LIDS (*n* = 125)
PSG‐INH (*n* = 47)	0.83 [0.82, 0.87] (0.10 [0.05, 0.26])	0.80 [0.76, 0.85] (0.08 [0.02, 0.23])	0.78 [0.70, 0.82] (0.11 [0.03, 0.24])
Nightstand radar LIDS (*n* = 125)	‐	0.94 [0.91, 0.95] (0.10 [0.03, 0.42])	0.88 [0.84, 0.90] (0.15 [0.08, 0.28])
Ceiling radar LIDS (*n* = 117)	‐	‐	0.84 [0.80, 0.87] (0.17 [0.09, 0.43])

*Note*: Median, upper, and lower quartiles of within‐subject PCC^c^ (*r)* medians over the dataset. Within‐subject variability shown as median within‐subject range of *r* over all participants, as well as minimum and maximum within‐subject range of *r* of any participant.

The smallest of row and column *n*'s shows how many concurrent recordings were available for any given combination of sensors^d^.

^a^ LIDS, locomotor inactivity during sleep.

^b^ PSG‐INH, polysomnography – inverted numerical hypnogram.

^c^ Pearson correlation coefficient.

^d^ Excepting ceiling radar and PSG, for which the number of concurrent recordings was *n* = 43 because of a malfunctioning sensor.

Statistics related to the cosine model fittings are reported in Table 2. Of the 184 total model fittings performed, 47 for each of the four kinds of signal (LIDS from three sensors and PSG‐INH), GF‐*r* was >0.4 for 176 (96%). The median [Q1, Q3] GF‐*r* between signal and best‐fit cosine model was 0.56 [0.50, 0.63] for PSG‐INH, 0.56 [0.47, 0.63] for actigraphy, and 0.53 [0.47, 0.60] and 0.54 [0.46, 0.61] for ceiling and nightstand radars, respectively.

**TABLE 2:** Goodness‐of‐fit statistics for single‐harmonic cosine models fitted to concurrently recorded PSG‐INH^a^ and LIDS^b^

	PSG‐INH (*n* = 47)	Nightstand radar LIDS (*n* = 47)	Ceiling radar LIDS (*n* = 43)	Actigraphy LIDS (*n* = 47)
*GF‐r* ^*c*^ >0.4	47/47 [100%]	44/47 [94%]	40/43 [93%]	45/47 [96%]
*GF‐r* median [Q1, Q3] within‐subject range (median [min, max])	0.56 [0.50, 0.63] (0.15 [0.06, 0.25])	0.53 [0.47, 0.60] (0.18 [0.10, 0.42])	0.54 [0.46, 0.61] (0.14 [0.01, 0.38])	0.56 [0.47, 0.63] (0.21 [0.11, 0.36])

*Note*: Cosine models were fitted to data from the nights where the participants underwent PSG in addition to being recorded with two radars and actigraphy. GF‐*r* between the model and the four input data time series were calculated.

GF‐*r* was above 0.4 for 96% of the 184 fittings performed in total. Within‐subject variability is shown through the median of the within‐subject range of GF‐*r* for all participants, as well as the minimum and maximum within‐subject range of GF‐*r* found in the dataset.

^a^ PSG‐INH, polysomnography – inverted numerical hypnogram.

^b^ LIDS, locomotor inactivity during sleep.

^c^ GF‐*r*, goodness‐of‐fit correlation (Pearson).

**FIGURE 4:**
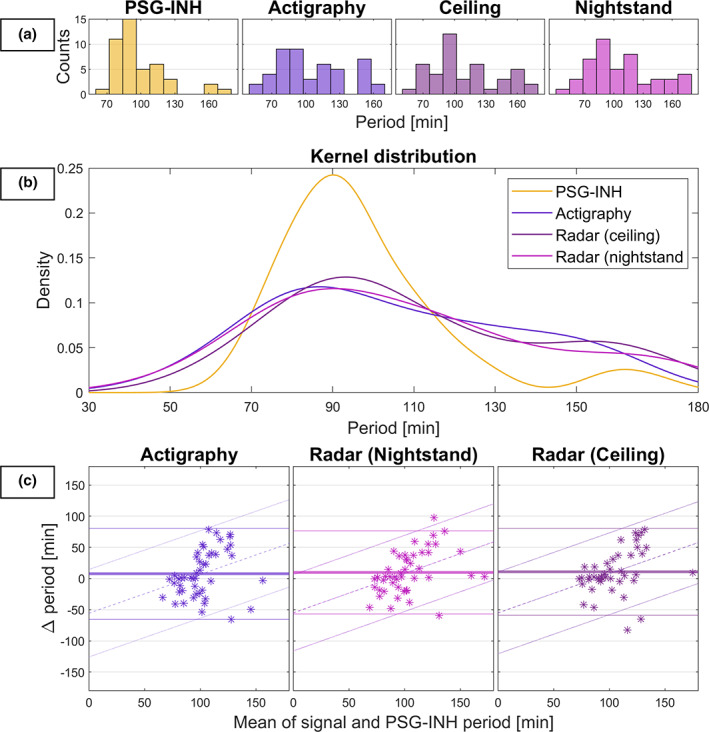
Periods of the best‐fit cosine models for PSG‐INH and LIDS from actigraphy, ceiling radar, and nightstand radar, estimated for all nights with a concurrent PSG recording available. (a) Histograms of cosine‐estimated periods. (b) Kernel distributions of cosine‐estimated periods. (c) Bland–Altman plots comparing LIDS‐estimates to PSG‐INH estimates, including slopes (where significant at the 5% level) estimated according to the regression approach for nonuniform differences (Bland & Altman, 1999)

Table 3 summarises the four parameters of the cosine models (amplitude, period, phase, and offset), and compares the PSG‐INH parameters with those of the LIDS signals. Figure 4 illustrates the estimated periods specifically, including histograms and kernel distribution of estimated periods, and Bland–Altman plots of LIDS compared with PSG‐INH. The mean of the periods of the best‐fit cosine models was 97.8 min for PSG‐INH, 105.5 for actigraphy, and 107.8 and 110.5 for the nightstand and ceiling radars, respectively. Paired‐sample Student's *t*‐tests did not reveal systematic bias (at the 5% significance level) between the amplitude, period, and phase of cosine models fitted to LIDS signals compared with those fitted to PSG‐INH; however, a 6% significance level would reject the hypothesis for the radar periods, and the period and phase confidence intervals and limits of agreement were large for all period and phase estimates. Comparing the offsets of LIDS and PSG‐INH is not meaningful as the numerical PSG coding is arbitrary.

**TABLE 3:** Cosine fit model parameters

	PSG‐INH^a^ (*n* = 47)	Nightstand radar LIDS^b^ (*n* = 47)			Ceiling radar LIDS (*n* = 43)			Actigraphy LIDS (*n* = 47)		
	Mean (SD)	Mean (SD)	Overall bias (95% CI) [*p*‐value]	95% limits of agreement	Mean (SD)	Overall bias (95% CI) [*p*‐value]	95% limits of agreement	Mean (SD)	Overall bias (95% CI) [*p*‐value]	95% limits of agreement
Amplitude [0–100]	19.2 (2.6)	18.0 (4.4)	−1.1 (−2.4, 0.2) [0.086]	[−9.9, 7.6]	19.3 (5.3)	0.2 (−1.4, 1.7) [0.816]	[−9.8, 10.1]	20.4 (5.1)	1.2 (−0.2, 2.6) [0.085]	[−8.0, 10.4]
Period [min]	97.8 (21.9)	107.8 (33.8)	10.0 (0.0, 20.0) [0.05]	[−56.7, 76.7]	110.5 (32.4)	10.8 (−0.2, 21.8) [0.054]	[−59.1, 80.6]	105.5 (30.4)	7.7 (−3.3, 18.6) [0.164]	[−65.4, 80.8]
Phase [min]	27.9 (16.5)	36.6 (29.7)	8.7 (−1.7, 19.1) [0.098]	[−60.5, 77.9]	35.4 (30.3)	7.0 (−2.6, 16.7) [0.149]	[−54.5, 68.6]	32.3 (26.3)	4.3 (−5.2, 13.9) [0.365]	[−59.4, 68.1]
Offset [0–100]	65.4 (3.9)	53.2 (6.6)			48.3 (7.5)			49.9 (8.0)		

*Note*: Parameters of the cosine models fitted to data from the nights where the participants underwent concurrent recordings of actigraphy, nightstand‐, and ceiling radars, and PSG^c^.

LIDS‐derived parameters are compared with PSG‐INH. Positive values of bias indicate overestimation by the LIDS‐parameter (*p*‐values from Student's *t*‐test on the null hypothesis that the bias is zero). Limits of Agreement was calculated as [bias ±1.96 × SD].

^a^ PSG‐INH, polysomnography – inverted numerical hypnogram.

^b^ LIDS, locomotor inactivity during sleep.

^c^ PSG, polysomnography.

**FIGURE 5:**
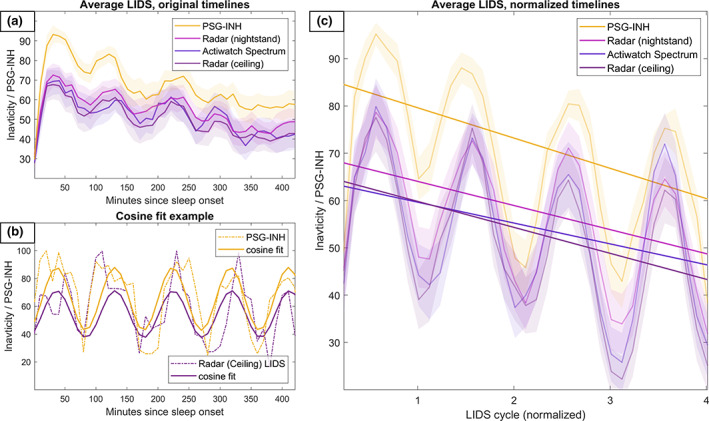
Average LIDS and PSG‐INH profiles over the nights with PSG recordings available, before and after normalisation of the timeline. (a) Average of the LIDS (and PSG‐INH) signals, ±95% confidence intervals shaded. (b) An example of the optimal cosine curves fitted to a ceiling radar LIDS signal and the PSG‐INH of its concurrently recorded PSG hypnogram. (c) Average of the LIDS (and PSG‐INH) signals (±95% CI), and linear mixed effects model fits, over timelines normalised to individual predominant LIDS periods as estimated by the corresponding optimal cosine curves. LIDS, locomotor inactivity during sleep; PSG, polysomnography; PSG‐INH, inverted numerical hypnogram

Period estimation and LIDS profile averaging are illustrated in Figure 5; first the simple averaging of all LIDS vectors with concurrent PSG recordings, then an example of cosine models fitted for the ceiling radar LIDS and the PSG‐INH over a single night. Finally, the figure shows the average LIDS and PSG‐INH profiles formed after first normalising the timelines to their internal rate based on the calculated predominant periods and synchronising the signals by their first peak. These profiles are shown along with lines illustrating the result of linear mixed model analysis of the cycle‐normalised profiles. The slopes of these lines, representing the average decline of inactivity (or PSG‐INH) per cycle, were found to be −6.4 units per cycle for PSG‐INH, and −5.5, −5.1, and −4.4 units per cycle for LIDS from ceiling radar, Actiwatch Spectrum, and nightstand radar, respectively. All upper and lower 95% confidence bounds for slopes were overlapping. The ceiling radar slope overlapped zero. Compared with the reference PSG‐INH intercept at 86.5, the ceiling radar, Actiwatch Spectrum, and nightstand radar had intercepts of −20.2, −18.9, and −14.5, respectively. The upper and lower bounds of all LIDS intercepts were overlapping. Males exhibited lower levels of LIDS and PSG‐INH than females, except for in the ceiling radar LIDS. The results of the mixed model analysis are summarised in Table 4.

**TABLE 4:** Mixed model analysis of LIDS^a^ and PSG‐INH^b^ over normalised timelines

Fixed effects			
	Estimate	*p*	95% CI
(Intercept)	86.5	<0.001	[83.1, 89.9]
Cycle	−6.4	<0.001	[−7.3, −5.5]
Sex	−4.4	0.01	[−7.5, −1.2]
Radar (Nightstand)	−14.5	<0.001	[−18.2, −10.8]
Radar (Ceiling)	−20.2	<0.001	[−24.1, −16.4]
Actiwatch Spectrum	−18.9	<0.001	[−22.6, −15.2]
Cycle: Radar (Nightstand)	1.3	0.02	[0.2, 2.4]
Cycle: Radar (Ceiling)	0.9	0.12	[−0.2, 2.1]
Cycle: Actiwatch Spectrum	2.0	<0.001	[0.9, 3.1]
Sex: Radar (Nightstand)	−4.8	<0.001	[−7.8, −1.7]
Sex: Radar (Ceiling)	−0.6	0.71	[−3.7, 2.5]
Sex: Actiwatch Spectrum	−6.1	<0.001	[−9.1, −3.0]
Random effects			
		Estimate [SD]	95% CI
PID^c^(Night[Cycle])	(Intercept)	1.4	[1.2, 1.6]
PID(Night)	(Intercept)	1.5	[0.4, 5.7]
PID	(Intercept)	5.8	[4.9, 6.8]
	cycle	0	
Residual		23	[22.7, 23.4]

*Note*: Target variables were LIDS and PSG‐INH over normalised timelines, with PSG‐INH as the reference. “Cycle” refers to LIDS (or PSG‐INH) cycle. Data were analysed in bins of normalised duration (internal time = 90 min), starting from PSG sleep onset and ending at the prescribed rise time for the participants. Reference sex was female. Only nights with concurrent PSG recordings available were included.

*n* = 184 nights of recordings total (47 per device [43 with ceiling radar], 12 subjects). Number of observations: 7453.

^a^ LIDS, locomotor inactivity during sleep.

^b^ PSG‐INH, polysomnography – inverted numerical hypnogram.

^c^ PID, participant identification.

**FIGURE 6:**
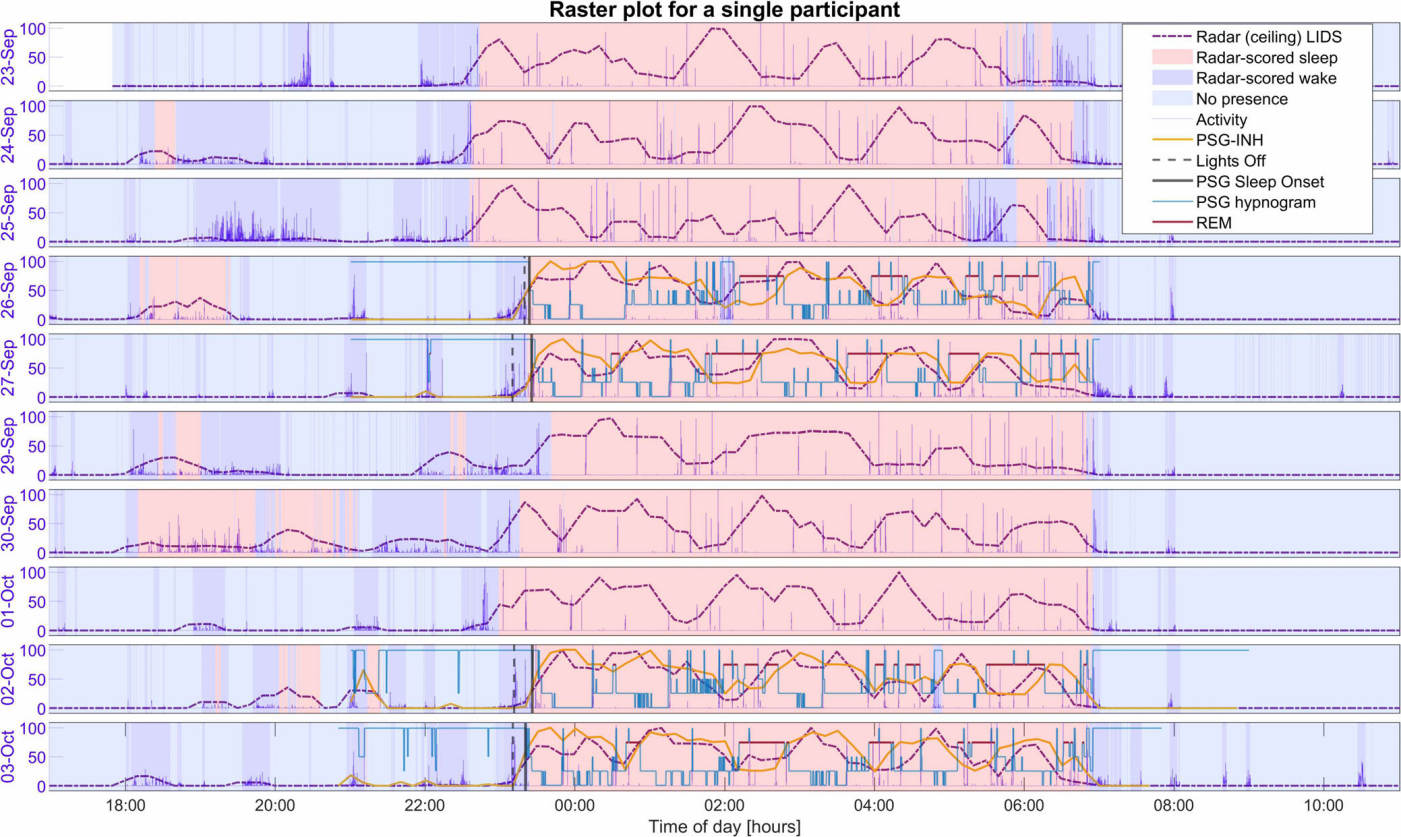
Temporal raster plot generated from ceiling‐radar data for a single participant over the recording period, as an example to show that LIDS plotted together with activity data may provide additional information about sleep dynamics. Background colours indicate the no presence state, and the sleep/wake states is scored by the real‐time model from our previous work (Heglum et al., 2021). PSG hypnograms and PSG‐INH signals are included where available. LIDS, locomotor inactivity during sleep; PSG, polysomnography; PSG‐INH, inverted numerical hypnogram

Figure 6 is an example of how LIDS signals can be plotted together with activity data to provide additional information about sleep dynamics in a traditional actigraphy‐style raster plot. The figure shows data for all nights for a single participant, also including PSG information where available as well as sleep–wake classification based on the ceiling radar using a model developed in our previous work (Heglum et al., 2021).

The full results of the mixed model analysis as well as a complete set of figures in the style of Figures 3 and 6 for each recording and each participant in the dataset can be found attached to the Appendix S1 of the present work.

## 4 DISCUSSION

After a few adaptations, the LIDS transform was applicable to contact‐free radar measurements of body movement. LIDS derived from radar data were very similar to actigraphy‐derived LIDS. Both also highly correlated with down‐sampled PSG hypnograms (PSG‐INH), indicating a distinct relationship between the macroscopic dynamics of polysomnographically measured ultradian REM/NREM sleep cycles and the patterns formed by activity and inactivity during sleep as revealed by the LIDS transformation. LIDS (radar‐ and actigraphy‐derived) and PSG‐INH both exhibited cycles that were distinct and stationary enough that a single‐harmonic cosine function could adequately model the signal, and key parameters of these models (amplitude, period, and phase) were not significantly different between PSG‐INH and LIDS. Mixed‐model analysis of aggregated LIDS and PSG‐INH, signals revealed trends of decline per cycle similar to those observed in a previous investigation of aggregated LIDS in a large dataset (more than 16,000 nights of 500 subjects) (Winnebeck et al., 2018), demonstrating that trend analysis of LIDS patterns on a group‐level can be done also in smaller studies, and that trends in LIDS may be useful reflections of trends in REM/NREM cyclicity.

While LIDS do not assess sleep architecture in the traditional sense, the simplicity and transparency of the method means that it can enable the study of sleep dynamics while circumventing some of the challenges of more complex approaches. For example, the fit statistics of the cosine modelling process, specifically the correlation between signal and best‐fit model, may be useful indicators of the strength and stability of the sleep cycles – and conversely, if a cosine model of acceptable fit cannot be found, it may be an indicator of some abnormality. Moreover, while Sundararajan et al. found that LIDS on its own probably lacks the discriminatory strength needed to classify sleep stages, they did report that LIDS had significant value as a feature for both sleep/wake‐ and non‐wear detection from actigraphy (Sundararajan et al., 2021); it may consequently be a useful feature to consider when designing more complex sleep analysis tools using movement data.

LIDS seems to represent a basic physiological relation between movement cycling and sleep stage cycling and may therefore be capable of providing clinically relevant surrogate markers of some subset of PSG‐derived measures. It has been reported to correlate with several polysomnographic sleep parameters, such as micro‐arousals, REM density, slow eye movements, delta amplitude density, and theta activity (Winnebeck et al., 2018), which again have been linked to incidence, course, and treatment of several disorders (such as insomnia, mood disorders, schizophrenia, and other psychiatric disorders) and to other health‐related phenomena such as cognitive performance and longevity (Drews et al., 2018; Feige et al., 2013; Leary et al., 2020; Lechinger et al., 2020; Monica, Johnsen, Atzori, Groeger, & Dijk, 2018; Palagini, Baglioni, Ciapparelli, Gemignani, & Riemann, 2013; Van Someren, 2021; Weinhold et al., 2021). The parameters of the cosine‐models may be one way to reflect this physiological relation. For example, the period of the cosine model may be an estimator of average sleep cycle length, which has been linked to cognitive decline in the elderly (Suh et al., 2019). However, Table 3 and Figure 4 indicate that the LIDS‐derived periods estimated with the present methodology may be biased toward higher values, compared with periods estimated from PSG‐INH. Moreover, the Bland–Altman plots of Figure 4 indicate that this bias may be nonuniform. Different parameter estimates between the four signals do not necessarily reflect physiological differences, but an assessment of this variability is useful for evaluation of method consistency. For instance, the wide limits of agreement suggest that the tentative application of this method in individual subjects is limited in its present form. More in‐depth harmonic analysis of the signals, which should include more than a single periodic component and test against PSG‐periods extracted manually from the hypnogram (as opposed to estimated automatically from the PSG‐INH), may help reveal if and how the quality and consistency of the estimates can be enhanced.

Generalising the LIDS method to work with data from a contact‐free sensor provides important flexibility, as such sensors have a clear advantage in situations where on‐body sensor equipment is not desirable or feasible. UWB radars in particular are a novel tool in sleep research that may have further advantages in terms of data richness and potential. A recent attempt at sleep stage classification using wrist‐worn accelerometer data concluded that it lacked sufficient discriminative features necessary for the task (Sundararajan et al., 2021); for the radar this is not necessarily true. It can detect a comparatively more complex set of movements, such as macroscopic limb and body movements, chest movements induced by respiration, and even heart beats transmitted to the body surface (Khan, Ghaffar, Khan, & Cho, 2020; Lee et al., 2018; Wisland et al., 2016). Radars have achieved favourable results in sleep‐stage classification based on artificial intelligence (Toften, Pallesen, Hrozanova, Moen, & Grønli, 2020), and have shown promise for detection and analysis of sleep disordered breathing (Kang et al., 2020; Tran, Al‐Jumaily, & Islam, 2019). In acute psychiatry it has been argued that permanently integrated radars in the walls or ceiling of hospital wards could become a core feature in “chronobiologically informed inpatient environments”, built to improve non‐invasive treatment of severely ill psychiatric inpatients (Drews, Scott, Langsrud, Vethe, & Kallestad, 2020; Scott, Langsrud, Goulding, & Kallestad, 2021; Vethe et al., 2021).

As the LIDS method was generalisable from one body movement sensor to another, by inference it may also be generalisable to other body movement sensors, such as raw data actigraphs, mattress sensors, infrared cameras, and so forth. Of the adjustments made in the present work, state correction would be necessary only for a position‐locked sensor, whereas rescaling would be a generalising step recommended for any device.

Further investigation is required, however. The LIDS transformation process includes several preprocessing steps and parameter choices that should be thoroughly evaluated and standardised. Different rescaling factors, for example, will emphasise different aspects of the signal. As illustrated in Figure 2, a lower rescaling factor reaches higher values of inactivity more easily and will resist being pushed down in the presence of activity, whereas a higher rescaling factor will react more quickly to activity, resulting perhaps in lower‐amplitude LIDS signals with a higher dynamic range. The present work has not established an optimal value for the rescaling – it may be the case that there is none, and that this factor should be a tunable parameter. For example, if a significant cosine function cannot be found for the signal at rescaling factor 1000, it may be possible to find one at factor 10,000. Careful application of this technique may enable dynamic analysis of sleep data from a wider population than otherwise. For position‐locked sensors, the optimal replacement value for no‐presence times should also be calibrated; it must be large enough to consistently push the LIDS signal to zero during these times, but should avoid affecting the ratio of activity to inactivity in the signal. Sundararajan et al. circumvented the low resolution of LIDS by making the 10 min bins themselves moving averages (Sundararajan et al., 2021), producing a smoother signal with a resolution down to the resolution of the original data; this is an interesting alteration that should be investigated and perhaps made standard. The bin length itself is another important choice – the present work chose 10 min bins based on the method proposed by Winnebeck et al. but have also tested other bin lengths and observed that they have a rather large effect on the resulting dynamics – 5 min bins form very fast and “aggressive” dynamics, while 15 min bins were sluggish and slow. A thorough analysis of the effect of bin length was deemed out of scope for the present work but would be an interesting avenue for further investigation. Moreover, LIDS signals can be used to derive several metrics; goodness of fit to a cosine model, mean period of oscillations, cycle‐by‐cycle amplitude decline, etcetera, but the utility of these requires examination. Extensive testing of different LIDS calculation approaches and the usefulness of different LIDS derived parameters should be conducted.

A limitation of the present study is the small size and lack of diversity of the dataset; with only young, healthy sleepers represented, conclusions regarding the utility of LIDS as a predictor of PSG parameters in a clinical setting cannot be drawn. Investigation of LIDS in a more diverse population will be necessary. An important limitation of the LIDS technique itself is that since the transformation is designed to enhance the impact of small signals, it also carries an inherent risk of noise‐amplification. Even a small amount of low‐amplitude noise can have a very large impact on LIDS, as it would be aggregated in the long activity bins and result in erroneously low LIDS values after inversion.

In summary, we have described how to implement the LIDS method with a contact‐free radar sensor. We have observed that LIDS signals carry information on ultradian cycling, both for group aggregates and on the individual level. We argue that our method might be generalisable to other innovative sleep monitoring devices, and that this simple and fully transparent sleep analysis tool could aid in further improving sleep monitoring in both clinical and real‐life settings.

## AUTHOR CONTRIBUTIONS

HSAH and DV had main responsibility for data collection. HSAH did data management and analysis and had overall responsibility for drafting the paper. HJD contributed substantially to the Discussion. ME preformed all polysomnographic scorings. ME, HK, and TS have provided essential mentorship, guidance, and support. HK and KL have been the main drivers in planning the new hospital and establishing related projects, including this one. All authors have revised the article and accepted the final version.

## CONFLICT OF INTEREST

This project is part of the Industrial PhD Scheme of the Research Council of Norway (project no: 284063) and is thus jointly funded by the Research Council and by Novelda AS, who developed the radar used in this work. As a candidate in this Scheme the corresponding author (HSAH) is considered an employee both of Novelda AS and the Norwegian University of Science and Technology (NTNU). None of the other authors are affiliated with Novelda AS, and Novelda AS has not participated in the decision to publish these data beyond the affiliation of and decisions made by the corresponding author.

## Supporting information


**Appendix S1** Supporting Information

## Data Availability

Some de‐identified data that underlie the results reported in this Article may be made available to researchers from accredited research institutions. Access to data will be limited to investigators who provide a methodologically sound proposal and will be limited to a specified time period (commencing about 3 months after publication of this Article and ending after 5 years). To ensure compliance with the General Data Protection Regulation, data processing must be covered by the European Commission's standard contractual clauses for the transfer of personal data, which must be signed by the data requesters. Proposals and requests for data access should be directed to the corresponding author.
