# Supplementary material for: Contact‐free radar recordings of body movement can reflect ultradian dynamics of sleep
Source: J Sleep Res. 2022 Jul 6;31(6):e13687. doi: 10.1111/jsr.13687 (PMC9786343; doi:10.1111/jsr.13687)
Supplement: Supplementary file 1 — Appendix S1 Supporting Information [file JSR-31-e13687-s001.zip › JSR_13687_R1_Supporting Information.pdf]

Contact-free radar recordings of body movement can reflect ultradian dynamics of sleep

Supporting Information

Hanne Siri Amdahl Heglum[1,2], Henning Johannes Drews[3,4], Håvard Kallestad[3, 5], Daniel Vethe[3, 5], Knut Langsrud[3, 5], Trond Sand[1, 6], Morten Engstrøm[1, 6]

[1] Department of Neuromedicine and Movement Science, Faculty of Medicine and Health Sciences, Norwegian University of Science and Technology (NTNU), Trondheim, Norway

[2] Novelda AS, Trondheim, Norway

[3]Department of Mental Health, Norwegian University of Science and Technology, Trondheim, Norway

[4]Department of Public Health, University of Copenhagen, Copenhagen, Denmark

[5]Division of Mental Health Care, St.Olavs University Hospital, Trondheim, Norway

[6]Department of Neurology and Clinical Neurophysiology, St.Olavs University Hospital, Trondheim, Norway

Corresponding author:

Hanne Siri Amdahl Heglum

Email: hanne.s.a.heglum@ntnu.no

Table of Contents

Finding the best-fit cosine model 2
Code examples 3
Mixed model analysis results 8
Complete figure sets 8
  Figure 3.pdf 8
  Figure 6.pdf 8
References 8
Table S1. Mixed model analysis of LIDS and PSG-INH over normalized timelines 9

Finding the best-fit cosine model - example

Finding the best-fit cosine models involve estimating the four parameters a (amplitude), b (period), c (phase), and d (offset), of a single-harmonic sinusoid:

$$\hat{y}(t) = a\, sin\left(2\pi\left(\frac{1}{b}t + c\right)\right) + d$$

Winnebeck et.al. used an approach based on iterative fitting of sinusoid to LIDS, trying thirty different periods, from 30 to 180 minutes in steps of 5, and choosing the best of the resulting models; with maximal Munich Rhythmicity Index (MRI = range of oscillation x r) as best-fit criterion (Winnebeck, Fischer, Leise, & Roenneberg, 2018). In this work, we instead use thirty periods from 30 to 180 minutes in steps of 5 as starting points for a nonlinear optimization problem; this allows periods to take on values outside of the 5-minute intervals used as starting points. For the other three parameters, we choose as starting points amplitude = range of the input signal, phase = 0, and offset = mean of input signal. For cost function, we choose Least-Squares. For the optimization criterion, we chose the correlation *r* between signal and model, given that the period of the highest correlated model is between 30 and 180 minutes.

After the best-fit parameter set is identified, two additional steps are taken to ensure reasonable values. If the amplitude is negative, the value is inverted, and the phase is shifted by one half period. If the phase is negative or larger than the period, it is shifted to the functionally equivalent phase that is positive and within one period of zero.

Consider the example signal in Figure S1:

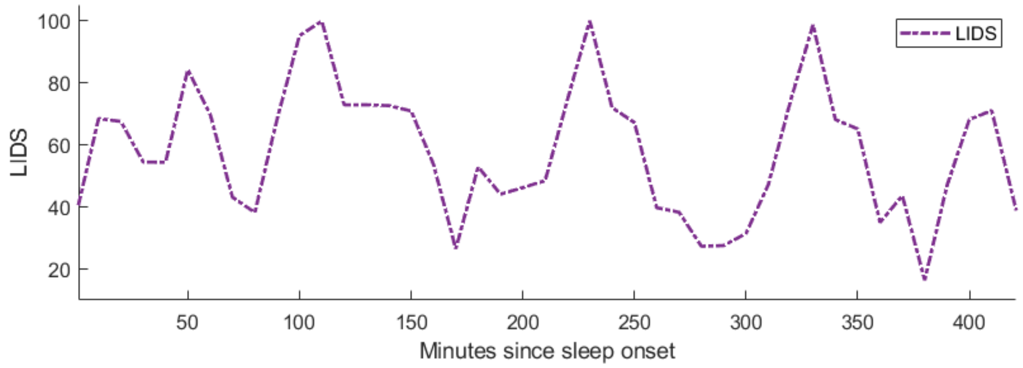

Figure S1: Input signal example.

For this signal, starting parameters for amplitude, phase, and offset are [95.64, 0, 54.73]. The optimization procedure is run with a total of thirty-one starting periods, from 30 to 180 in steps of five. The results of every optimization are listed in Table S1. Identical (or functionally identical) results

represent different local minima for the objective function. In this case, the thirty-one optimizations converged to a total of six different parameter sets, representing six candidate cosine models for the signal; these are illustrated in Figure S2. The best fit model achieves a correlation coefficient to the signal of 0.5; seven out of thirty-one starting parameters converged to this solution.

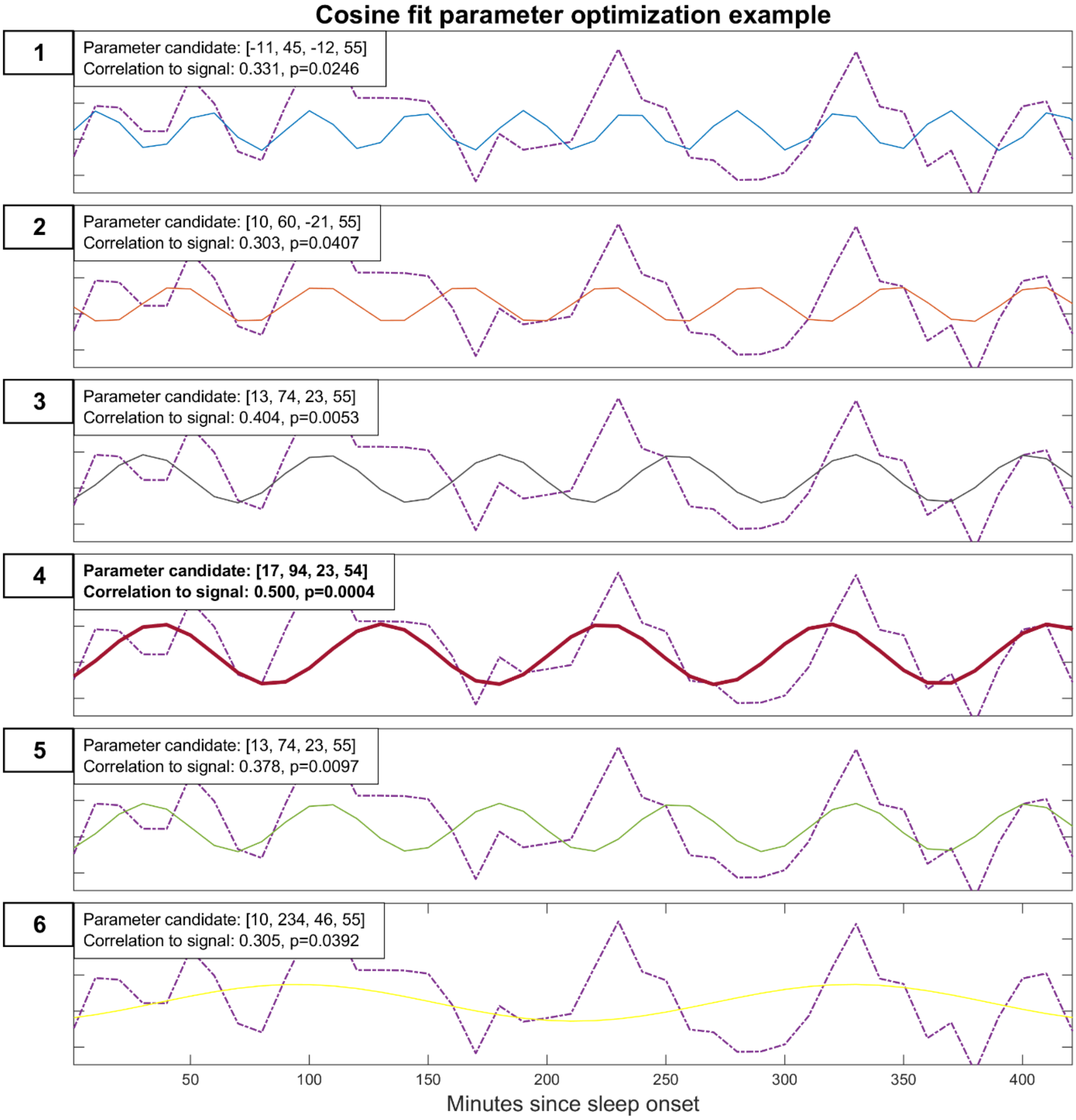

Figure S2: Thirty-one different starting parameters converge to six candidate solutions. Of these, the best fit is found in subplot 4, with a correlation coefficient of 0.5 and a period of 94 minutes.

Table S1: Optimization example, all results

One set of optimizations for one example signal converged to six candidate solutions. Identical (and functionally identical) results are color coded. The best fit (chosen by highest correlation between signal and model) is highlighted in red.

Starting period	Results				Fit statistics	
	Amplitude	Period	Phase	Offset	r	p
30	-11	45	-12	55	0.331	0.0246
35	-11	45	-12	55	0.331	0.0246
40	-11	45	-12	55	0.331	0.0246
45	10	60	-21	55	0.303	0.0407
50	10	60	-21	55	0.303	0.0407
55	10	60	-21	55	0.303	0.0407
60	-13	74	-14	55	0.404	0.0053
65	-13	74	-14	55	0.404	0.0053
**70**	**-17**	**94**	**-24**	**54**	**0.500**	**0.0004**
75	13	74	23	55	0.404	0.0053
80	13	74	23	55	0.404	0.0053
85	13	74	23	55	0.404	0.0053
90	-13	127	23	56	0.378	0.0097
95	-13	127	23	56	0.378	0.0097
**100**	**17**	**94**	**23**	**54**	**0.500**	**0.0004**
105	-13	127	23	56	0.378	0.0097
**110**	**17**	**94**	**23**	**54**	**0.500**	**0.0004**
**115**	**17**	**94**	**23**	**54**	**0.500**	**0.0004**
**120**	**17**	**94**	**23**	**54**	**0.500**	**0.0004**
**125**	**17**	**94**	**23**	**54**	**0.500**	**0.0004**
**130**	**17**	**94**	**23**	**54**	**0.500**	**0.0004**
135	-10	234	-70	55	0.305	0.0392
140	10	234	46	55	0.305	0.0392
145	-10	234	-70	55	0.305	0.0392
150	-10	234	-70	55	0.305	0.0392
155	-10	234	-70	55	0.305	0.0392
160	-10	234	-70	55	0.305	0.0392
165	13	127	86	56	0.378	0.0097
170	-10	234	-70	55	0.305	0.0392
175	-13	127	23	56	0.378	0.0097
180	13	127	86	56	0.378	0.0097

Code examples

The following code examples show three MATLAB-functions:

1. `get_LIDS` shows our implementation of the LIDS transformation
2. `cosine_fit` uses the `fminsearch` function to solve the optimization problem to find the best-fit cosine model. After performing thirty optimizations from the chosen starting parameters, we choose out of the candidate parameter sets the model with the highest correlation to the original signals. Several of the starting points will lead to the same result, i.e., they converge to the same local minimum. Identifying several local minima increases the odds of finding an actual optimal solution.
3. `fitAndResample` shows the implementation of the resampling and synchronization process, wherein after finding the best-fit cosine model, the timelines for each individual signal were converted from 'external' (real) time to an 'internal' times and then phase synchronized by the first peak in their optimal cosine curve.

```
function [LIDS] = get_LIDS(activity, rate, varargin)
%get_LIDS Calculates LIDS for activity data sampled at a given rate
% Input arguments:
%       activity            vector of activity data
%       rate                signal rate in Hertz
%       rescaling_factor    optional factor for rescaling the binned activity
(default = 1000)

p = inputParser;
addOptional(p,'rescaling_factor', 1000);
parse(p,varargin{:});
p = p.Results;
rescaling_factor = p.rescaling_factor;

% make 10-minute activity bins
windowlength = 10*60; %windowlength in seconds
ppb = windowlength*rate; %'points per bin'
actisum = activity(1:end-mod(length(activity),ppb));
actisum = sum(reshape(actisum, [ppb, length(actisum)/ppb]));
% add remainder into a final bin
if ~mod(length(activity),ppb)==0
    remainder = sum(activity(end-mod(length(activity),ppb)));
    actisum(end+1) = remainder;
end

% rescale the binned activity
actisum = rescale(actisum, 0, rescaling_factor);

% calculate LIDS
LIDS = 100./(actisum+1);
LIDS = movmean(LIDS, 3)';
end
```

```matlab
function [theta_bestfit, res, yhat] = cosine_fit(y, t)
%COSINE_FIT Searches for the best-fit cosine-function for signal y over
%time t. Unconstrained optimization with post-hoc reasonableness adjustments,
%starting periods in every 5-minute step between 30 and 150 minutes.
% Input arguments:
%       y             signal to fit
%       t             time (in 10-minute bins)
% Output arguments:
%       theta_bestfit: [ampltude, period, phase, offset] of best-fit cosine model
%       res:            fit statistics
%       yhat:           best-fit cosine model over time vector t
    [m, ~] = size(t); if m==1;t=t';end
    theta_all = zeros(0,4);
    results = zeros(0,4);
    fitfunction = @(b,t)  b(1).*(sin((2*pi/b(2))*(t - b(3)))) + b(4);
    costfunction = @(b) sum((fitfunction(b,t) - y).^2);
    for per = 3:0.5:18
        theta0 = [range(y); per; 0; mean(y)];
        A = []; b = []; Aeq = []; beq = []; nonlcon=[];
        options = optimset('MaxFunEvals', 300*length(theta0));
        theta = fminsearch(costfunction, theta0, options);
        theta_all(end+1,:) = theta;
        yhat = fitfunction(theta, t);
        [r, p] = corrcoef(y,yhat);
        r = r(2); p = p(2);
        RoO = range(yhat);
        MRI = RoO*r;
        results(end+1,:) = [r, RoO, MRI, p];
    end

    % choose the best period that is shorter than 180 and longer than 30 minutes
    optimization_criterion = 1; %correlation coefficient
    [~, idx] = max(results(:,optimization_criterion));
    bestPer = theta_all(idx,2);
    restemp = results; theta_all_temp = theta_all; index = 1:length(theta_all);
    while (18 < bestPer) || (bestPer < 3)
        restemp(idx,:) = []; theta_all_temp(idx,:) = []; index(idx)=[];
        [~, idx] = max(restemp(:,optimization_criterion));
        bestPer = theta_all_temp(idx,2);
    end
    idx = index(idx);
    if results(idx, 4)>0.05; fprintf('WARNING: Non-significant cosine fit!\n');  end
    theta_bestfit = theta_all(idx,:);
    res = results(idx,:);

    %shift to closest equivalent phase not larger than period
    while abs(theta_bestfit(3)) > theta_bestfit(2)
        if theta_bestfit(3) < 0; theta_bestfit(3) = theta_bestfit(3)+theta_bestfit(2);
        else; theta_bestfit(3) = theta_bestfit(3)-theta_bestfit(2); end
    end
    %if amplitude is negative, use absolute amplitude and add 1/2 period to the phase:
    if theta_bestfit(1) < 0
       theta_bestfit(1) = abs(theta_bestfit(1));
       if theta_bestfit(3) < 0; theta_bestfit(3) = theta_bestfit(3)+0.5*theta_bestfit(2);
       else; theta_bestfit(3) = theta_bestfit(3)-0.5*theta_bestfit(2); end
    end
    if theta_bestfit(3) < 0
        theta_bestfit(3) = theta_bestfit(3) + theta_bestfit(2);
    end
    yhat = fitfunction(theta_bestfit, t);
    fprintf('Cosine parameters: %.4f - %.4f - %.4f - %.4f\n',theta_bestfit);
end
```

```
function [y_synchronized, y_resampled, yhat, yhat_synchronized, yhat_resampled,
fitStats] = fitAndResample(y, moffset)
%FITANDRESAMPLE Calculates best-fit cosine model for signal y. Resamples to
%90 minute period 'internal time'. Synchronizes so that the first peak in
%the resampled signal occurs after moffset number of bins.
%
% Input arguments:
%       y            signal to fit
%       moffset      number of bins before first peak in synchronized
%                       output (after resampling)
% Output arguments:
%       y_synchronized: resampled y synchronized to have first peak at moffset
%       y_resampled: y resampled to 90 minute period 'internal time'
%       yhat: best-fit cosine model over time vector t
%       yhat_synchronized: resampled and synchronized yhat
%       yhat_resampled: resampled yhat
%       fitStats: [ampltude, period, phase, offset] of best-fit cosine
%                   model, and the fit statistics

    y(isnan(y))=[];

    [theta_bestfit, res, yhat] = cosine_fit(y, (1:length(y))');
    fitStats = [theta_bestfit, res];
    % calculate 'internal time' to normalize period to 90 minutes, resample y
    optPer = 10*theta_bestfit(2);

    % resample to new rate
    new_rate = 60*optPer/9;
    old_rate = 10*60;
    y_resampled = resample(y,old_rate,round(new_rate));
    yhat_resampled = resample(yhat, old_rate, round(new_rate));

    %synchronize by first LIDS peak
    [~,yhatpeaks_resampled] = findpeaks(yhat_resampled, 'MinPeakProminence',6);
    y_synchronized = y_resampled;
    yhat_synchronized = yhat_resampled;
    if yhatpeaks_resampled(1)-moffset >= 0
        y_synchronized(1:yhatpeaks_resampled(1)-moffset) = [];
        yhat_synchronized(1:yhatpeaks_resampled(1)-moffset) = [];
    else
        y_synchronized = [nan(moffset-yhatpeaks_resampled(1),1); y_synchronized];
        yhat_synchronized = [nan(moffset-yhatpeaks_resampled(1),1);
yhat_synchronized];
    end
end
```

Mixed model analysis results

The full results of the mixed model analyses can be found in Supplementary Table S1.

Complete figure sets

Attached to this supplement are two pdf-files, *Figure3.pdf* and *Figure6.pdf*, that contain complete sets of figures in styles corresponding to the respective figure in the main text of this work, generated for all data in the dataset.

Actigraphy sleep/wake state scored with past-10 future-10 model. Radar sleep/wake state scored with past-5 future-0 model. Real-time processing is not relevant for actigraphy since actigraphy data can't be accessed in real time.

Figure 3.pdf

A complete set of figures in the style of Figure 3 from the main text, including all nights from all participants wherein all three activity sensors (Actiwatch Spectrum, Radar (ceiling), and Radar (nightstand)) and PSG was recorded; each figure contains data from one night.

The PSG hypnogram is inverted and averaged into 10-minute bins, then rescaled to a range of [0, 100], to correspond with the temporal resolution and numerical range of the LIDS transform. This PSG-INH is plotted together with the three sensor-derived LIDS signals in the final subplot.

LIDS, Locomotor Inactivity During Sleep; PSG-INH, Polysomnography – Inverted Numerical Hypnogram

Figure 6.pdf

Contains three temporal raster plots for each participant, generated with data from actigraphy, ceiling radar, and nightstand radar respectively. Each plot shows all nights of recording for the participant, with activity data from the given sensor plotted along with LIDS derived from that sensor. Background colours indicate state and PSG information is included where available.

LIDS, Locomotor Inactivity During Sleep; PSG-INH, Polysomnography – Inverted Numerical Hypnogram

References

Winnebeck, E. C., Fischer, D., Leise, T., & Roenneberg, T. (2018). Dynamics and Ultradian Structure of Human Sleep in Real Life. *Current Biology, 28*(1), 49-59. doi:10.1016/j.cub.2017.11.063

Table S2. Mixed model analysis of LIDS and PSG-INH over normalized timelines

Target variables were LIDS and PSG-INH over normalized timelines, with PSG-INH as the reference. 'cycle' refers to LIDS (or PSG-INH) cycle. Data were analysed in bins of normalized duration (internal time = 90 minutes), starting from PSG sleep onset and ending at the prescribed rise time for the participants.

Model information:

Number of observations	7164
Fixed effects coefficients	12
Random effects coefficients	306
Covariance parameters	5

Formula

parval ~ 1 + cycle*device + sex*device + (1 | PID) + (1 | PID:nightnum) + (1 | PID:nightnum:cycle) + (cycle | PID)

Model fit statistics:

AIC	BIC	LogLikelihood	Deviance
65576	65693	-32771	65542

Fixed effects coefficients (95% CIs):

Name	Estimate	SE	tStat	DF	pValue	Lower	Upper
(Intercept)	86.49	1.72	50.39	7152	0	83.12	89.85
cycle	-6.40	0.47	-13.63	7152	8.97E-42	-7.32	-5.48
sex_m	-4.36	1.61	-2.71	7152	6.70E-03	-7.51	-1.21
device_Radar (nightstand)	-14.51	1.89	-7.66	7152	2.16E-14	-18.22	-10.79
device_Radar (ceiling)	-20.24	1.96	-10.32	7152	8.69E-25	-24.09	-16.40
device_Actiwatch Spectrum	-18.87	1.89	-10.01	7152	2.06E-23	-22.57	-15.17
cycle:device_Radar (nightstand)	1.31	0.57	2.27	7152	2.31E-02	0.18	2.43
cycle:device_Radar (ceiling)	0.91	0.59	1.54	7152	1.23E-01	-0.25	2.07
cycle:device_Actiwatch Spectrum	2.00	0.57	3.48	7152	5.10E-04	0.87	3.12
sex_m:device_Radar (nightstand)	-4.76	1.56	-3.06	7152	2.22E-03	-7.81	-1.71
sex_m:device_Radar (ceiling)	-0.59	1.59	-0.37	7152	7.10E-01	-3.71	2.53
sex_m:device_Actiwatch Spectrum	-6.07	1.55	-3.92	7152	8.90E-05	-9.11	-3.04

Random effects covariance parameters (95% CIs):

	Name1	Name2	Type	Estimate	Lower	Upper
Group: PID (12 Levels)	(Intercept)	(Intercept)	std	3.84e-08	NaN	NaN
Group: PID:nightnum (47 Levels)	(Intercept)	(Intercept)	std	1.60	0.40	5.67
Group: PID:nightnum:cycle (323 Levels)	(Intercept)	(Intercept)	std	5.78	4.93	6.77
Group: PID (12 Levels)	cycle	cycle	std	1.39	1.18	1.63
Group: Error	Res Std			23.04	22.66	23.43
