# Supplementary material for: Contact‐free radar recordings of body movement can reflect ultradian dynamics of sleep
Source: J Sleep Res. 2022 Jul 6;31(6):e13687. doi: 10.1111/jsr.13687 (PMC9786343; doi:10.1111/jsr.13687)
Supplement: Supplementary file 1 — Appendix S1 Supporting Information [file JSR-31-e13687-s001.zip › JSR_13687_Supporting Figures_Figure6.pdf]

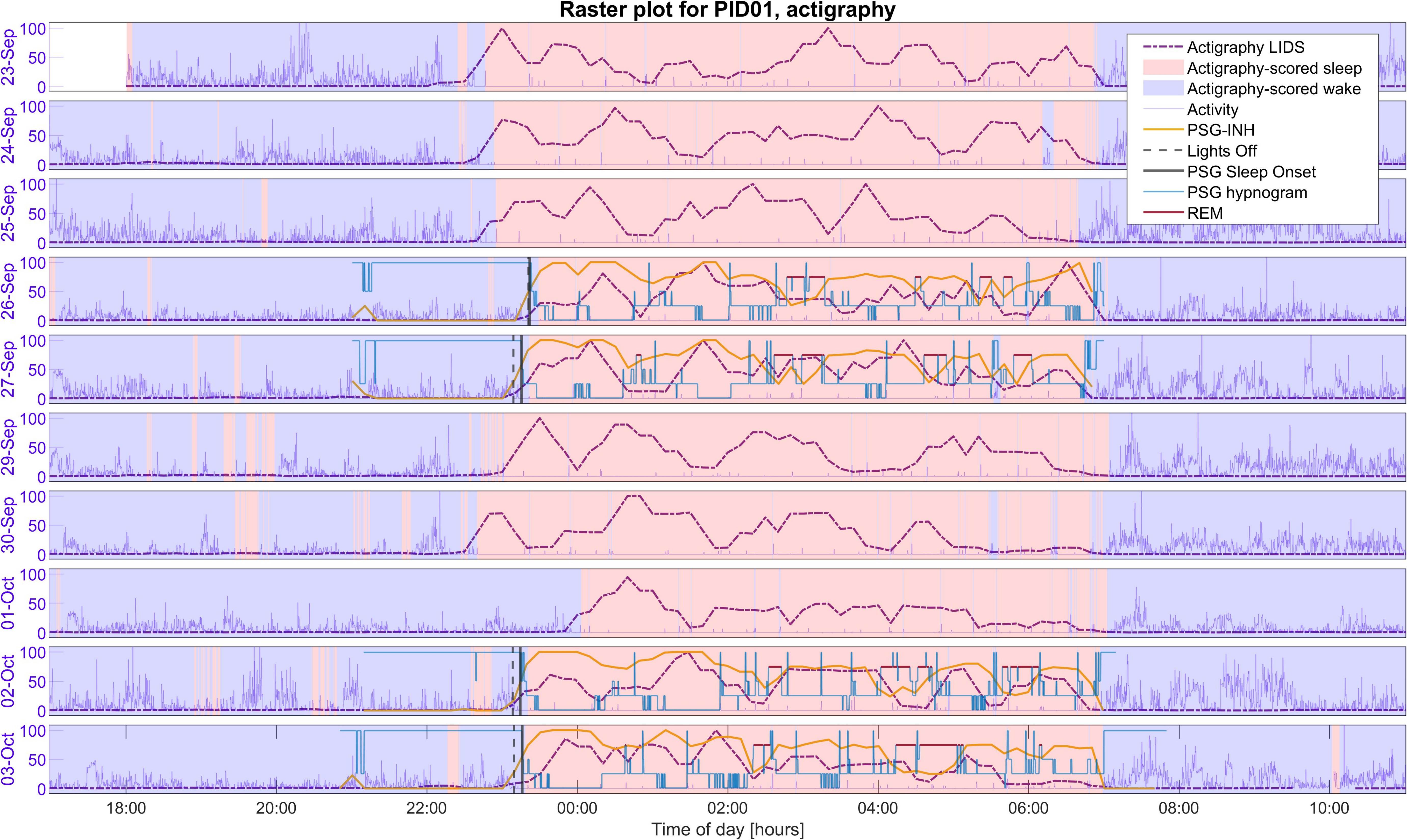
Raster plot for PID01, actigraphy
Actigraphy LIDS
Actigraphy-scored sleep
Actigraphy-scored wake
Activity
PSG-INH
Lights Off
PSG Sleep Onset
PSG hypnogram
REM
23-Sep
24-Sep
25-Sep
26-Sep
27-Sep
29-Sep
30-Sep
01-Oct
02-Oct
03-Oct
0
50
100
18:00
20:00
22:00
00:00
02:00
04:00
06:00
08:00
10:00
Time of day [hours]

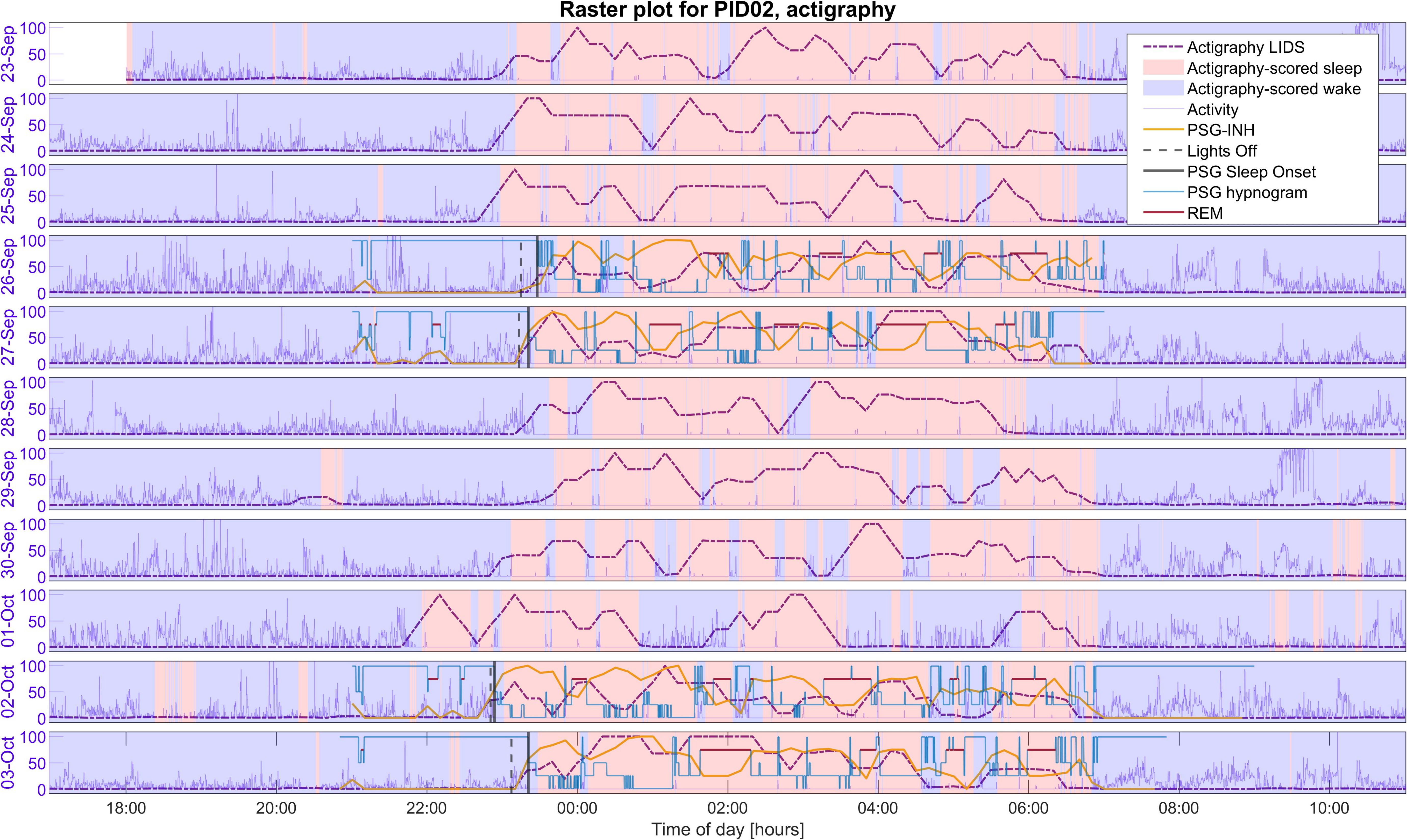

Raster plot for PID02, actigraphy
23-Sep
24-Sep
25-Sep
26-Sep
27-Sep
28-Sep
29-Sep
30-Sep
01-Oct
02-Oct
03-Oct
Actigraphy LIDS
Actigraphy-scored sleep
Actigraphy-scored wake
Activity
PSG-INH
Lights Off
PSG Sleep Onset
PSG hypnogram
REM
18:00
20:00
22:00
00:00
02:00
04:00
06:00
08:00
10:00
Time of day [hours]

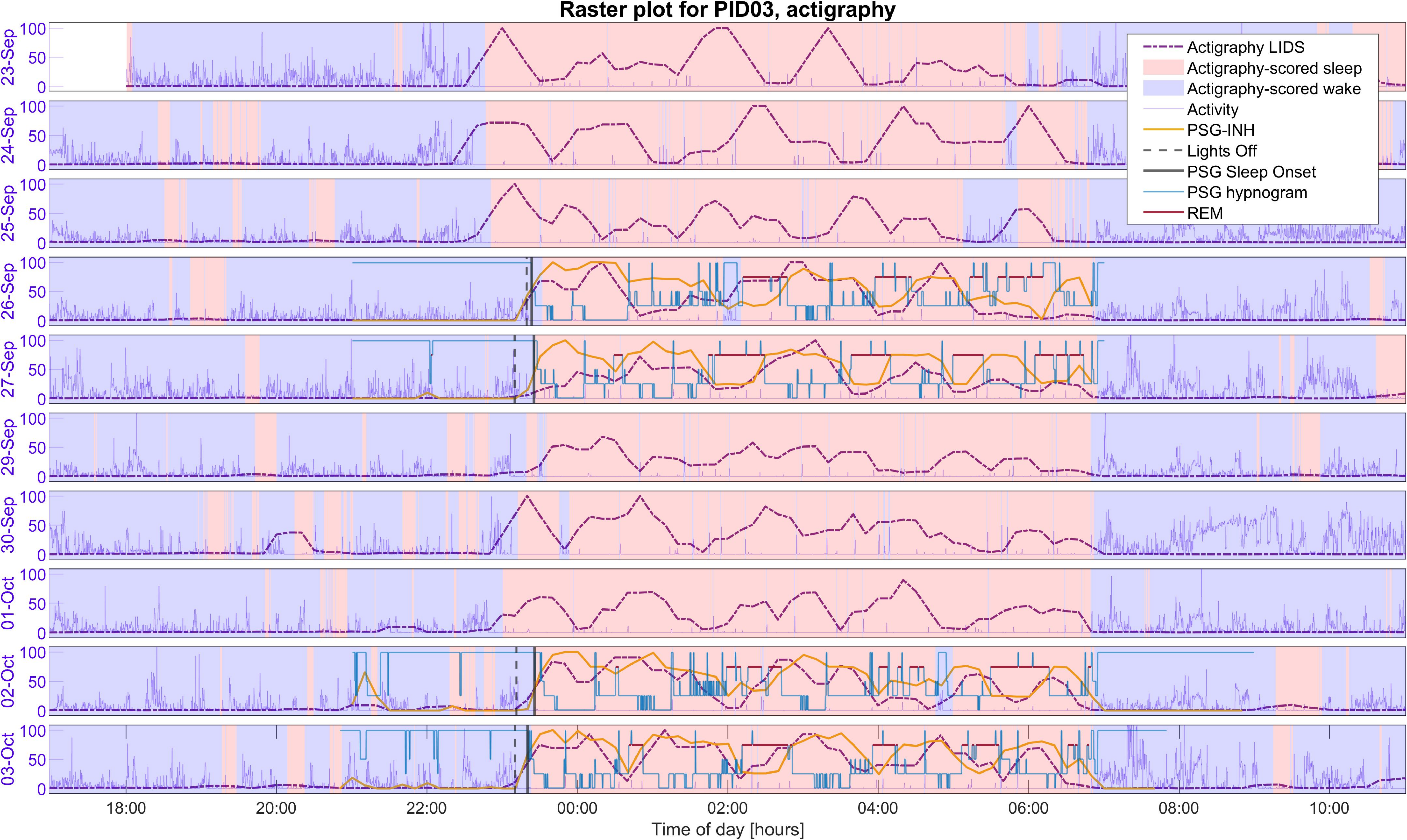
Raster plot for PID03, actigraphy
Actigraphy LIDS
Actigraphy-scored sleep
Actigraphy-scored wake
Activity
PSG-INH
Lights Off
PSG Sleep Onset
PSG hypnogram
REM
23-Sep
24-Sep
25-Sep
26-Sep
27-Sep
29-Sep
30-Sep
01-Oct
02-Oct
03-Oct
0
50
100
18:00
20:00
22:00
00:00
02:00
04:00
06:00
08:00
10:00
Time of day [hours]

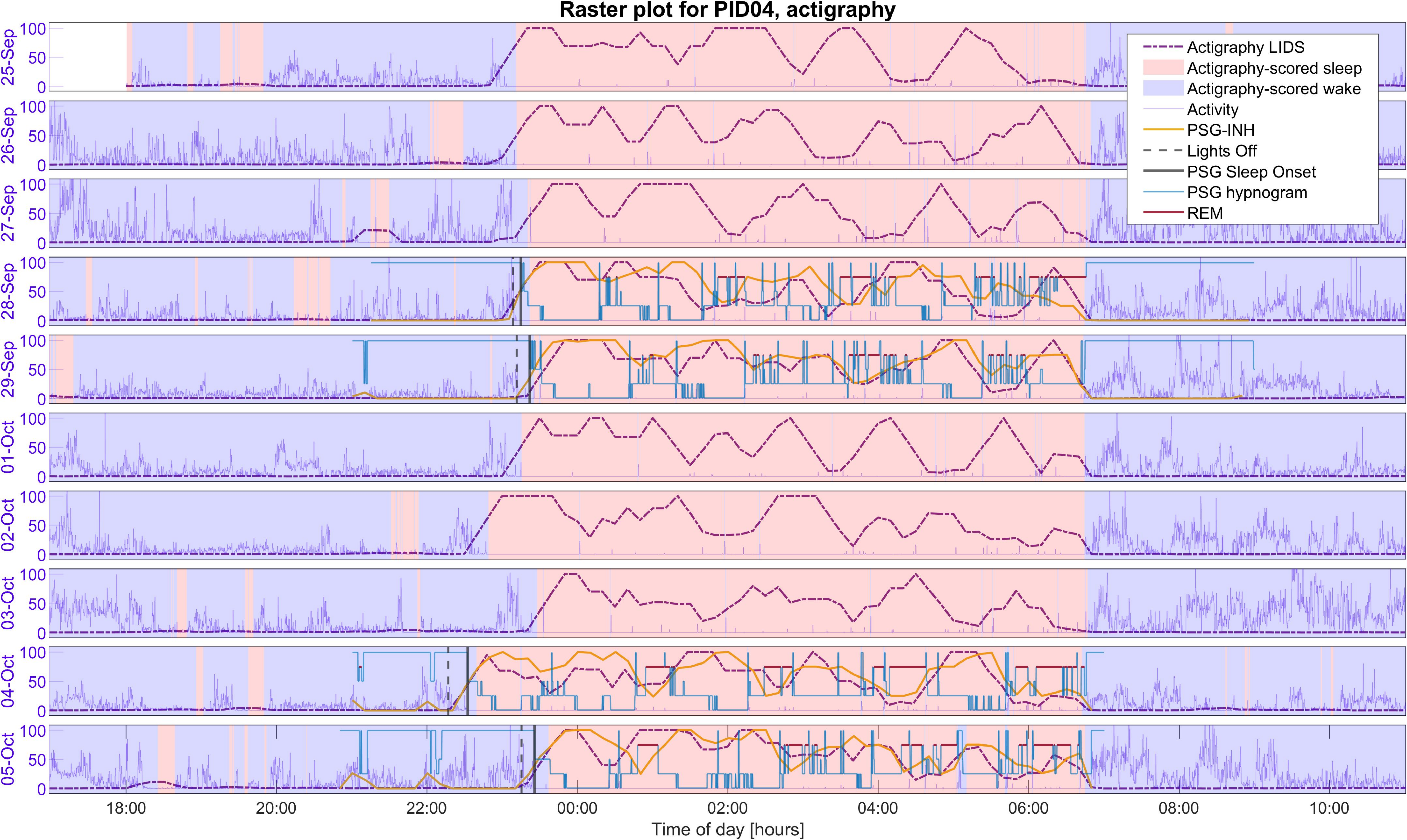

Raster plot for PID04, actigraphy
Actigraphy LIDS
Actigraphy-scored sleep
Actigraphy-scored wake
Activity
PSG-INH
Lights Off
PSG Sleep Onset
PSG hypnogram
REM
25-Sep
26-Sep
27-Sep
28-Sep
29-Sep
01-Oct
02-Oct
03-Oct
04-Oct
05-Oct
0
50
100
18:00
20:00
22:00
00:00
02:00
04:00
06:00
08:00
10:00
Time of day [hours]

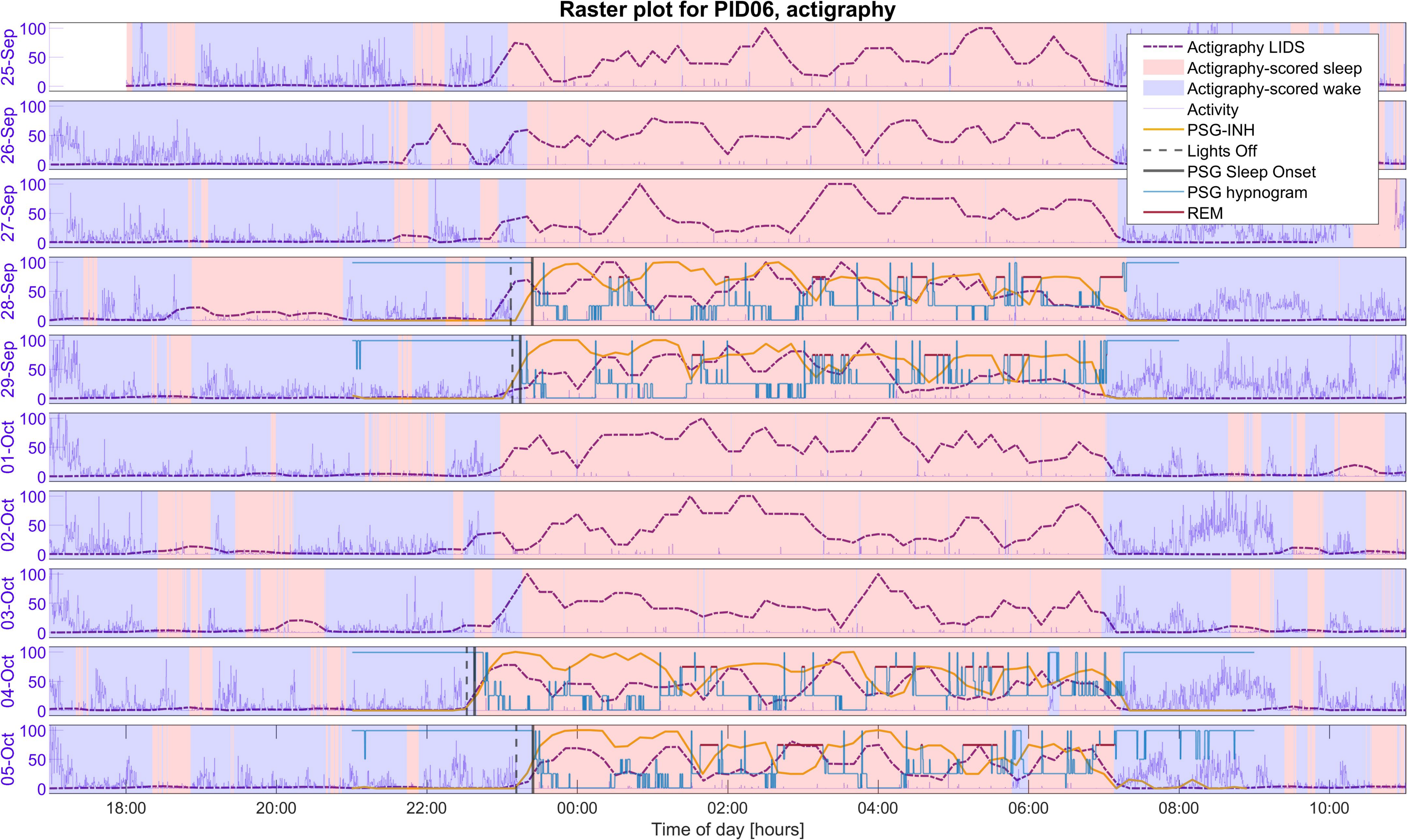
Raster plot for PID06, actigraphy
25-Sep
26-Sep
27-Sep
28-Sep
29-Sep
01-Oct
02-Oct
03-Oct
04-Oct
05-Oct
0
50
100
Actigraphy LIDS
Actigraphy-scored sleep
Actigraphy-scored wake
Activity
PSG-INH
Lights Off
PSG Sleep Onset
PSG hypnogram
REM
18:00
20:00
22:00
00:00
02:00
04:00
06:00
08:00
10:00
Time of day [hours]

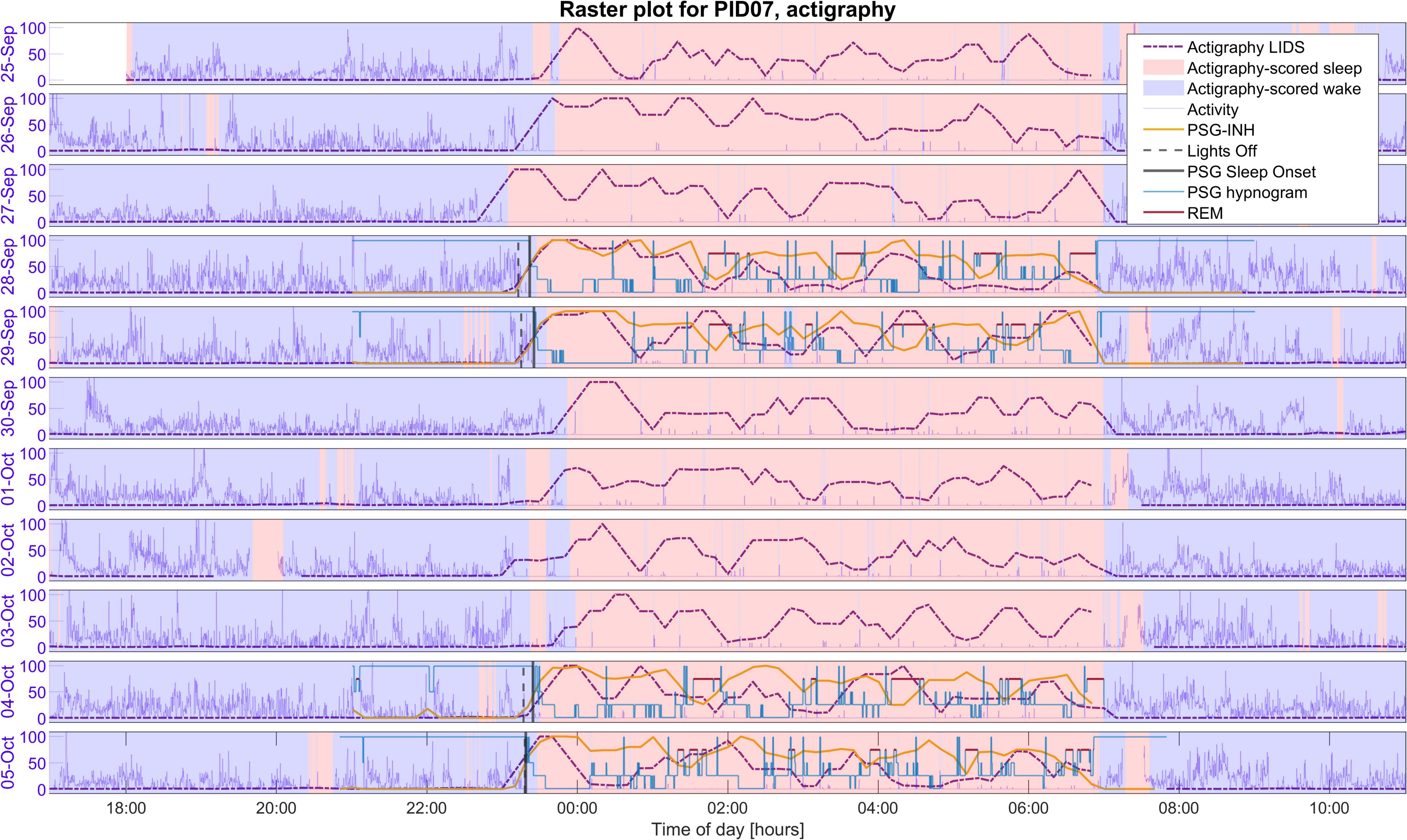

Raster plot for PID07, actigraphy
Actigraphy LIDS
Actigraphy-scored sleep
Actigraphy-scored wake
Activity
PSG-INH
Lights Off
PSG Sleep Onset
PSG hypnogram
REM
25-Sep
26-Sep
27-Sep
28-Sep
29-Sep
30-Sep
01-Oct
02-Oct
03-Oct
04-Oct
05-Oct
0
50
100
18:00
20:00
22:00
00:00
02:00
04:00
06:00
08:00
10:00
Time of day [hours]

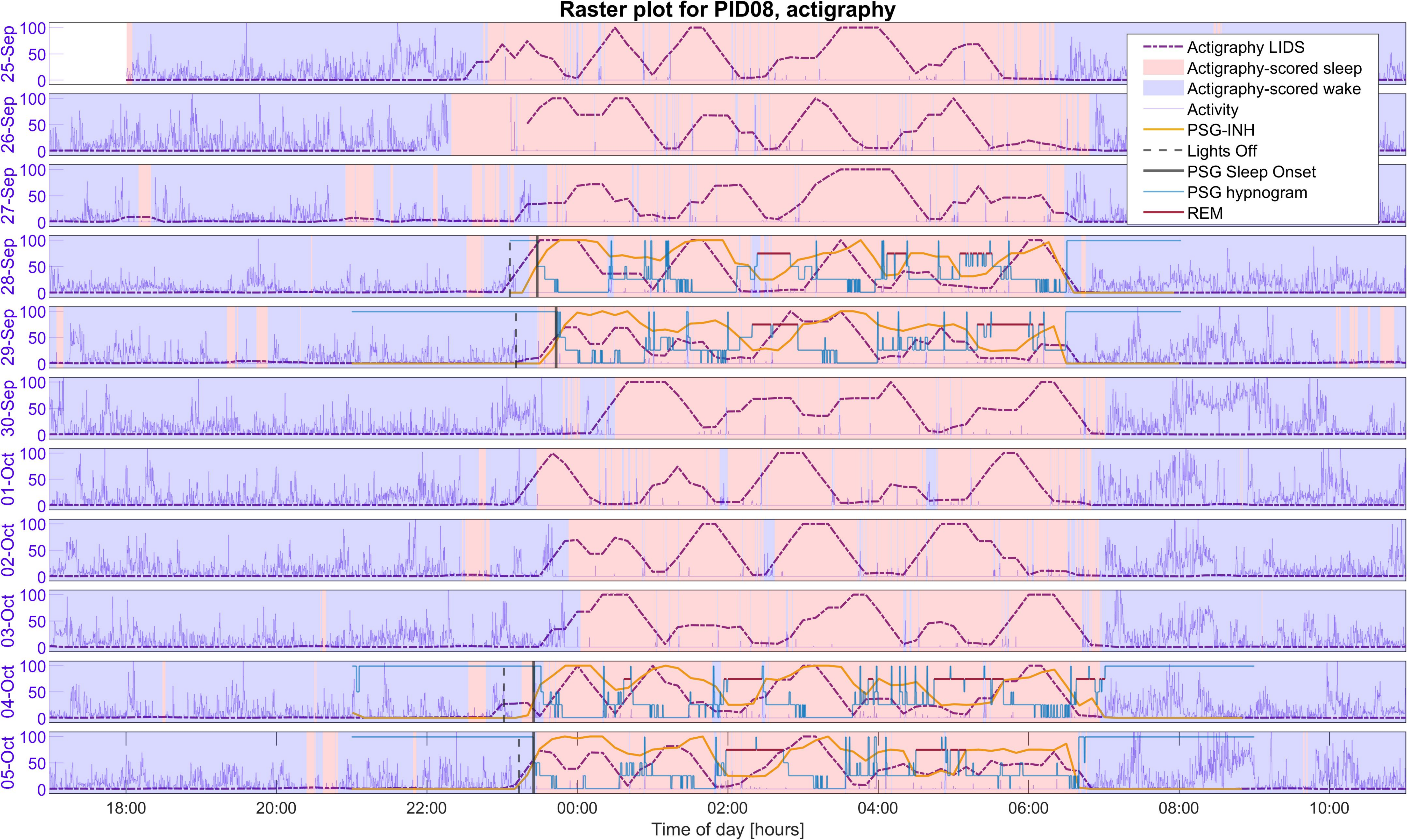

Raster plot for PID08, actigraphy
25-Sep
26-Sep
27-Sep
28-Sep
29-Sep
30-Sep
01-Oct
02-Oct
03-Oct
04-Oct
05-Oct
Actigraphy LIDS
Actigraphy-scored sleep
Actigraphy-scored wake
Activity
PSG-INH
Lights Off
PSG Sleep Onset
PSG hypnogram
REM
18:00
20:00
22:00
00:00
02:00
04:00
06:00
08:00
10:00
Time of day [hours]

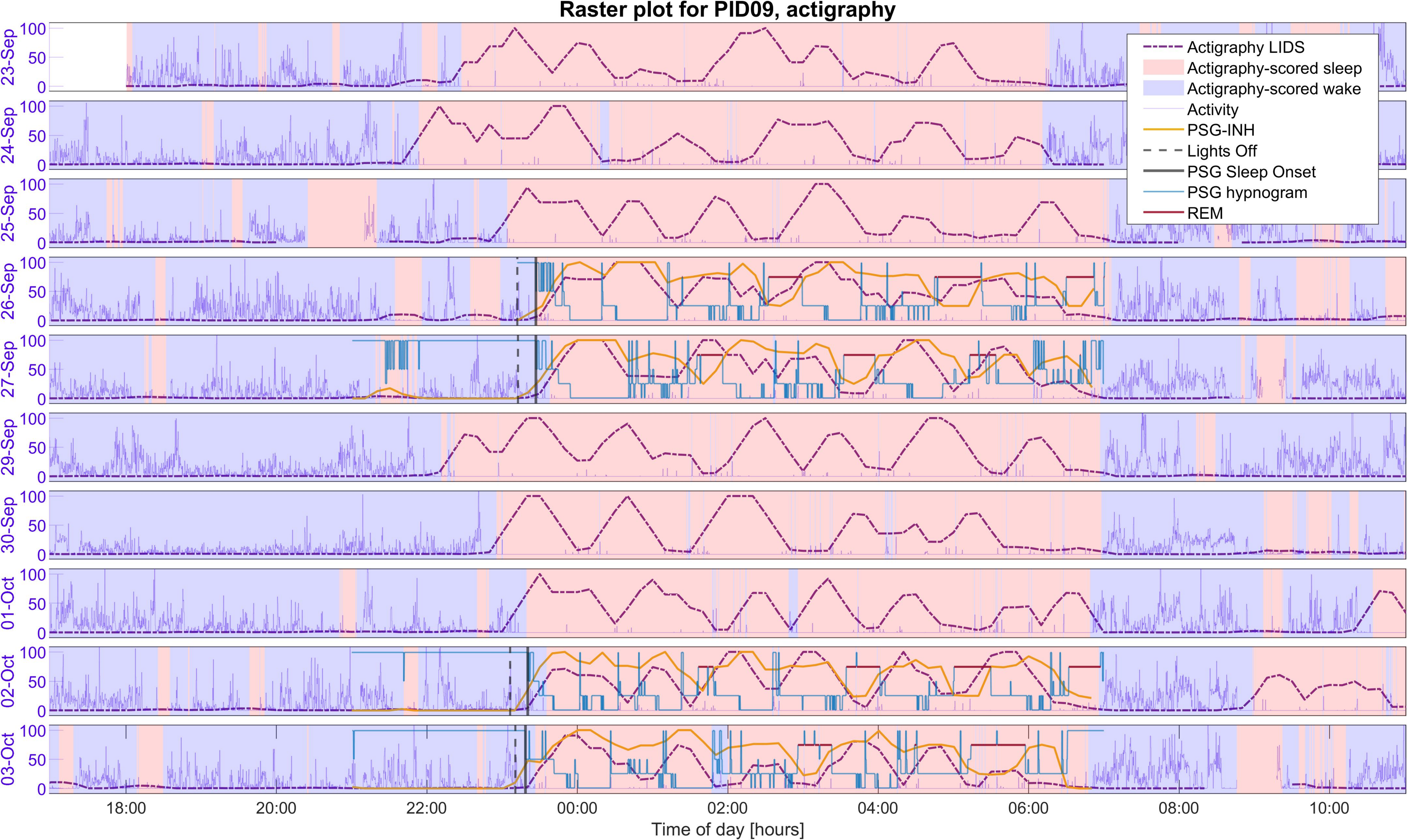

Raster plot for PID09, actigraphy
23-Sep
24-Sep
25-Sep
26-Sep
27-Sep
29-Sep
30-Sep
01-Oct
02-Oct
03-Oct
0
50
100
18:00
20:00
22:00
00:00
02:00
04:00
06:00
08:00
10:00
Time of day [hours]
Actigraphy LIDS
Actigraphy-scored sleep
Actigraphy-scored wake
Activity
PSG-INH
Lights Off
PSG Sleep Onset
PSG hypnogram
REM

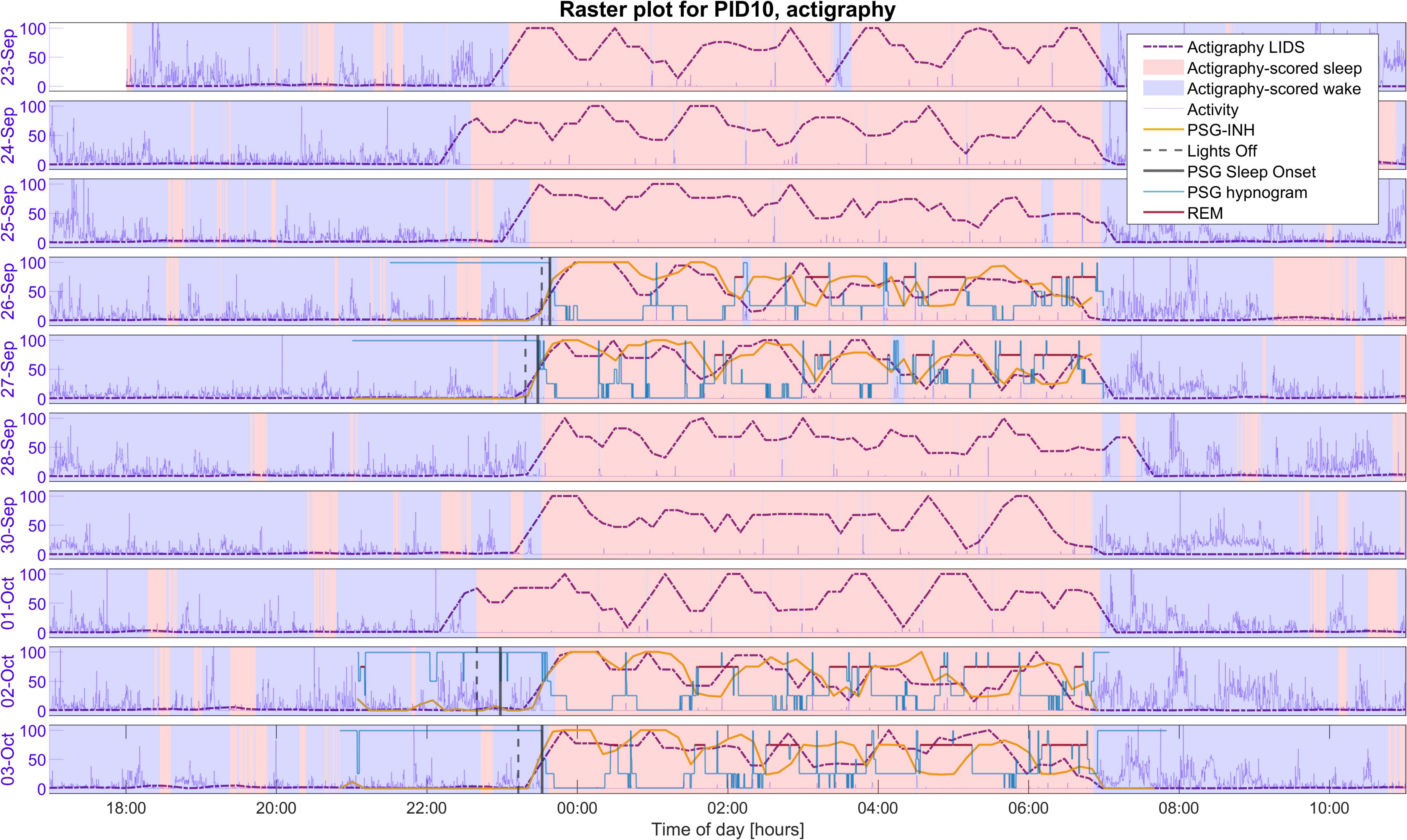

Raster plot for PID10, actigraphy
Actigraphy LIDS
Actigraphy-scored sleep
Actigraphy-scored wake
Activity
PSG-INH
Lights Off
PSG Sleep Onset
PSG hypnogram
REM
23-Sep
24-Sep
25-Sep
26-Sep
27-Sep
28-Sep
30-Sep
01-Oct
02-Oct
03-Oct
0
50
100
18:00
20:00
22:00
00:00
02:00
04:00
06:00
08:00
10:00
Time of day [hours]

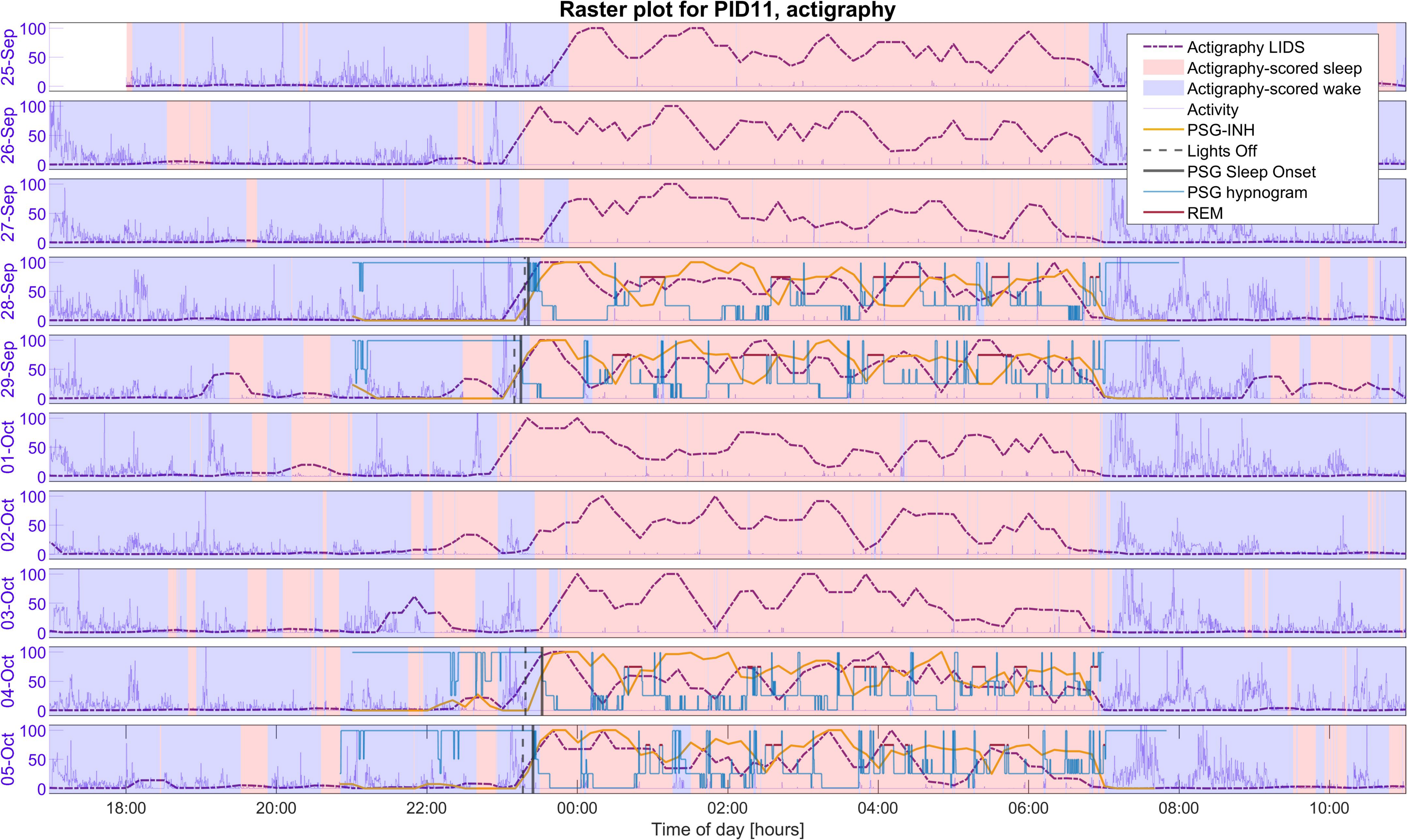

Raster plot for PID11, actigraphy
25-Sep
26-Sep
27-Sep
28-Sep
29-Sep
01-Oct
02-Oct
03-Oct
04-Oct
05-Oct
0
50
100
Actigraphy LIDS
Actigraphy-scored sleep
Actigraphy-scored wake
Activity
PSG-INH
Lights Off
PSG Sleep Onset
PSG hypnogram
REM
18:00
20:00
22:00
00:00
02:00
04:00
06:00
08:00
10:00
Time of day [hours]

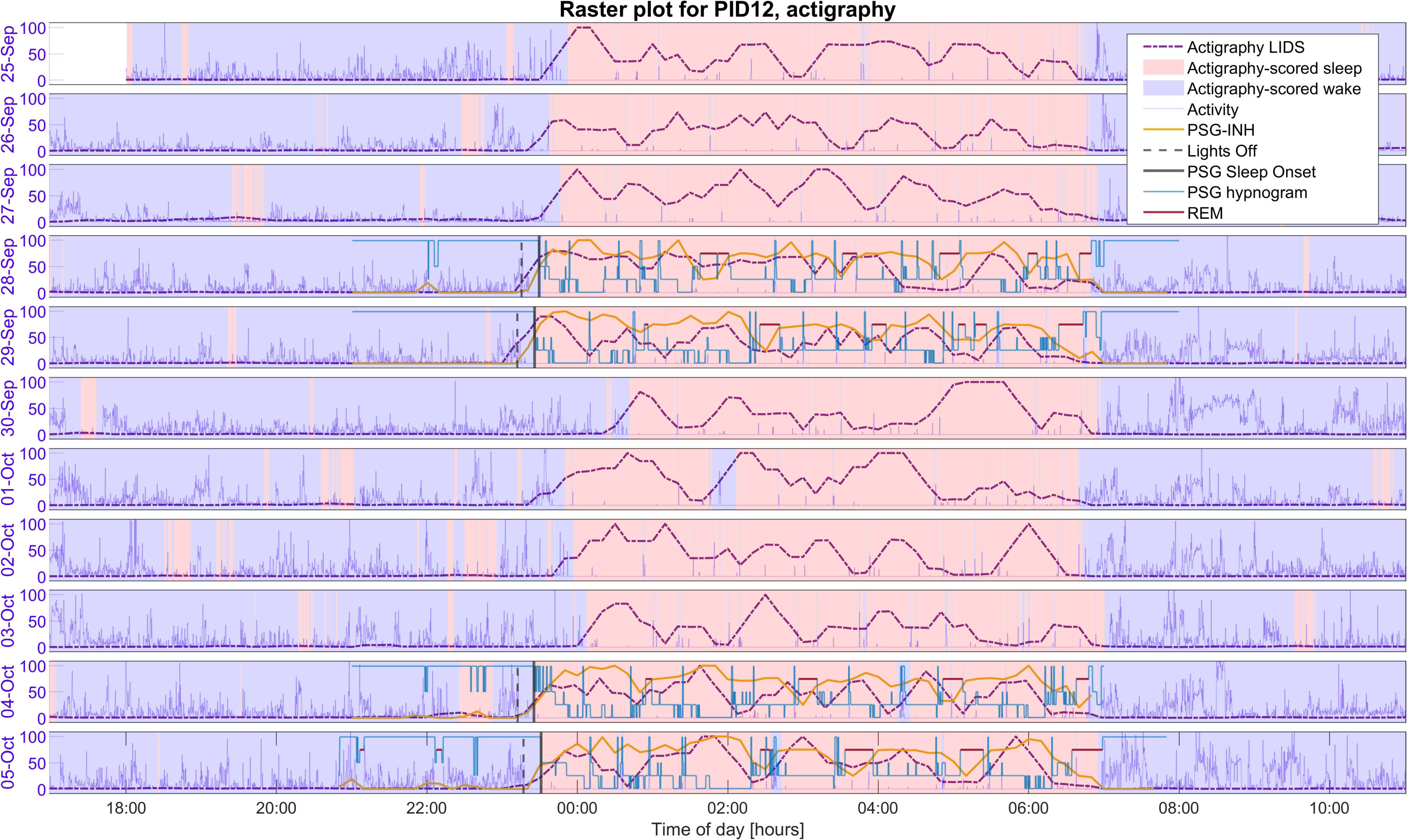

Raster plot for PID12, actigraphy
Actigraphy LIDS
Actigraphy-scored sleep
Actigraphy-scored wake
Activity
PSG-INH
Lights Off
PSG Sleep Onset
PSG hypnogram
REM
25-Sep
26-Sep
27-Sep
28-Sep
29-Sep
30-Sep
01-Oct
02-Oct
03-Oct
04-Oct
05-Oct
0
50
100
18:00
20:00
22:00
00:00
02:00
04:00
06:00
08:00
10:00
Time of day [hours]

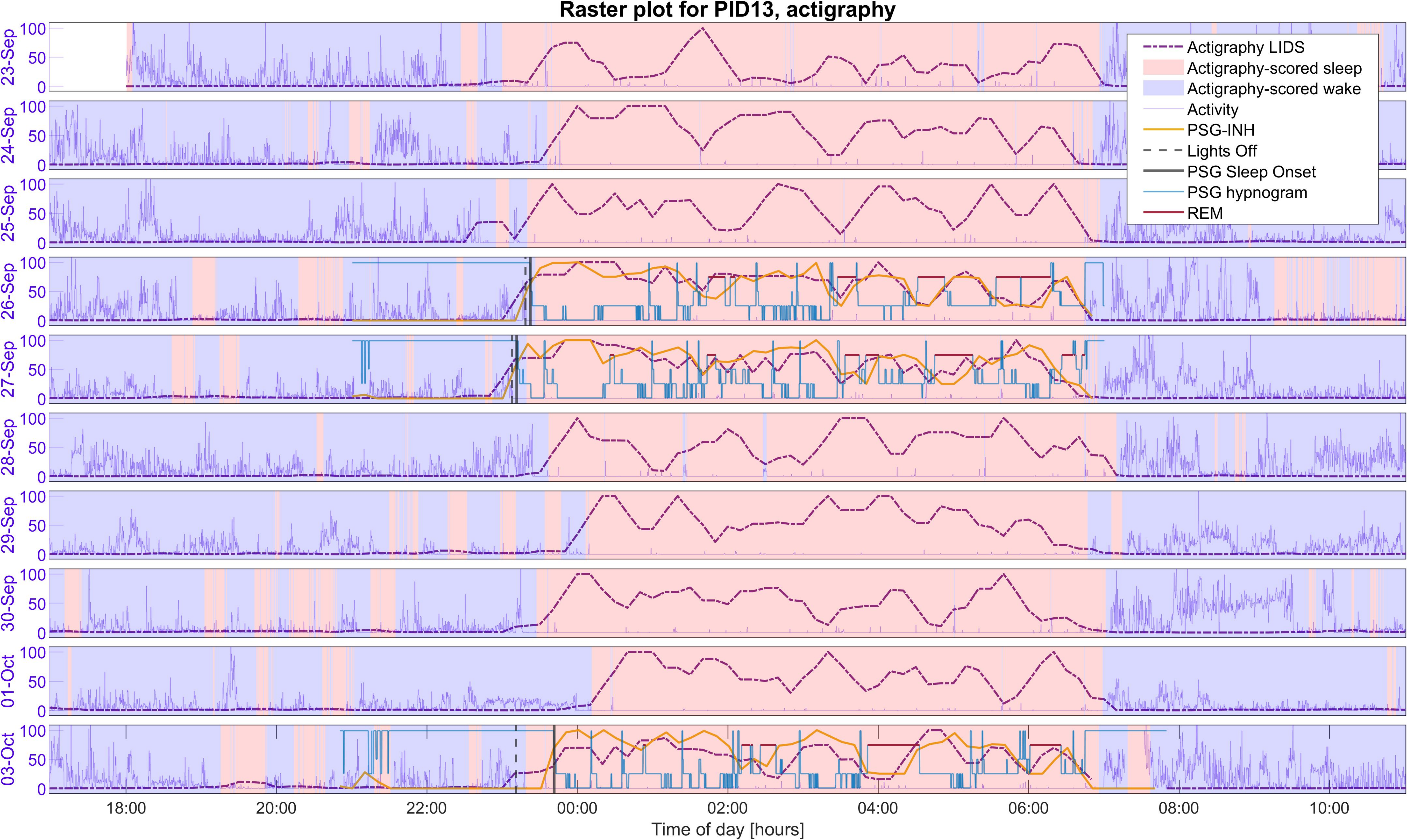

Raster plot for PID13, actigraphy
23-Sep
24-Sep
25-Sep
26-Sep
27-Sep
28-Sep
29-Sep
30-Sep
01-Oct
03-Oct
0
50
100
Actigraphy LIDS
Actigraphy-scored sleep
Actigraphy-scored wake
Activity
PSG-INH
Lights Off
PSG Sleep Onset
PSG hypnogram
REM
18:00
20:00
22:00
00:00
02:00
04:00
06:00
08:00
10:00
Time of day [hours]

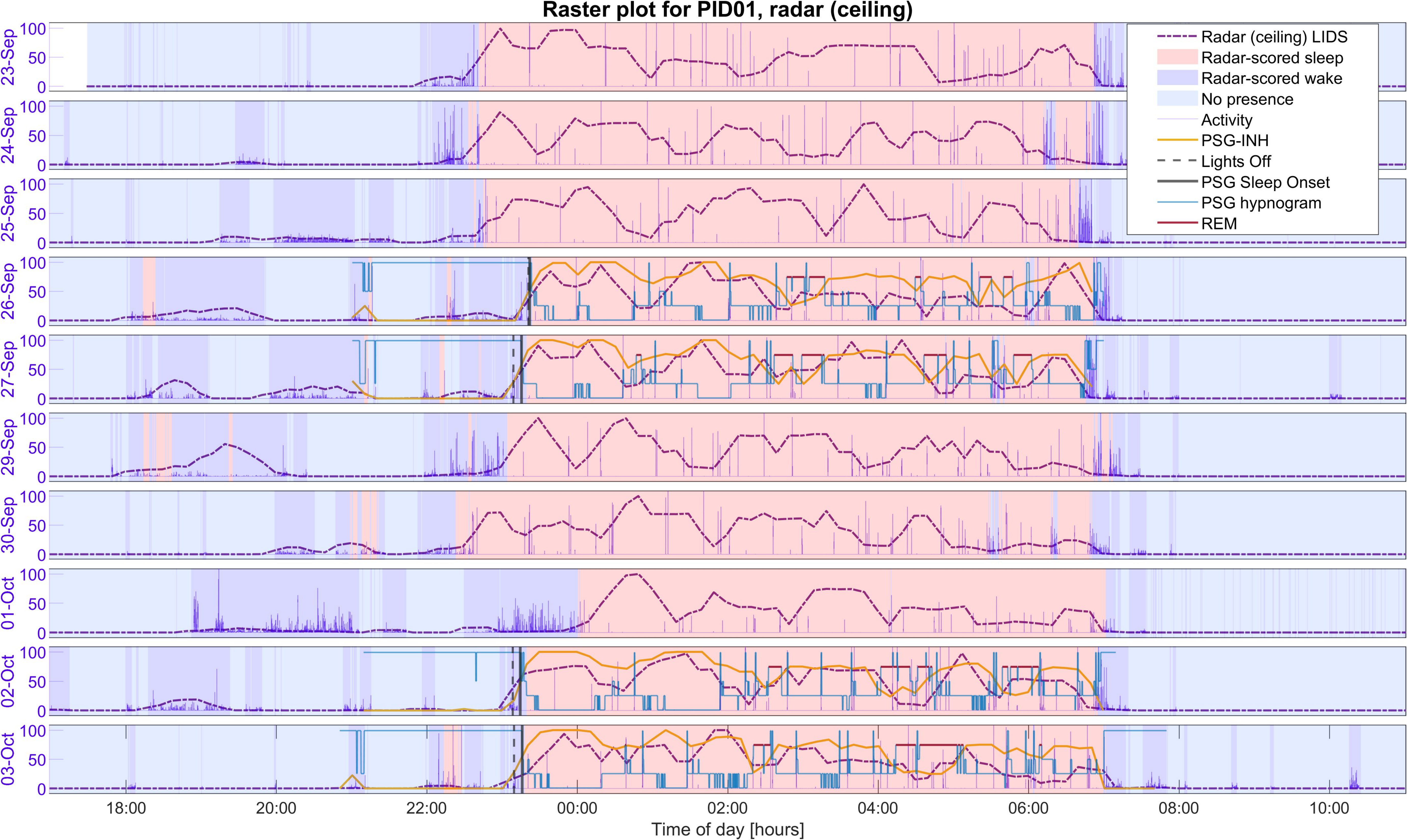

Raster plot for PID01, radar (ceiling)
Radar (ceiling) LIDS
Radar-scored sleep
Radar-scored wake
No presence
Activity
PSG-INH
Lights Off
PSG Sleep Onset
PSG hypnogram
REM
23-Sep
24-Sep
25-Sep
26-Sep
27-Sep
29-Sep
30-Sep
01-Oct
02-Oct
03-Oct
0
50
100
18:00
20:00
22:00
00:00
02:00
04:00
06:00
08:00
10:00
Time of day [hours]

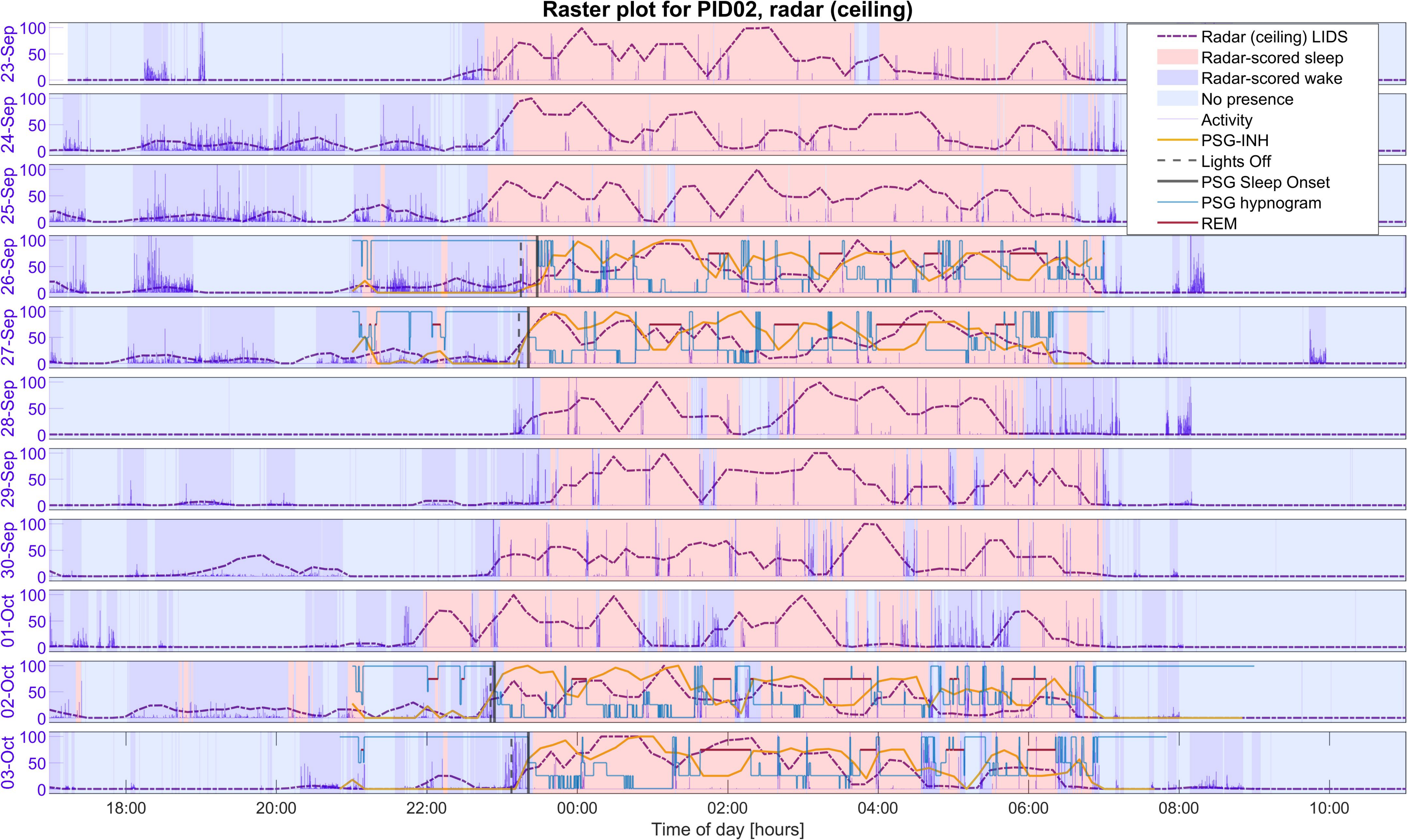
Raster plot for PID02, radar (ceiling)
23-Sep
24-Sep
25-Sep
26-Sep
27-Sep
28-Sep
29-Sep
30-Sep
01-Oct
02-Oct
03-Oct
Radar (ceiling) LIDS
Radar-scored sleep
Radar-scored wake
No presence
Activity
PSG-INH
Lights Off
PSG Sleep Onset
PSG hypnogram
REM
18:00
20:00
22:00
00:00
02:00
04:00
06:00
08:00
10:00
Time of day [hours]

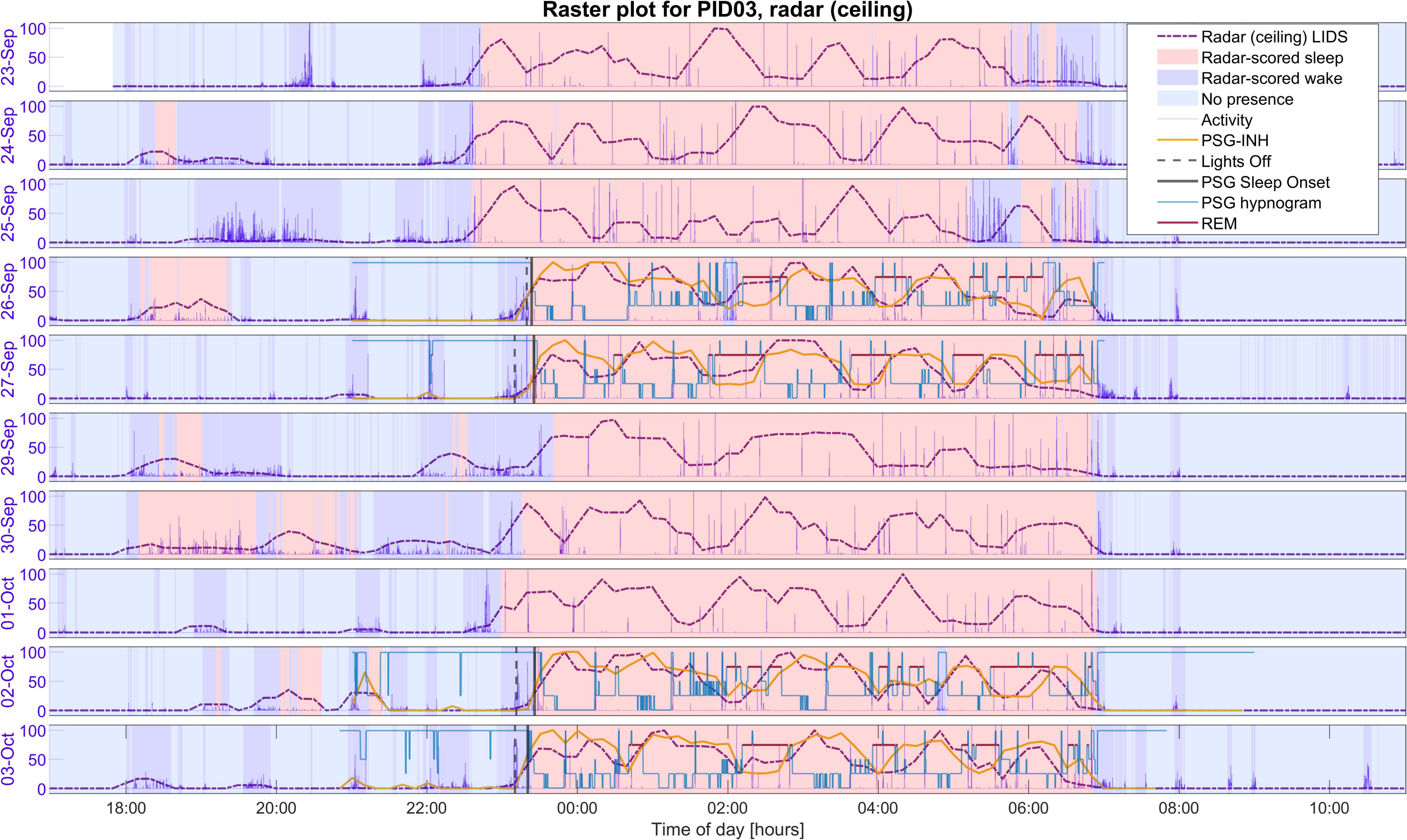

Raster plot for PID03, radar (ceiling)
Radar (ceiling) LIDS
Radar-scored sleep
Radar-scored wake
No presence
Activity
PSG-INH
Lights Off
PSG Sleep Onset
PSG hypnogram
REM
23-Sep
24-Sep
25-Sep
26-Sep
27-Sep
29-Sep
30-Sep
01-Oct
02-Oct
03-Oct
0
50
100
18:00
20:00
22:00
00:00
02:00
04:00
06:00
08:00
10:00
Time of day [hours]

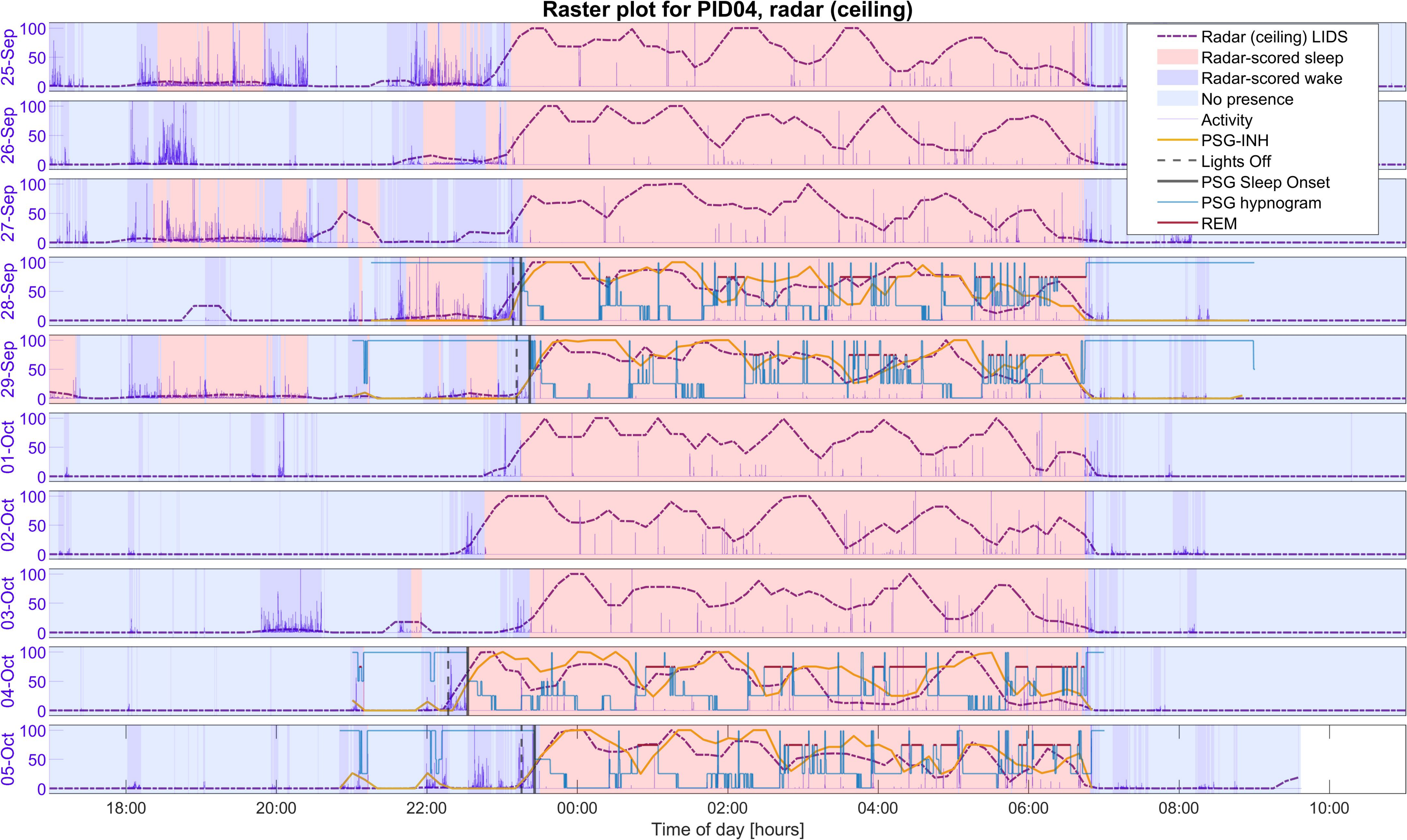

Raster plot for PID04, radar (ceiling)
25-Sep
26-Sep
27-Sep
28-Sep
29-Sep
01-Oct
02-Oct
03-Oct
04-Oct
05-Oct
0
50
100
Radar (ceiling) LIDS
Radar-scored sleep
Radar-scored wake
No presence
Activity
PSG-INH
Lights Off
PSG Sleep Onset
PSG hypnogram
REM
18:00
20:00
22:00
00:00
02:00
04:00
06:00
08:00
10:00
Time of day [hours]

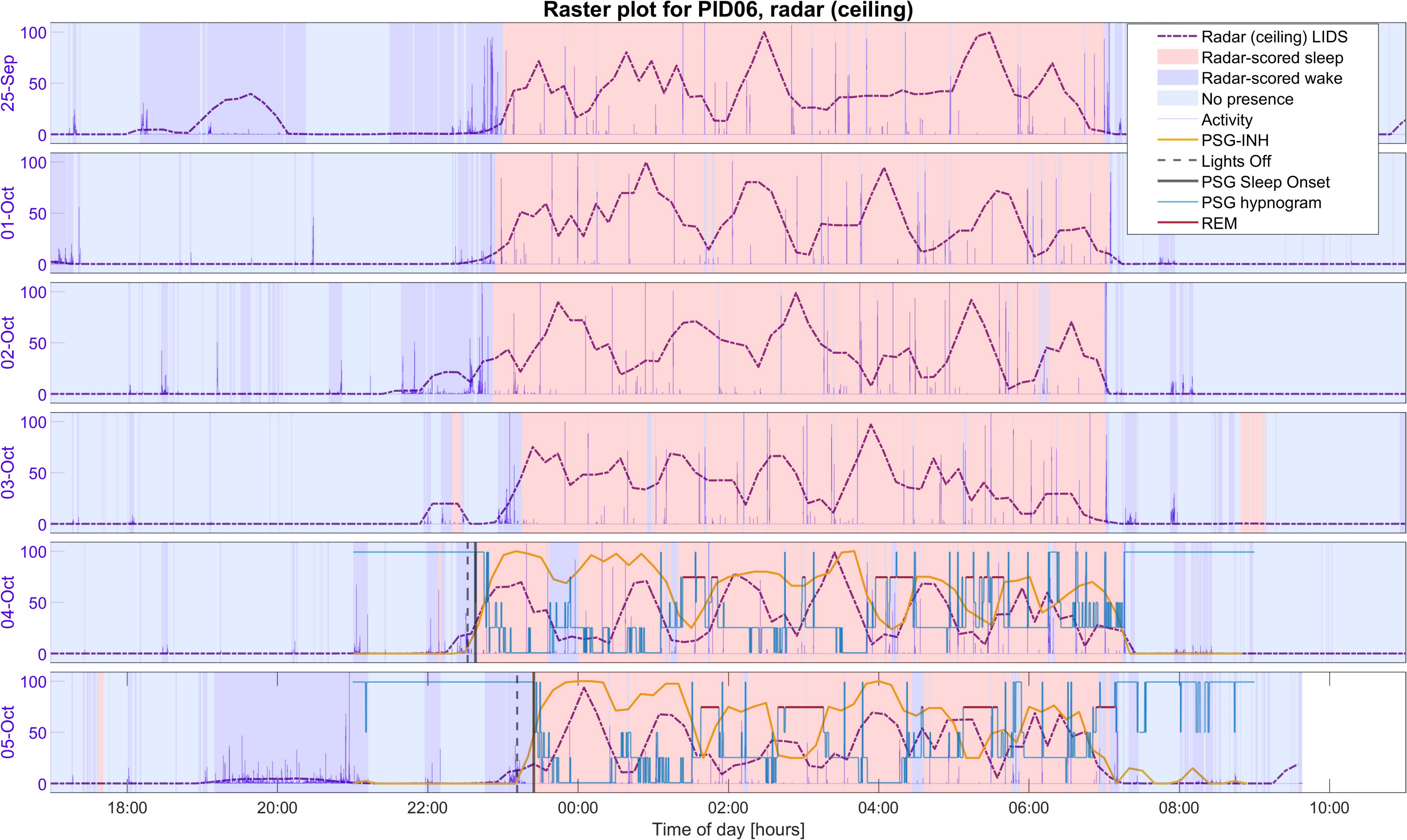

Raster plot for PID06, radar (ceiling)
Radar (ceiling) LIDS
Radar-scored sleep
Radar-scored wake
No presence
Activity
PSG-INH
Lights Off
PSG Sleep Onset
PSG hypnogram
REM
25-Sep
01-Oct
02-Oct
03-Oct
04-Oct
05-Oct
0
50
100
18:00
20:00
22:00
00:00
02:00
04:00
06:00
08:00
10:00
Time of day [hours]

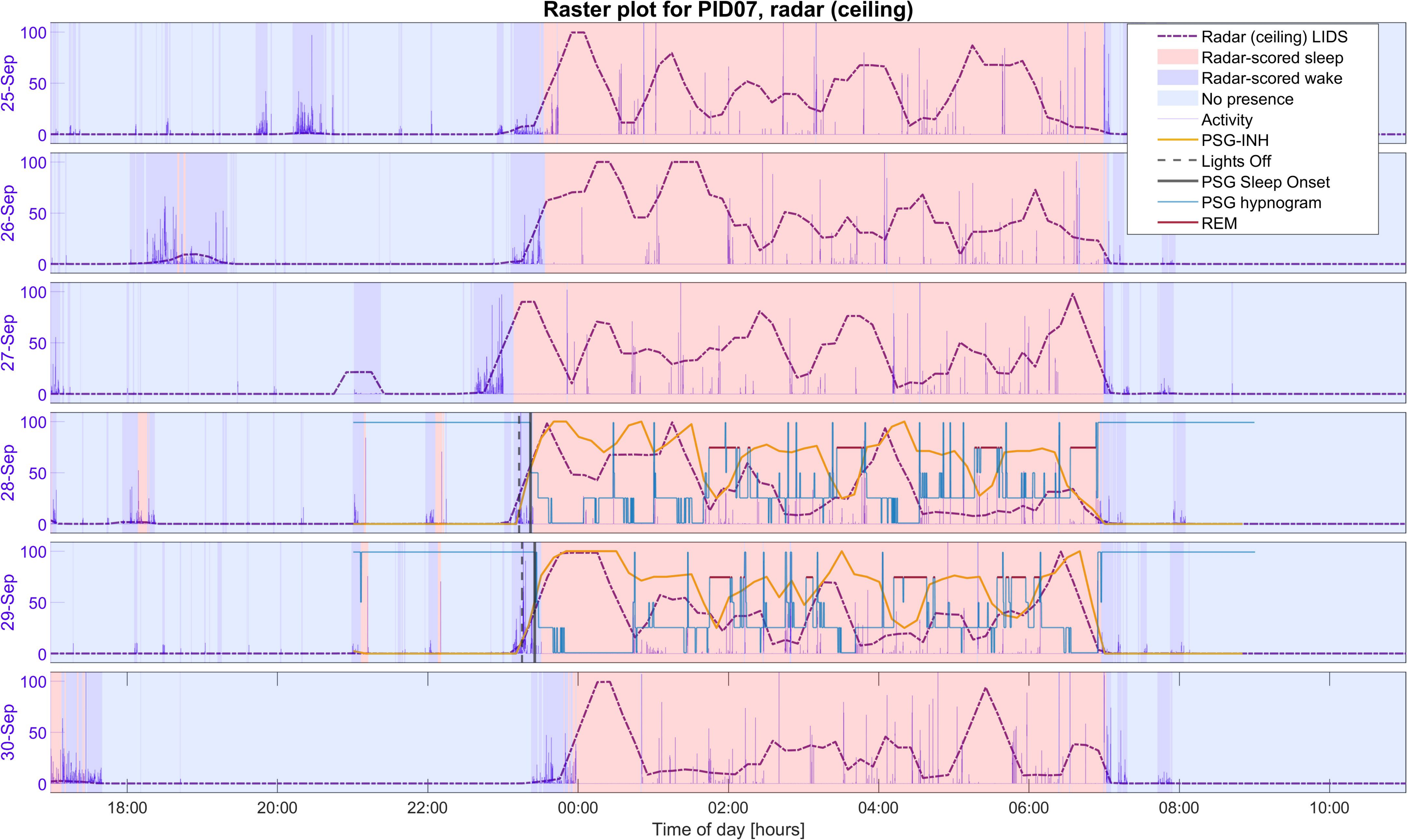

Raster plot for PID07, radar (ceiling)
Radar (ceiling) LIDS
Radar-scored sleep
Radar-scored wake
No presence
Activity
PSG-INH
Lights Off
PSG Sleep Onset
PSG hypnogram
REM
25-Sep
26-Sep
27-Sep
28-Sep
29-Sep
30-Sep
0
50
100
18:00
20:00
22:00
00:00
02:00
04:00
06:00
08:00
10:00
Time of day [hours]

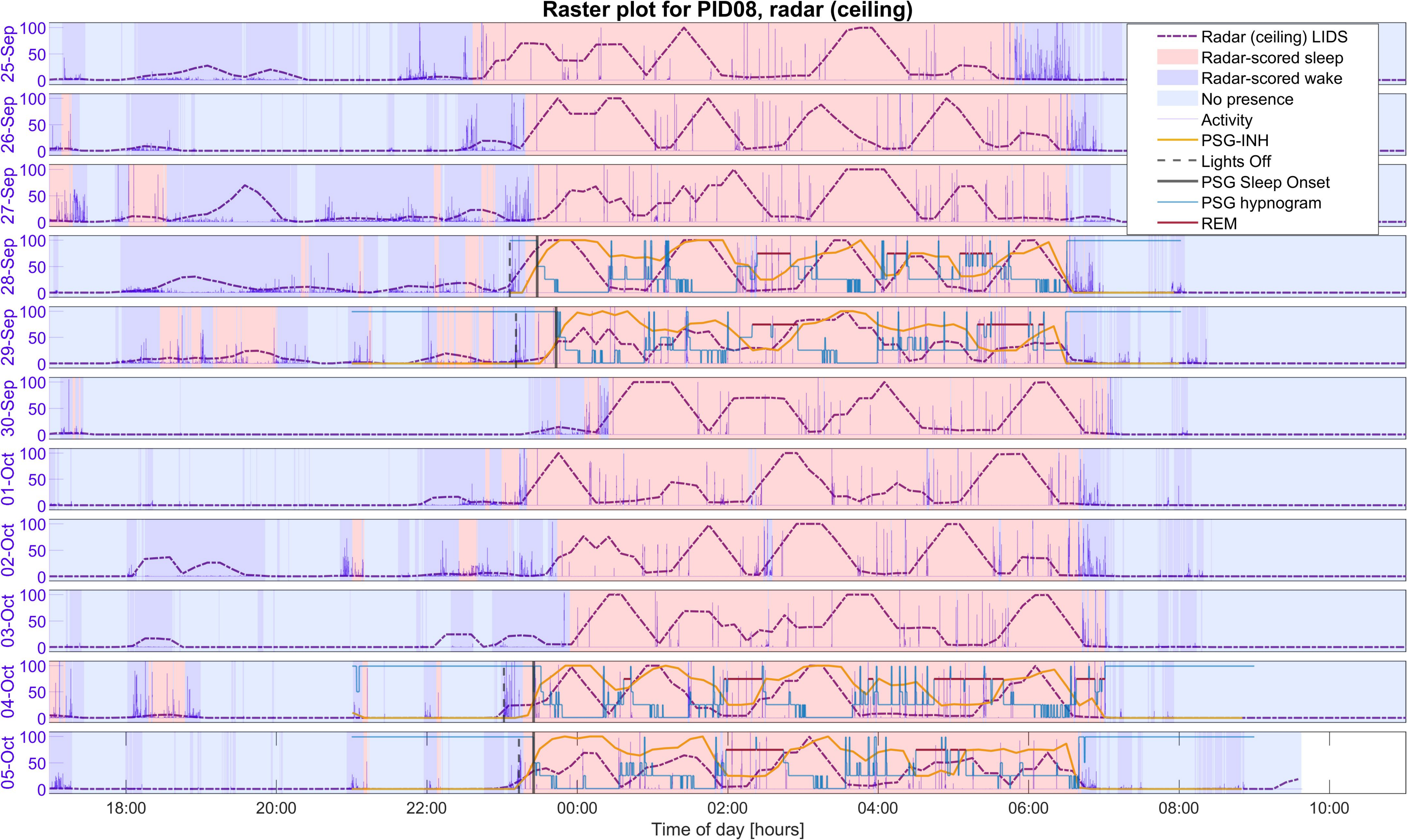

Raster plot for PID08, radar (ceiling)
25-Sep
26-Sep
27-Sep
28-Sep
29-Sep
30-Sep
01-Oct
02-Oct
03-Oct
04-Oct
05-Oct
0
50
100
Radar (ceiling) LIDS
Radar-scored sleep
Radar-scored wake
No presence
Activity
PSG-INH
Lights Off
PSG Sleep Onset
PSG hypnogram
REM
18:00
20:00
22:00
00:00
02:00
04:00
06:00
08:00
10:00
Time of day [hours]

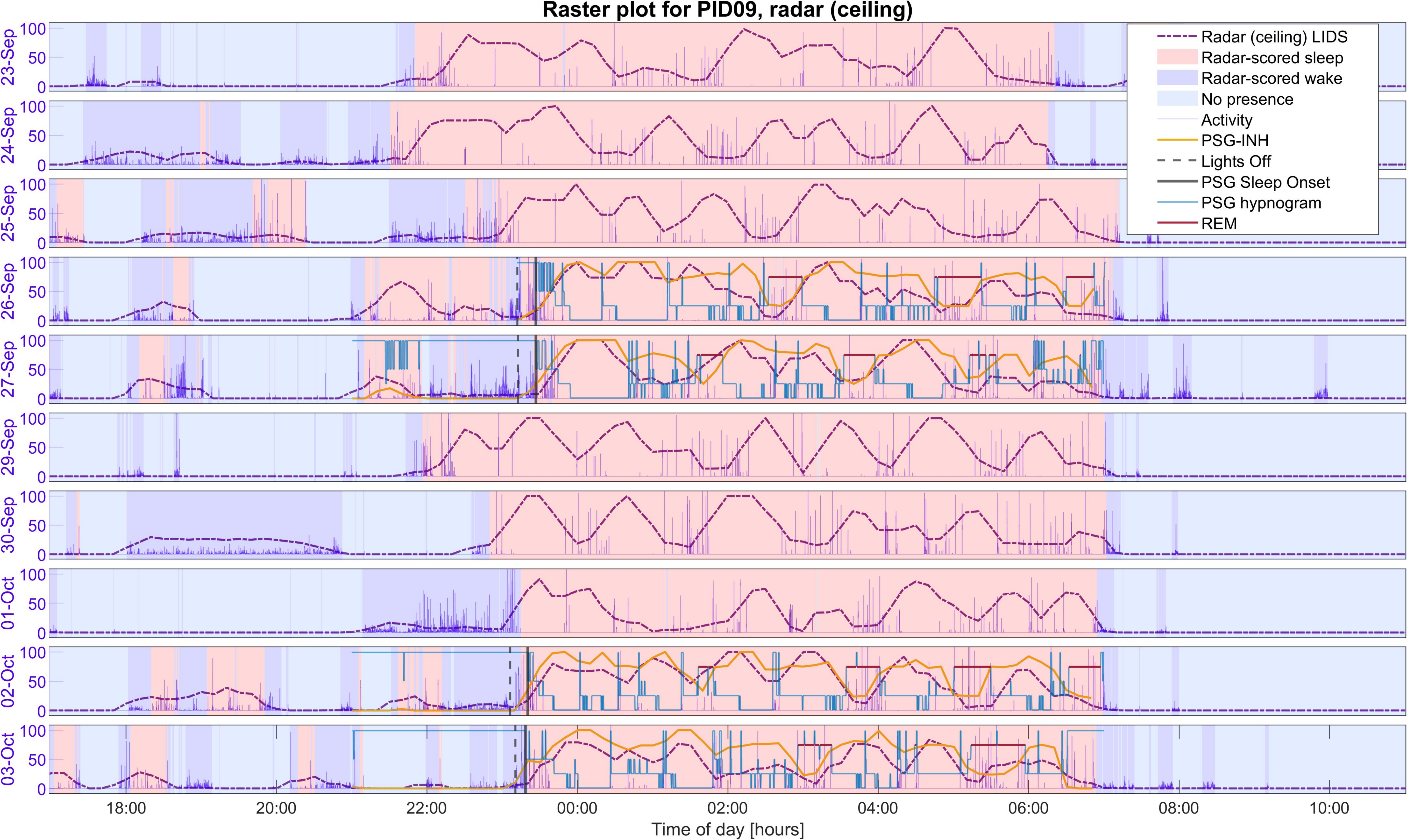

Raster plot for PID09, radar (ceiling)
Radar (ceiling) LIDS
Radar-scored sleep
Radar-scored wake
No presence
Activity
PSG-INH
Lights Off
PSG Sleep Onset
PSG hypnogram
REM
23-Sep
24-Sep
25-Sep
26-Sep
27-Sep
29-Sep
30-Sep
01-Oct
02-Oct
03-Oct
0
50
100
18:00
20:00
22:00
00:00
02:00
04:00
06:00
08:00
10:00
Time of day [hours]

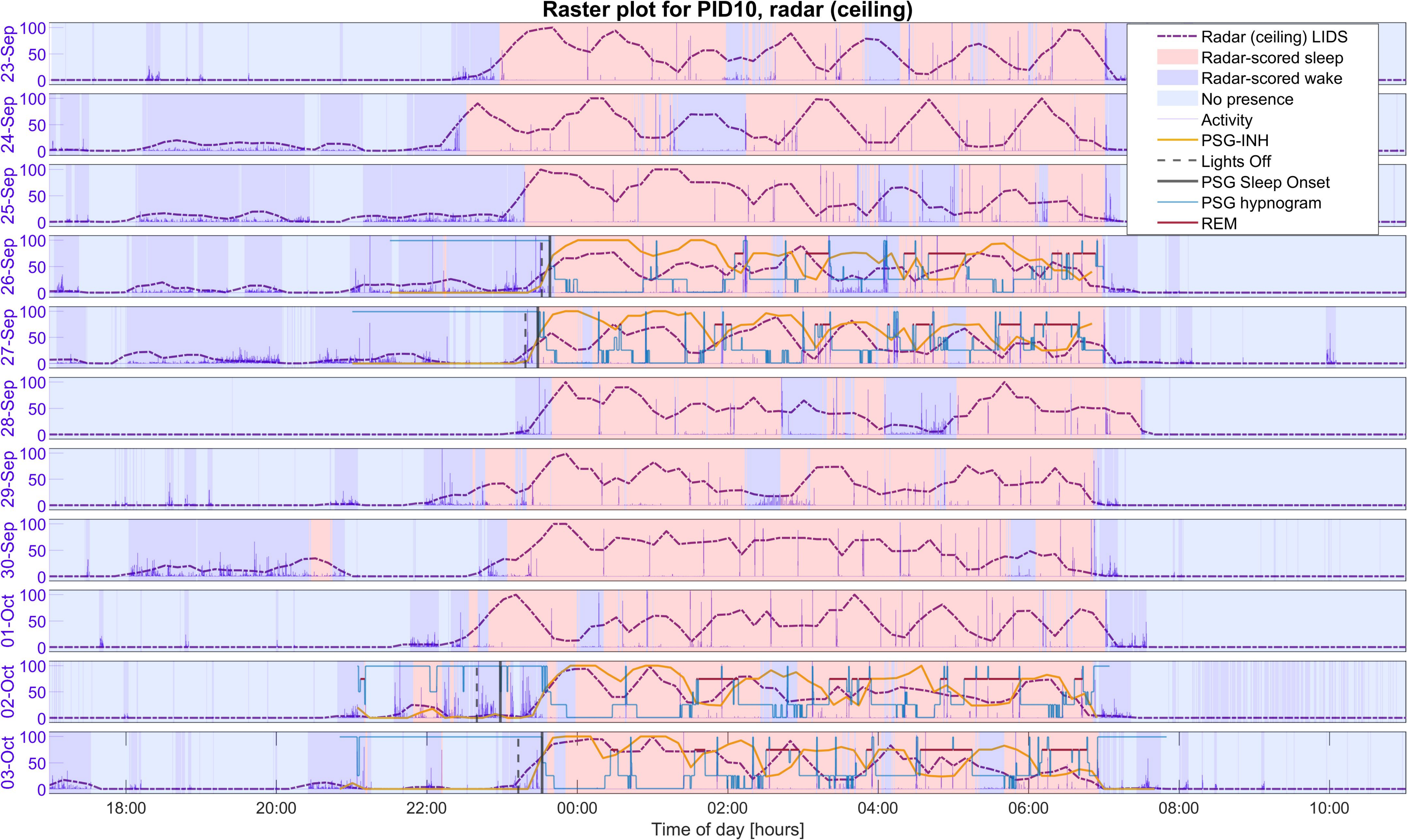

Raster plot for PID10, radar (ceiling)
Radar (ceiling) LIDS
Radar-scored sleep
Radar-scored wake
No presence
Activity
PSG-INH
Lights Off
PSG Sleep Onset
PSG hypnogram
REM
23-Sep
24-Sep
25-Sep
26-Sep
27-Sep
28-Sep
29-Sep
30-Sep
01-Oct
02-Oct
03-Oct
0
50
100
18:00
20:00
22:00
00:00
02:00
04:00
06:00
08:00
10:00
Time of day [hours]

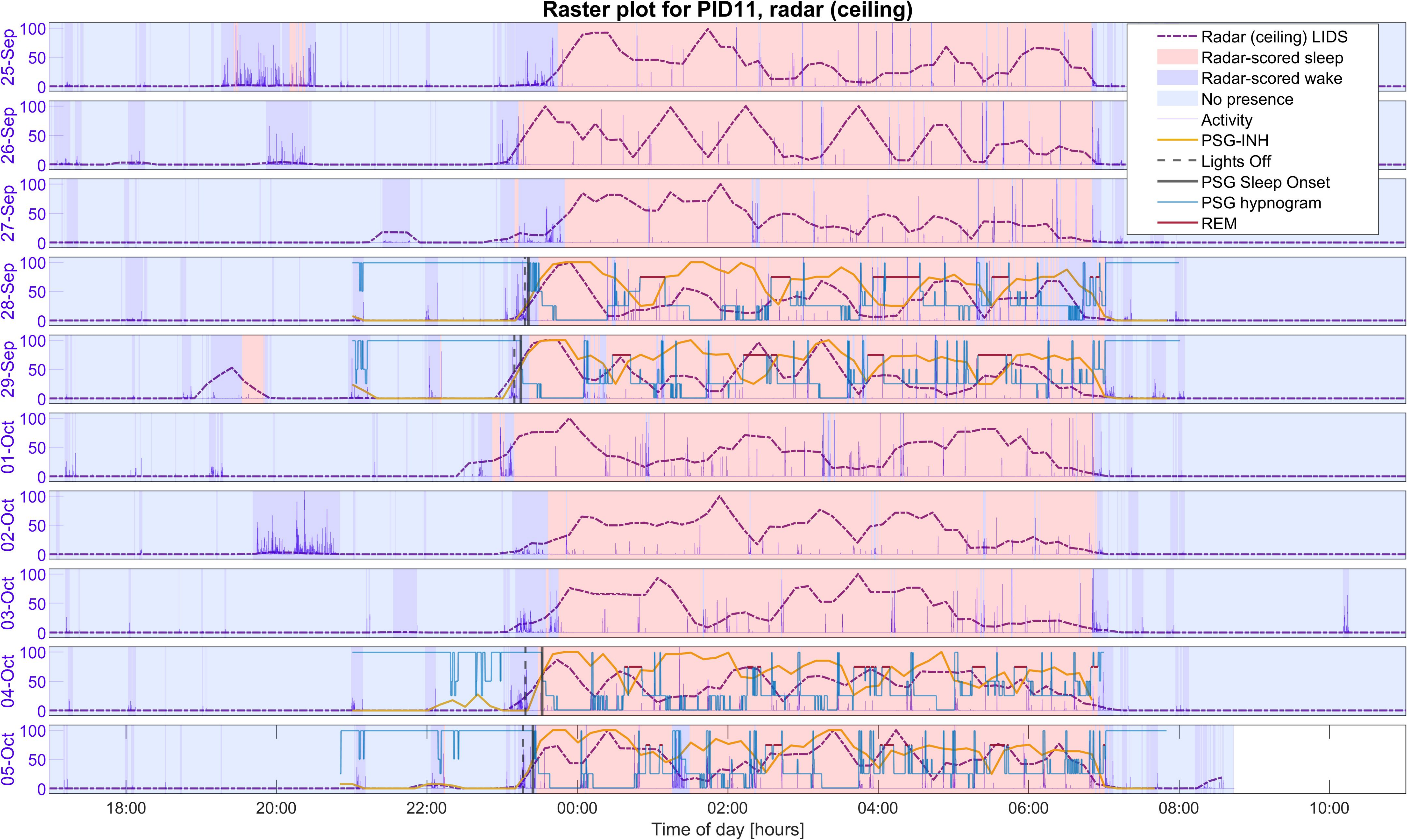

Raster plot for PID11, radar (ceiling)
25-Sep
26-Sep
27-Sep
28-Sep
29-Sep
01-Oct
02-Oct
03-Oct
04-Oct
05-Oct
0
50
100
Radar (ceiling) LIDS
Radar-scored sleep
Radar-scored wake
No presence
Activity
PSG-INH
Lights Off
PSG Sleep Onset
PSG hypnogram
REM
18:00
20:00
22:00
00:00
02:00
04:00
06:00
08:00
10:00
Time of day [hours]

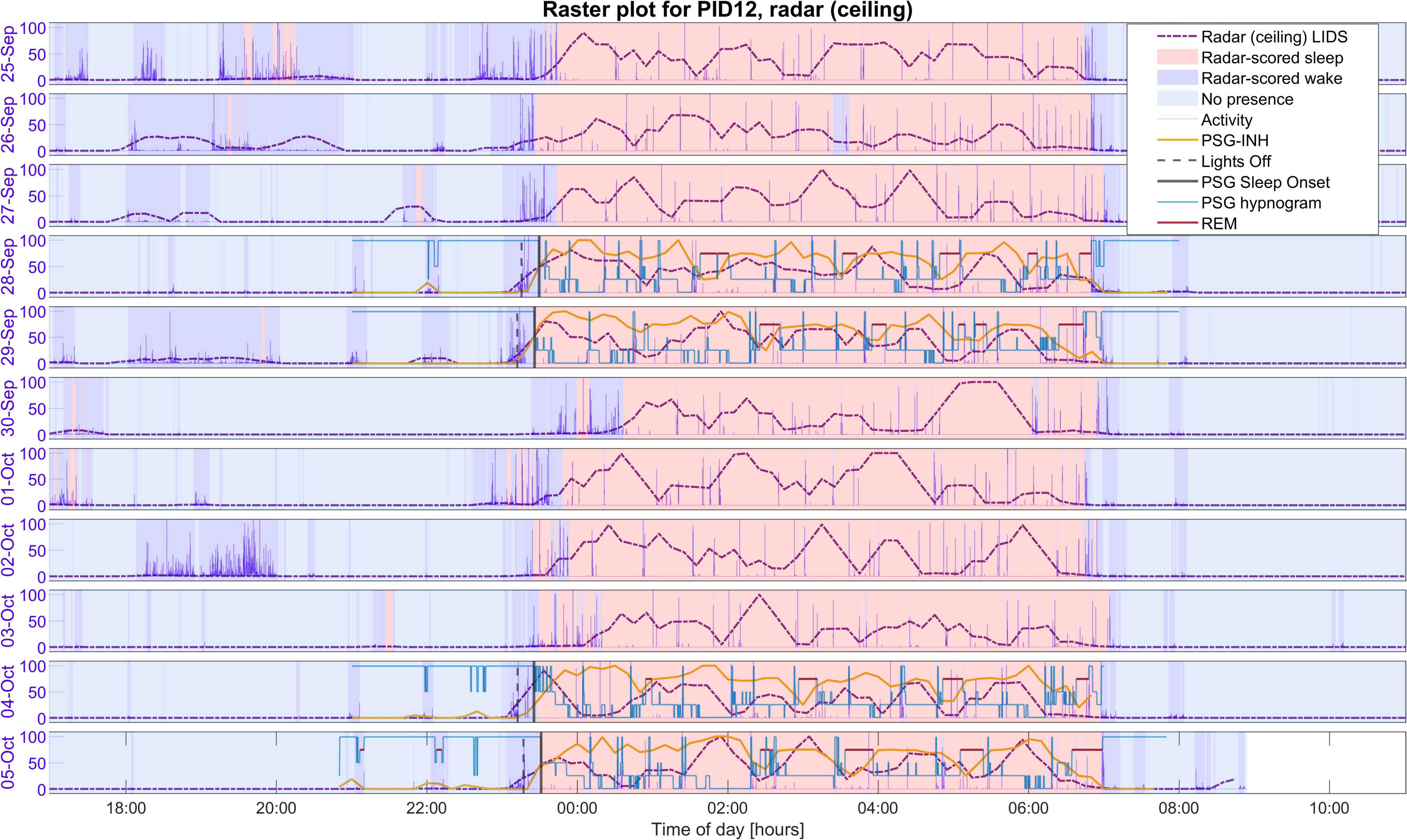

Raster plot for PID12, radar (ceiling)
Radar (ceiling) LIDS
Radar-scored sleep
Radar-scored wake
No presence
Activity
PSG-INH
Lights Off
PSG Sleep Onset
PSG hypnogram
REM
25-Sep
26-Sep
27-Sep
28-Sep
29-Sep
30-Sep
01-Oct
02-Oct
03-Oct
04-Oct
05-Oct
0
50
100
18:00
20:00
22:00
00:00
02:00
04:00
06:00
08:00
10:00
Time of day [hours]

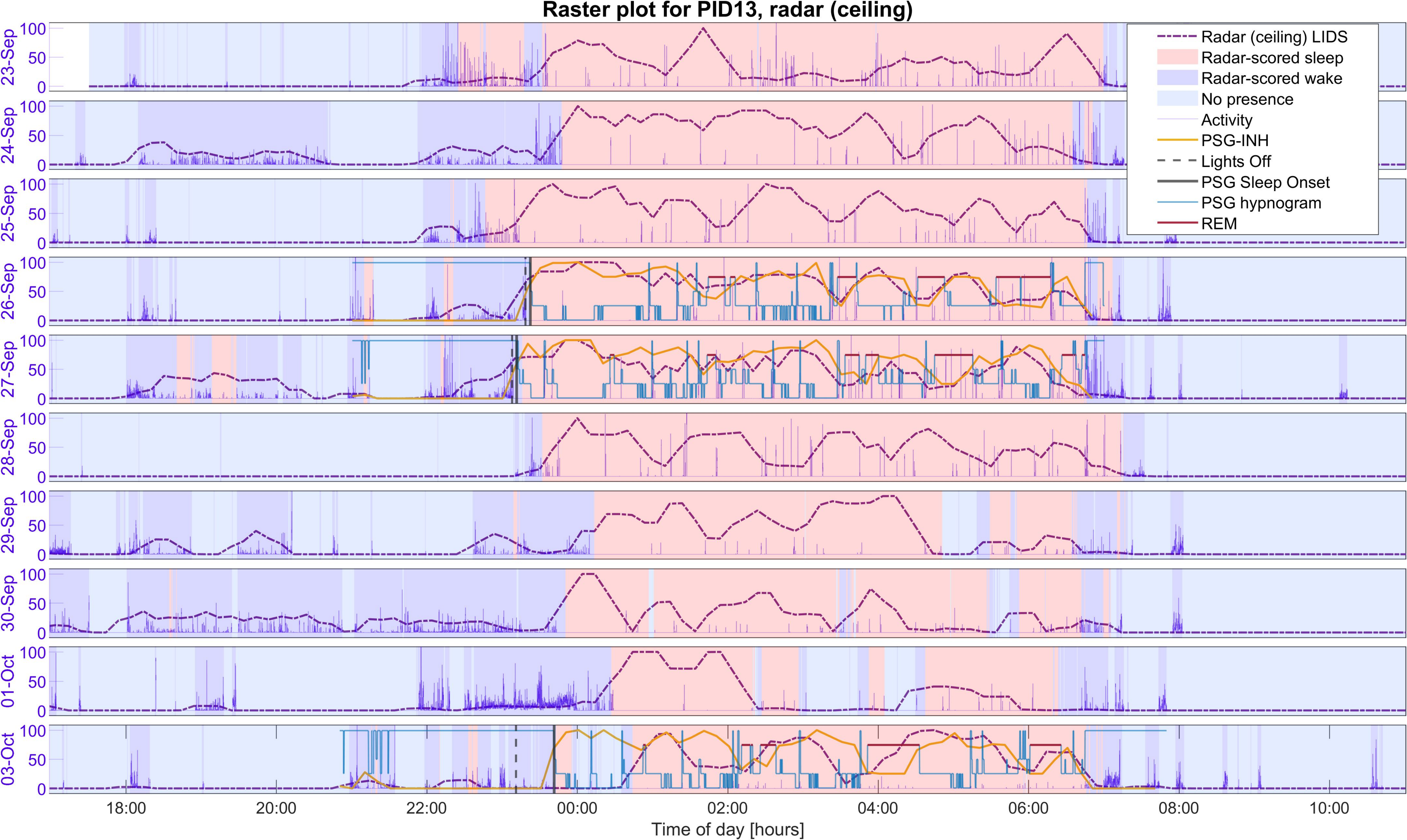

Raster plot for PID13, radar (ceiling)
23-Sep
24-Sep
25-Sep
26-Sep
27-Sep
28-Sep
29-Sep
30-Sep
01-Oct
03-Oct
0
50
100
Radar (ceiling) LIDS
Radar-scored sleep
Radar-scored wake
No presence
Activity
PSG-INH
Lights Off
PSG Sleep Onset
PSG hypnogram
REM
18:00
20:00
22:00
00:00
02:00
04:00
06:00
08:00
10:00
Time of day [hours]

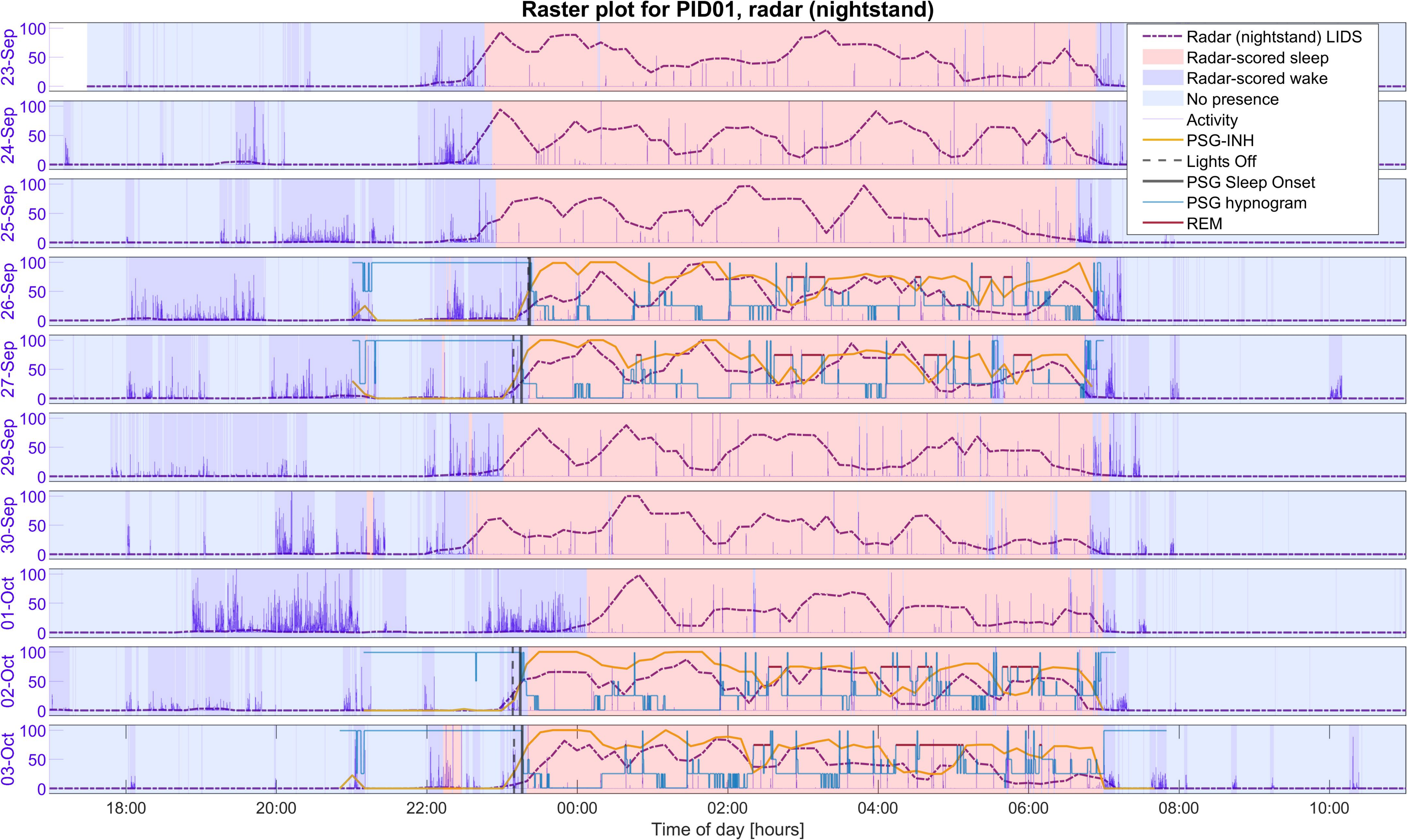

Raster plot for PID01, radar (nightstand)
Radar (nightstand) LIDS
Radar-scored sleep
Radar-scored wake
No presence
Activity
PSG-INH
Lights Off
PSG Sleep Onset
PSG hypnogram
REM
23-Sep
24-Sep
25-Sep
26-Sep
27-Sep
29-Sep
30-Sep
01-Oct
02-Oct
03-Oct
0
50
100
18:00
20:00
22:00
00:00
02:00
04:00
06:00
08:00
10:00
Time of day [hours]

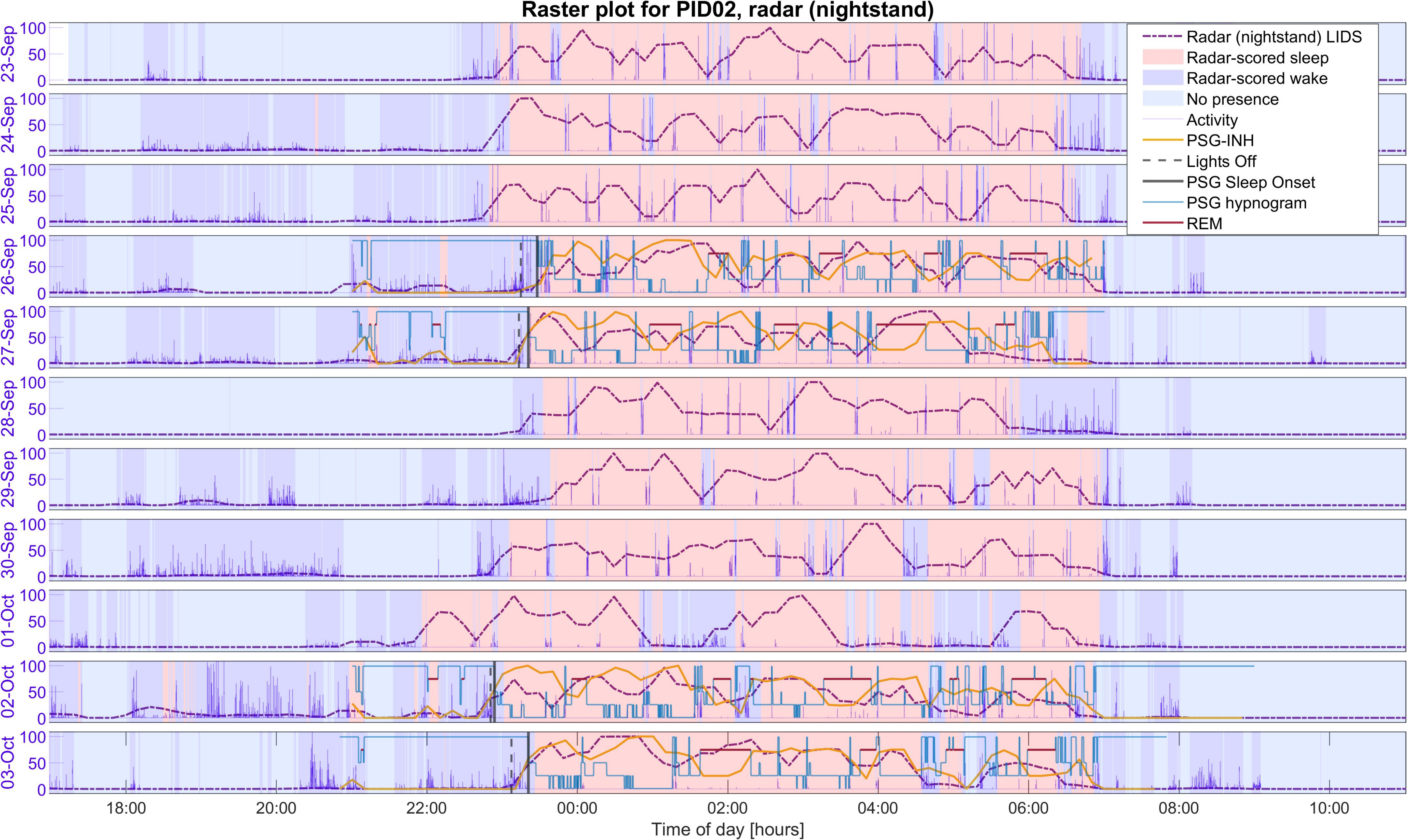
Raster plot for PID02, radar (nightstand)
23-Sep
24-Sep
25-Sep
26-Sep
27-Sep
28-Sep
29-Sep
30-Sep
01-Oct
02-Oct
03-Oct
0
50
100
Radar (nightstand) LIDS
Radar-scored sleep
Radar-scored wake
No presence
Activity
PSG-INH
Lights Off
PSG Sleep Onset
PSG hypnogram
REM
18:00
20:00
22:00
00:00
02:00
04:00
06:00
08:00
10:00
Time of day [hours]

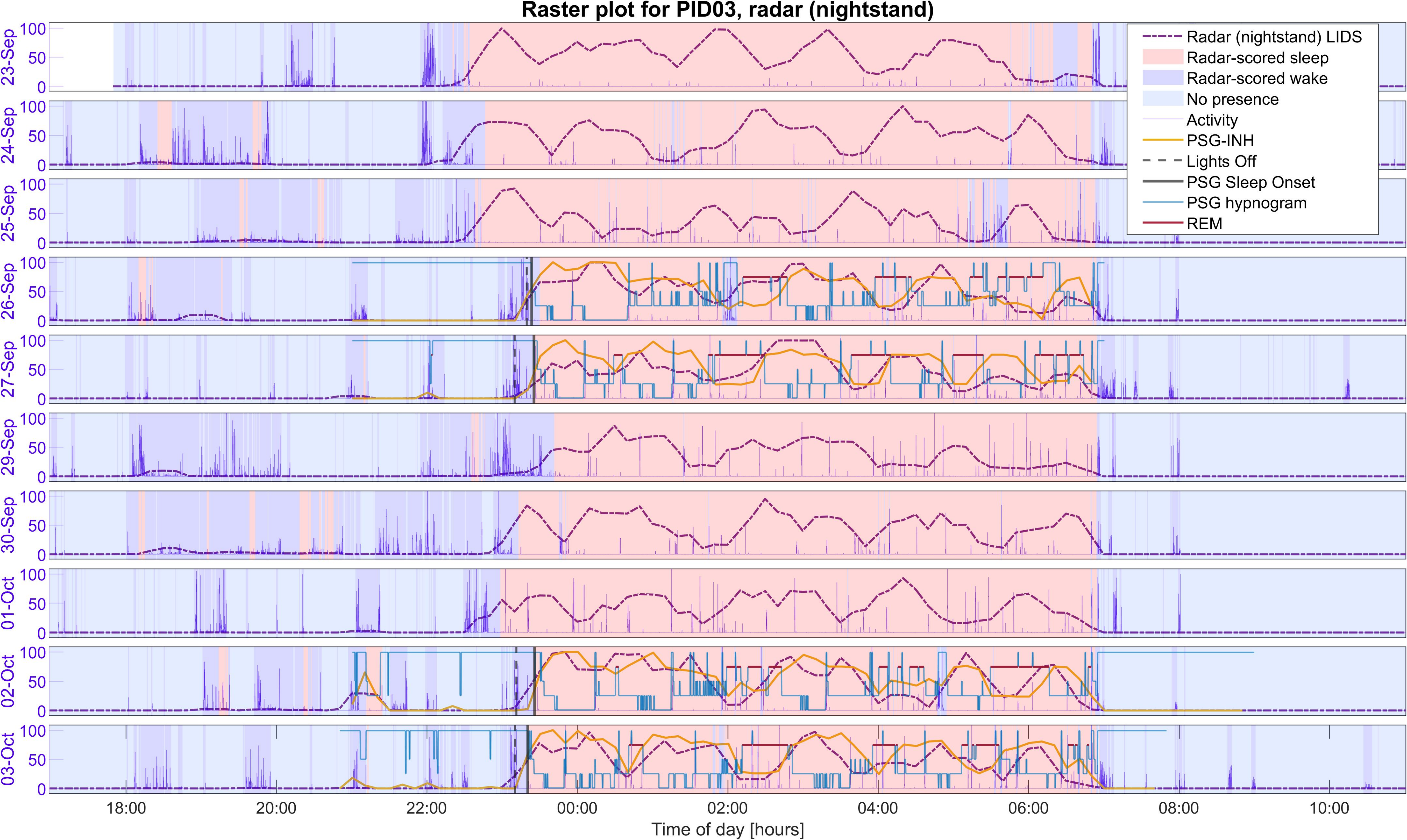

Raster plot for PID03, radar (nightstand)
Radar (nightstand) LIDS
Radar-scored sleep
Radar-scored wake
No presence
Activity
PSG-INH
Lights Off
PSG Sleep Onset
PSG hypnogram
REM
23-Sep
24-Sep
25-Sep
26-Sep
27-Sep
29-Sep
30-Sep
01-Oct
02-Oct
03-Oct
0
50
100
18:00
20:00
22:00
00:00
02:00
04:00
06:00
08:00
10:00
Time of day [hours]

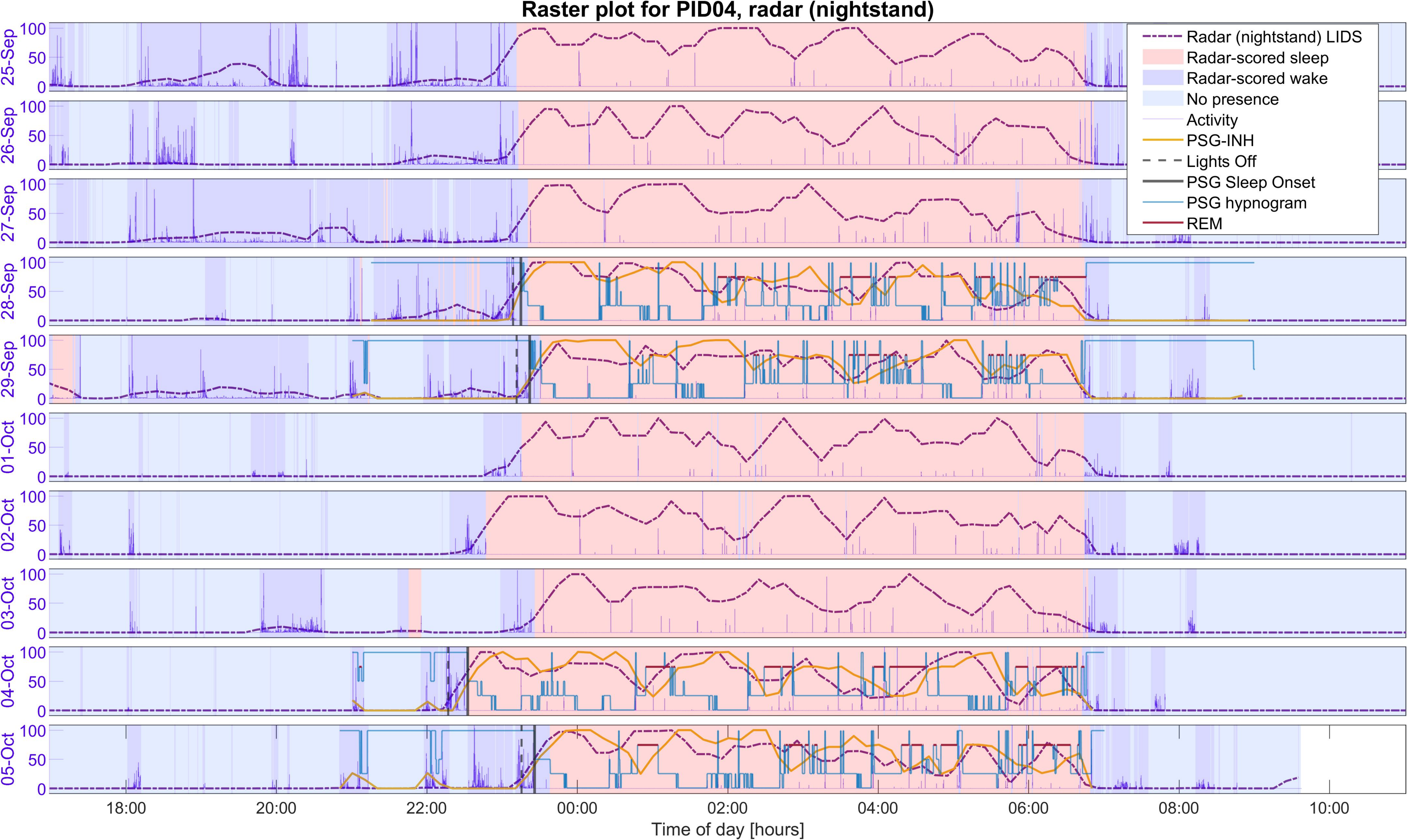

Raster plot for PID04, radar (nightstand)
Radar (nightstand) LIDS
Radar-scored sleep
Radar-scored wake
No presence
Activity
PSG-INH
Lights Off
PSG Sleep Onset
PSG hypnogram
REM
25-Sep
26-Sep
27-Sep
28-Sep
29-Sep
01-Oct
02-Oct
03-Oct
04-Oct
05-Oct
0
50
100
18:00
20:00
22:00
00:00
02:00
04:00
06:00
08:00
10:00
Time of day [hours]

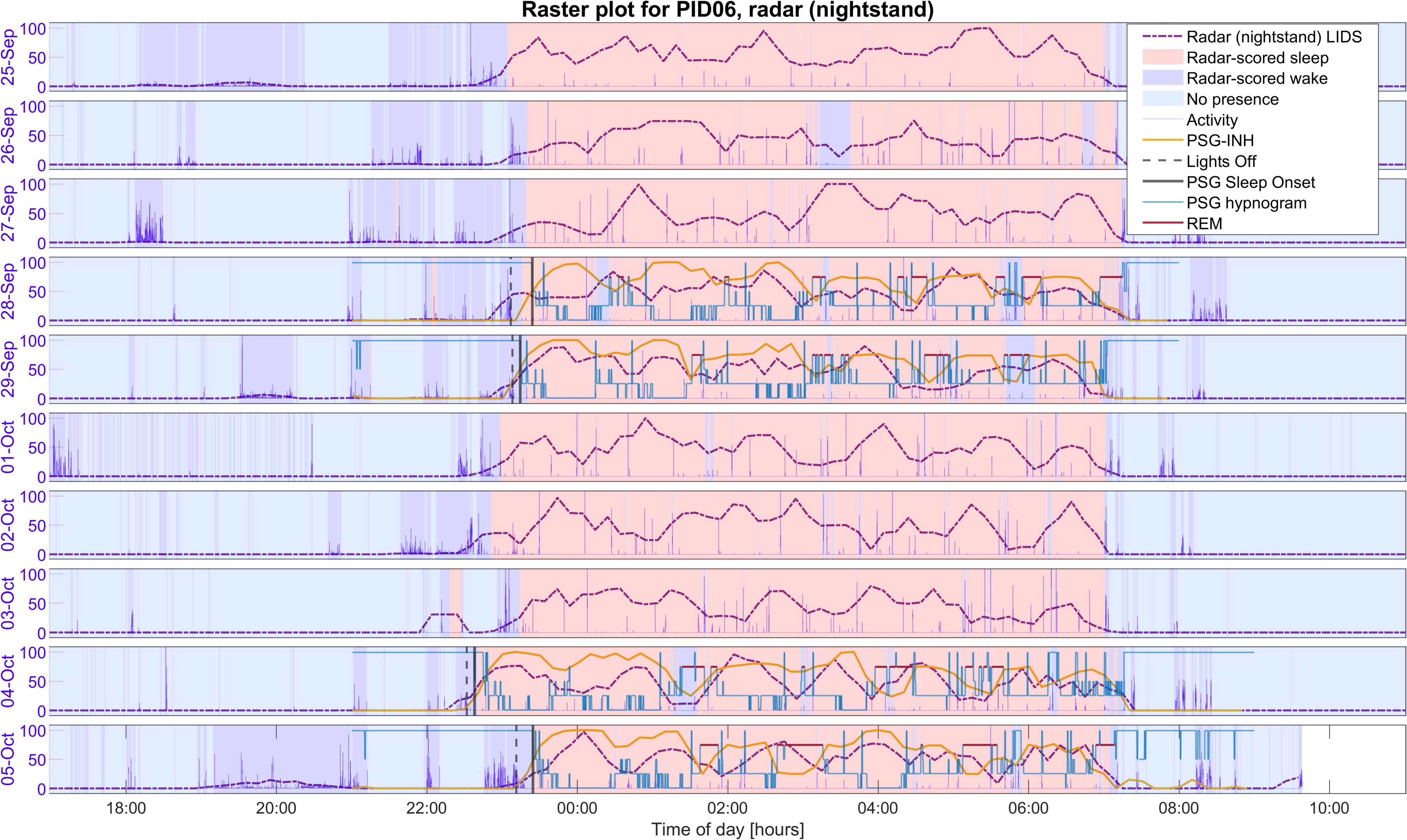

Raster plot for PID06, radar (nightstand)
25-Sep
26-Sep
27-Sep
28-Sep
29-Sep
01-Oct
02-Oct
03-Oct
04-Oct
05-Oct
0
50
100
Radar (nightstand) LIDS
Radar-scored sleep
Radar-scored wake
No presence
Activity
PSG-INH
Lights Off
PSG Sleep Onset
PSG hypnogram
REM
18:00
20:00
22:00
00:00
02:00
04:00
06:00
08:00
10:00
Time of day [hours]

Raster plot for PID07, radar (nightstand)

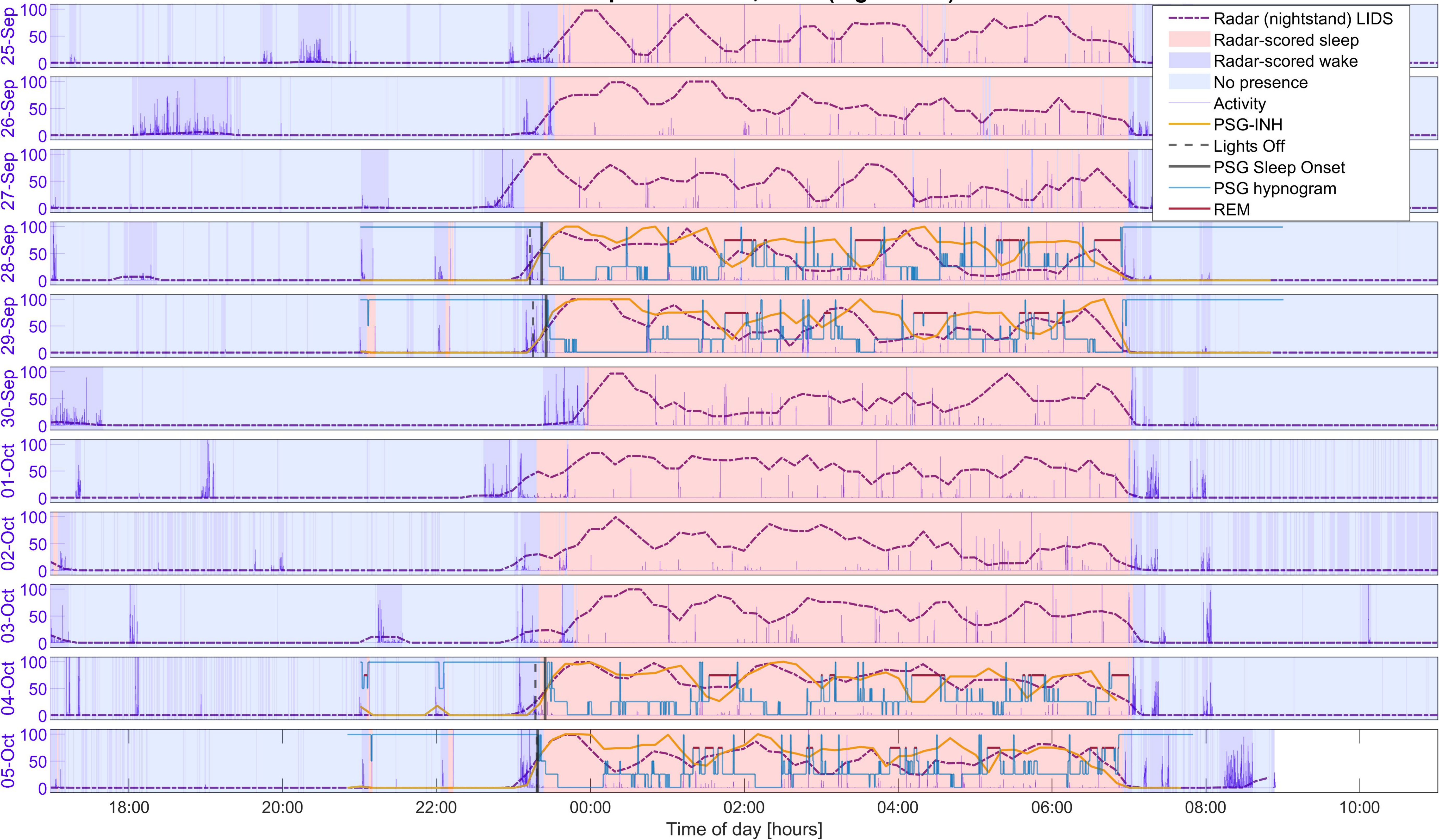

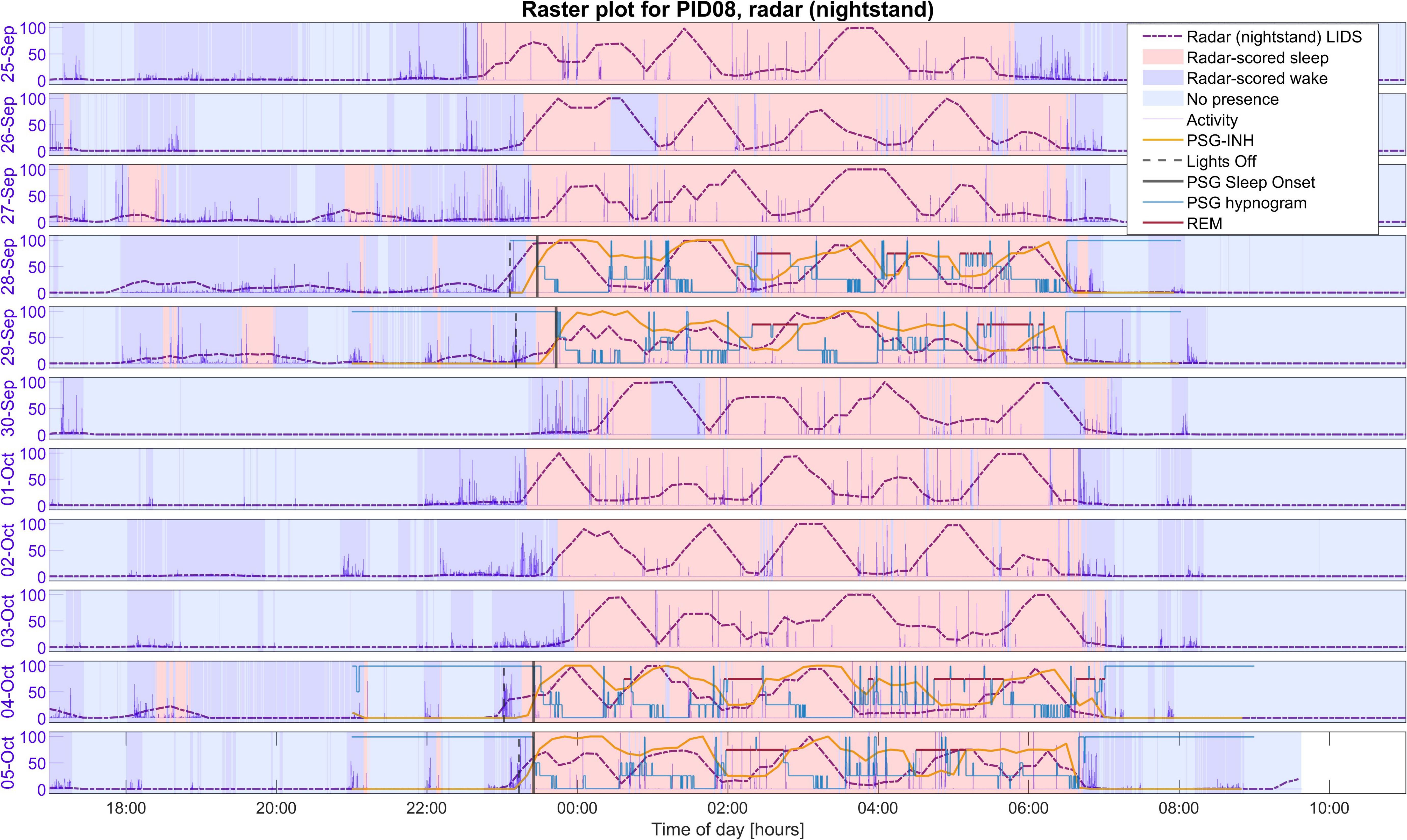

Raster plot for PID08, radar (nightstand)
Radar (nightstand) LIDS
Radar-scored sleep
Radar-scored wake
No presence
Activity
PSG-INH
Lights Off
PSG Sleep Onset
PSG hypnogram
REM
25-Sep
26-Sep
27-Sep
28-Sep
29-Sep
30-Sep
01-Oct
02-Oct
03-Oct
04-Oct
05-Oct
0
50
100
18:00
20:00
22:00
00:00
02:00
04:00
06:00
08:00
10:00
Time of day [hours]

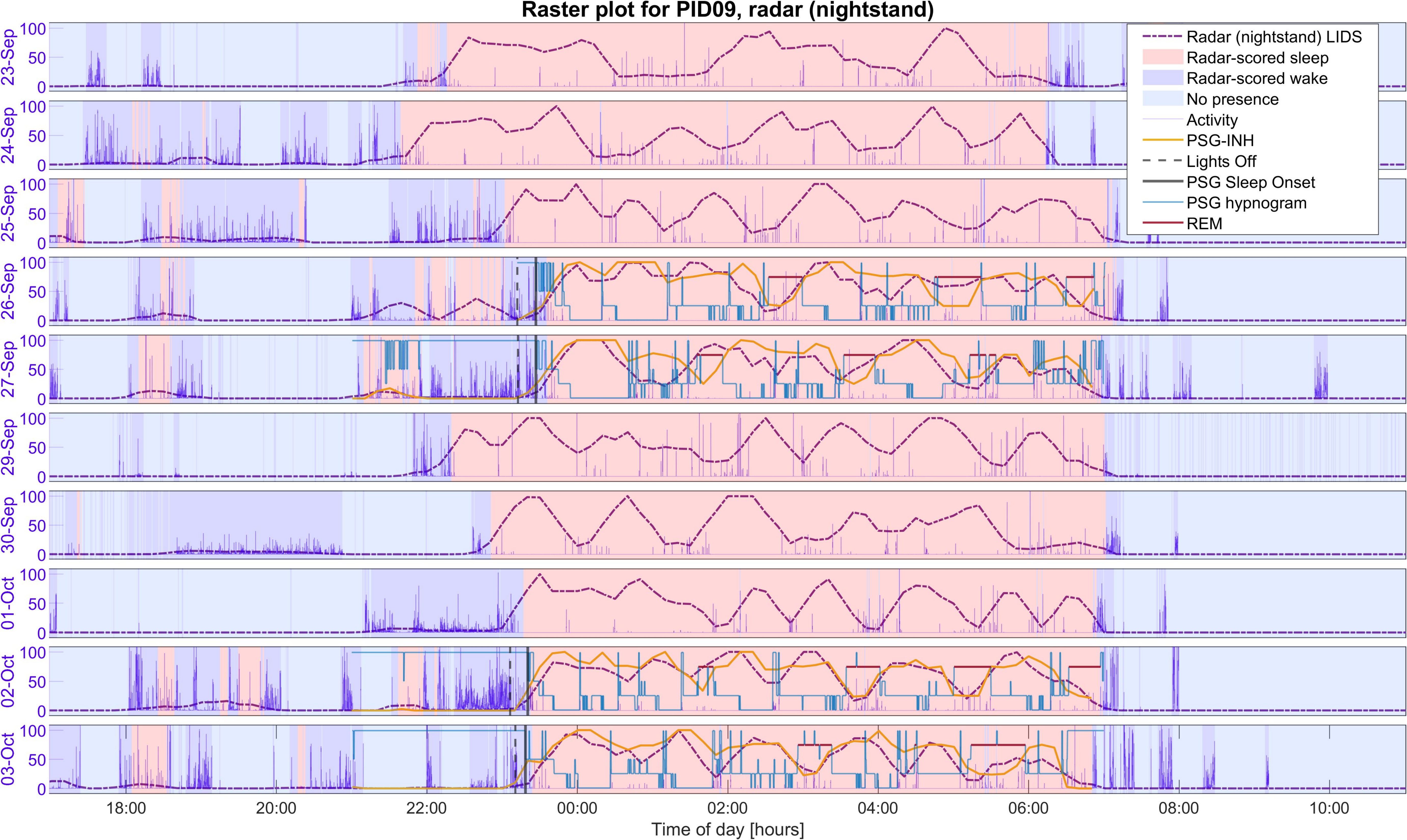

Raster plot for PID09, radar (nightstand)
Radar (nightstand) LIDS
Radar-scored sleep
Radar-scored wake
No presence
Activity
PSG-INH
Lights Off
PSG Sleep Onset
PSG hypnogram
REM
23-Sep
24-Sep
25-Sep
26-Sep
27-Sep
29-Sep
30-Sep
01-Oct
02-Oct
03-Oct
0
50
100
18:00
20:00
22:00
00:00
02:00
04:00
06:00
08:00
10:00
Time of day [hours]

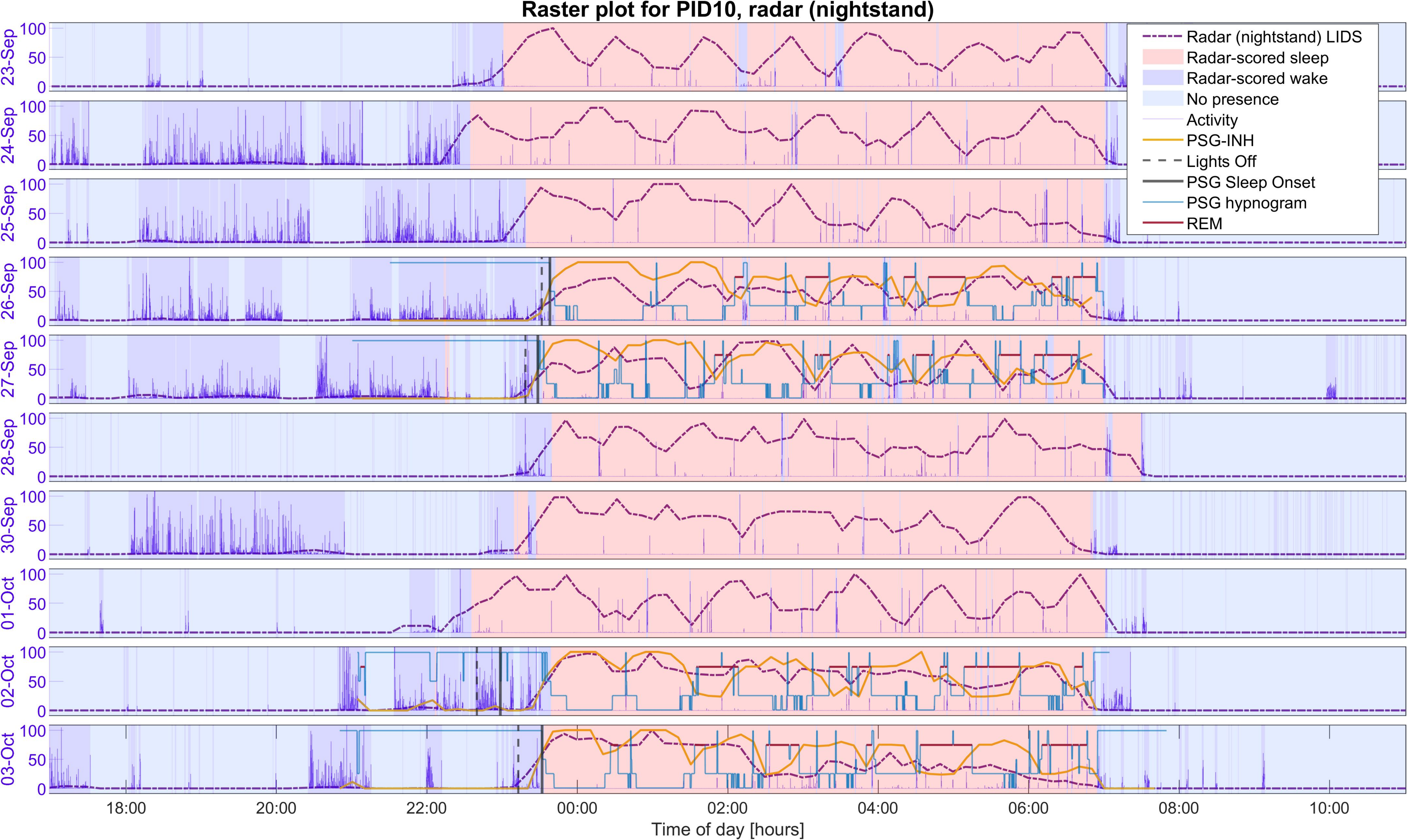

Raster plot for PID10, radar (nightstand)
Radar (nightstand) LIDS
Radar-scored sleep
Radar-scored wake
No presence
Activity
PSG-INH
Lights Off
PSG Sleep Onset
PSG hypnogram
REM
23-Sep
24-Sep
25-Sep
26-Sep
27-Sep
28-Sep
30-Sep
01-Oct
02-Oct
03-Oct
0
50
100
18:00
20:00
22:00
00:00
02:00
04:00
06:00
08:00
10:00
Time of day [hours]

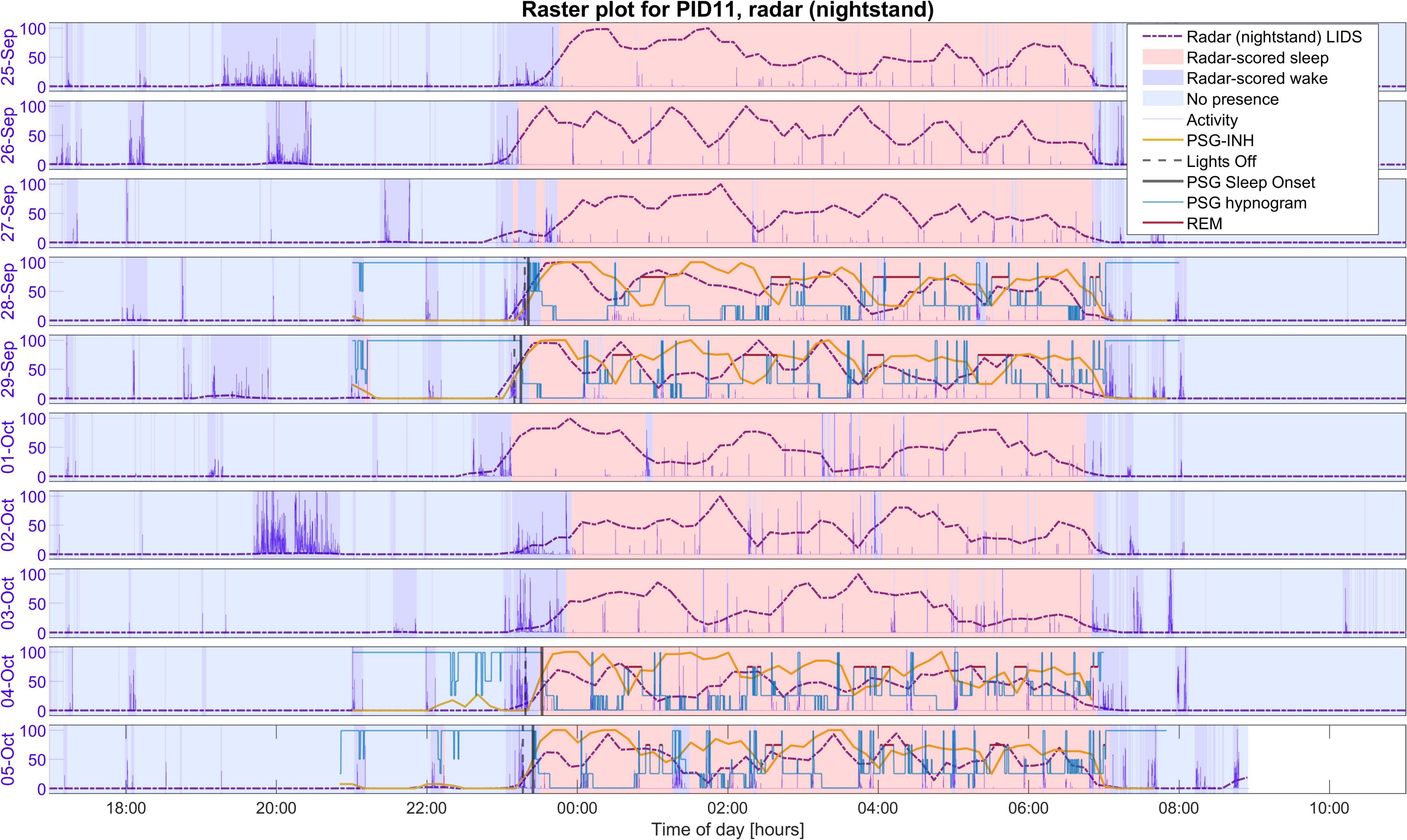
Raster plot for PID11, radar (nightstand)
Radar (nightstand) LIDS
Radar-scored sleep
Radar-scored wake
No presence
Activity
PSG-INH
Lights Off
PSG Sleep Onset
PSG hypnogram
REM
25-Sep
26-Sep
27-Sep
28-Sep
29-Sep
01-Oct
02-Oct
03-Oct
04-Oct
05-Oct
0
50
100
18:00
20:00
22:00
00:00
02:00
04:00
06:00
08:00
10:00
Time of day [hours]

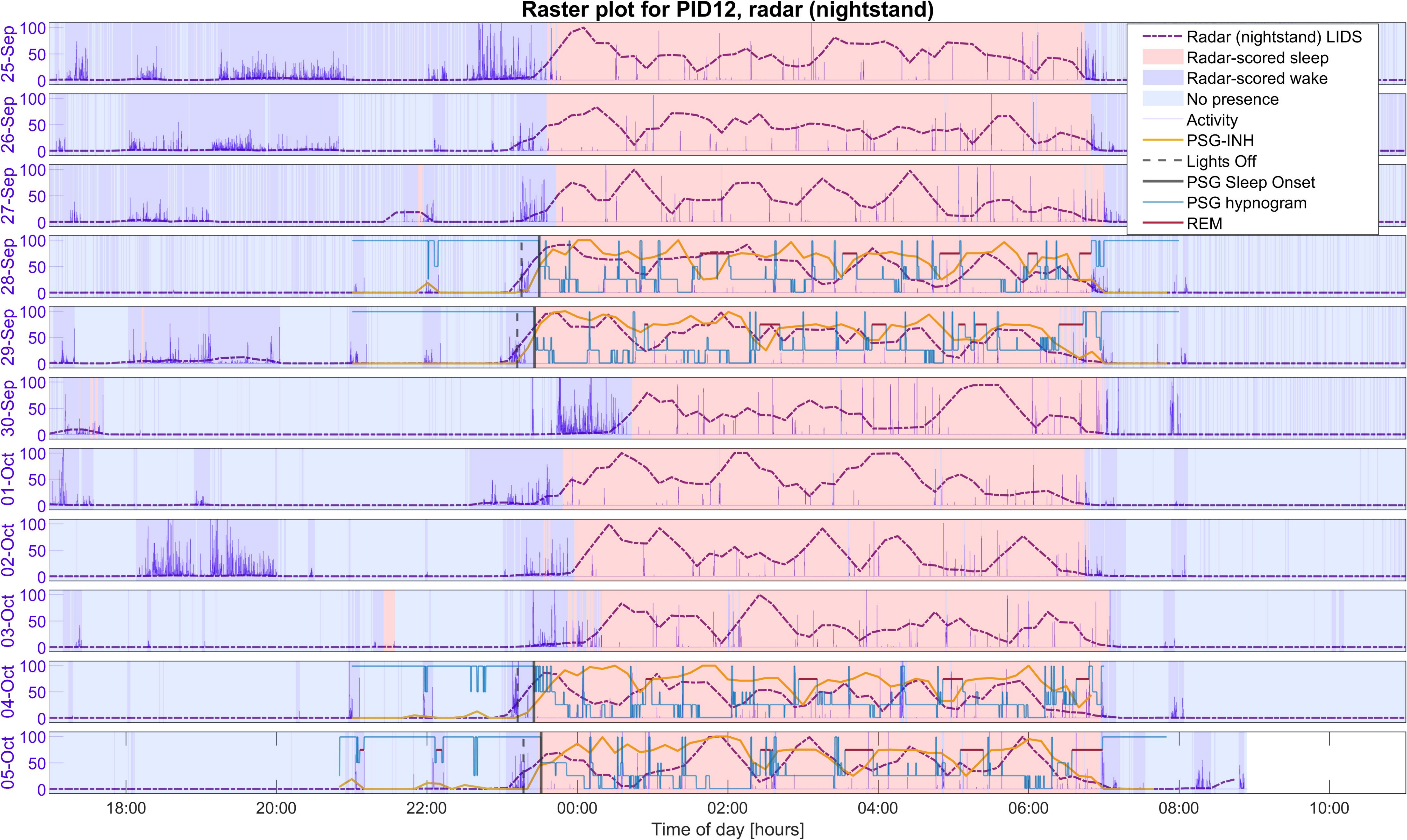

Raster plot for PID12, radar (nightstand)
Radar (nightstand) LIDS
Radar-scored sleep
Radar-scored wake
No presence
Activity
PSG-INH
Lights Off
PSG Sleep Onset
PSG hypnogram
REM
25-Sep
26-Sep
27-Sep
28-Sep
29-Sep
30-Sep
01-Oct
02-Oct
03-Oct
04-Oct
05-Oct
0
50
100
18:00
20:00
22:00
00:00
02:00
04:00
06:00
08:00
10:00
Time of day [hours]

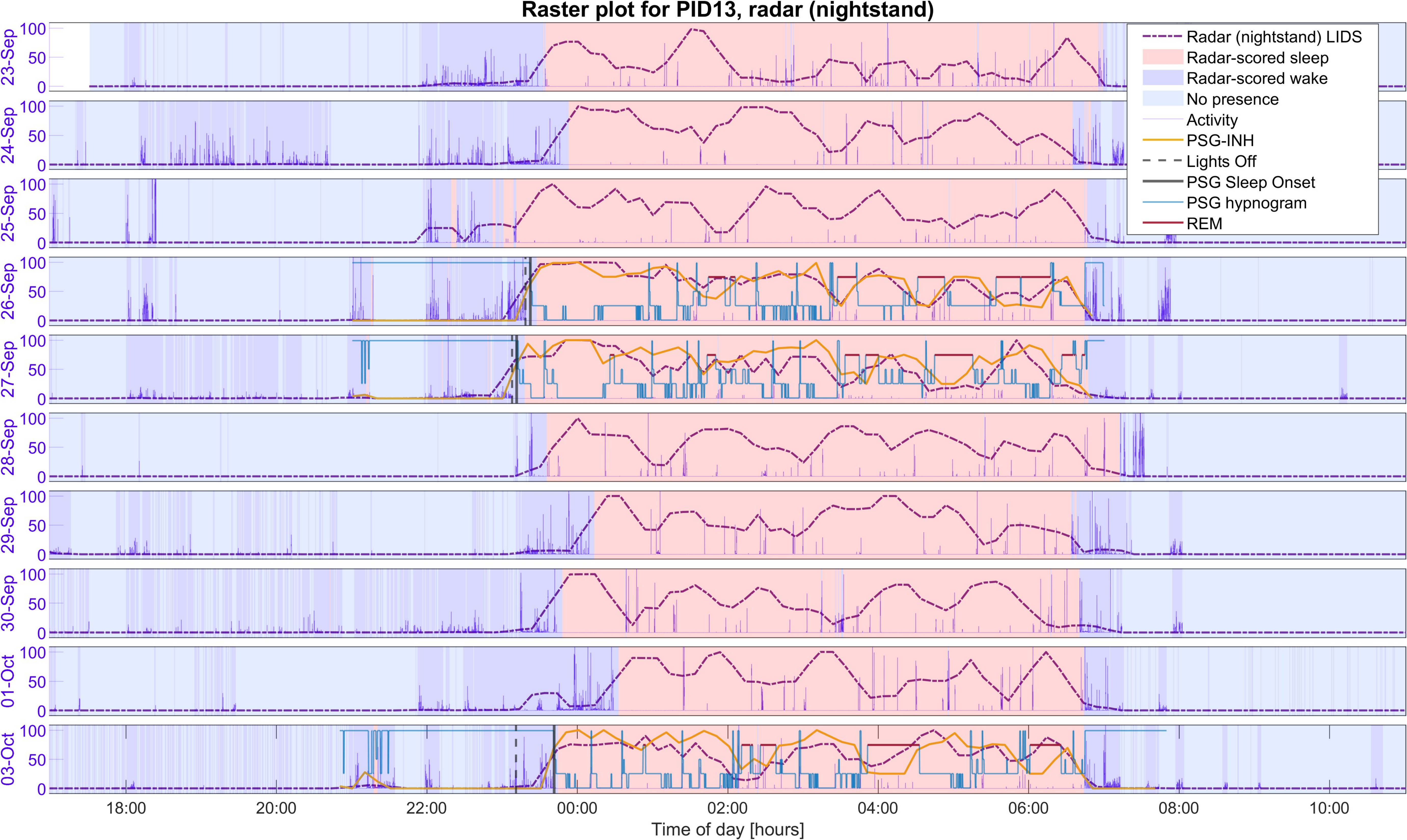

Raster plot for PID13, radar (nightstand)
Radar (nightstand) LIDS
Radar-scored sleep
Radar-scored wake
No presence
Activity
PSG-INH
Lights Off
PSG Sleep Onset
PSG hypnogram
REM
23-Sep
24-Sep
25-Sep
26-Sep
27-Sep
28-Sep
29-Sep
30-Sep
01-Oct
03-Oct
0
50
100
18:00
20:00
22:00
00:00
02:00
04:00
06:00
08:00
10:00
Time of day [hours]
