# Supplementary figures and images for: Contact‐free radar recordings of body movement can reflect ultradian dynamics of sleep
Source: J Sleep Res. 2022 Jul 6;31(6):e13687. doi: 10.1111/jsr.13687 (PMC9786343; doi:10.1111/jsr.13687)

# PID01-20170926

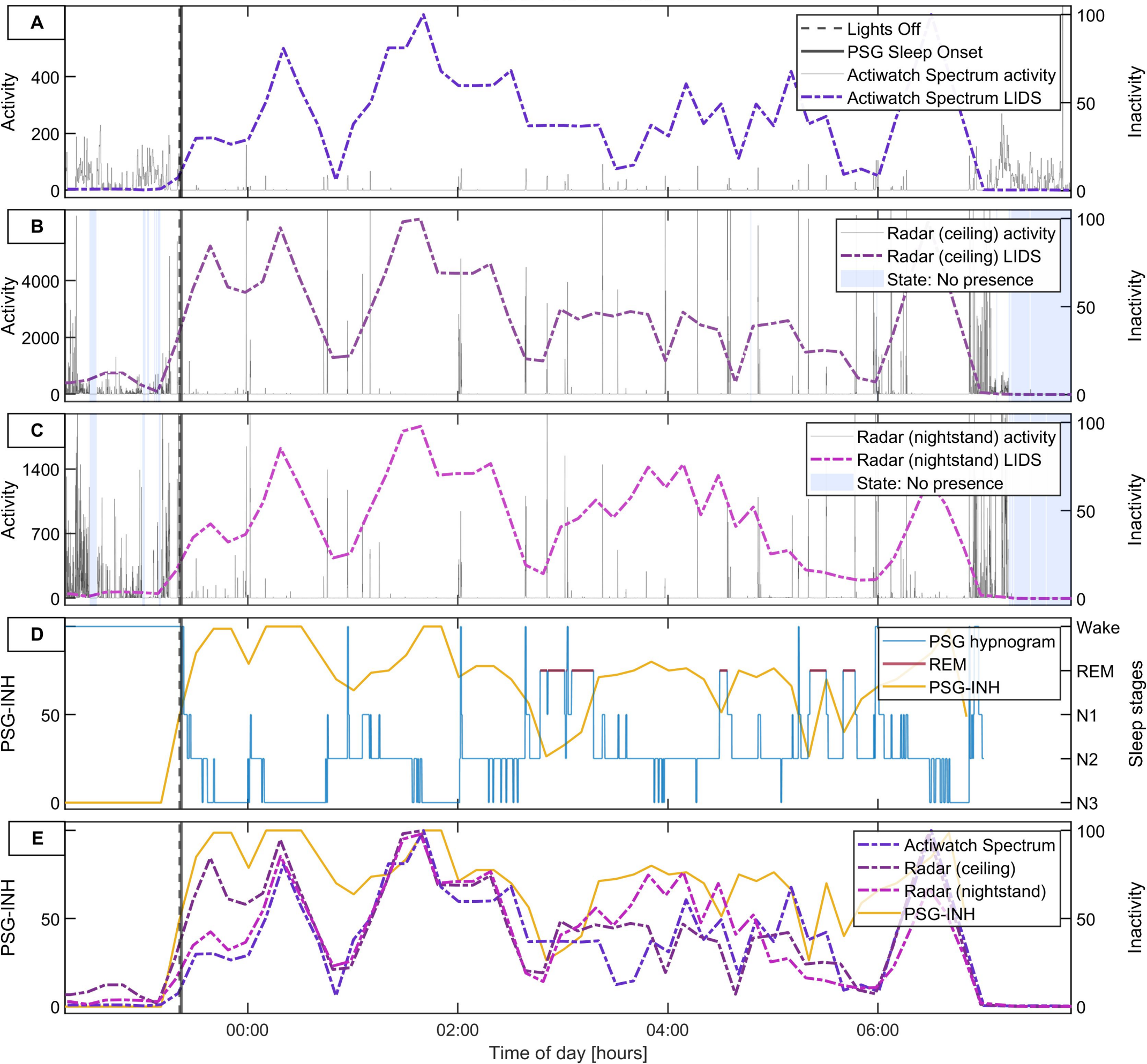

# PID01-20170927

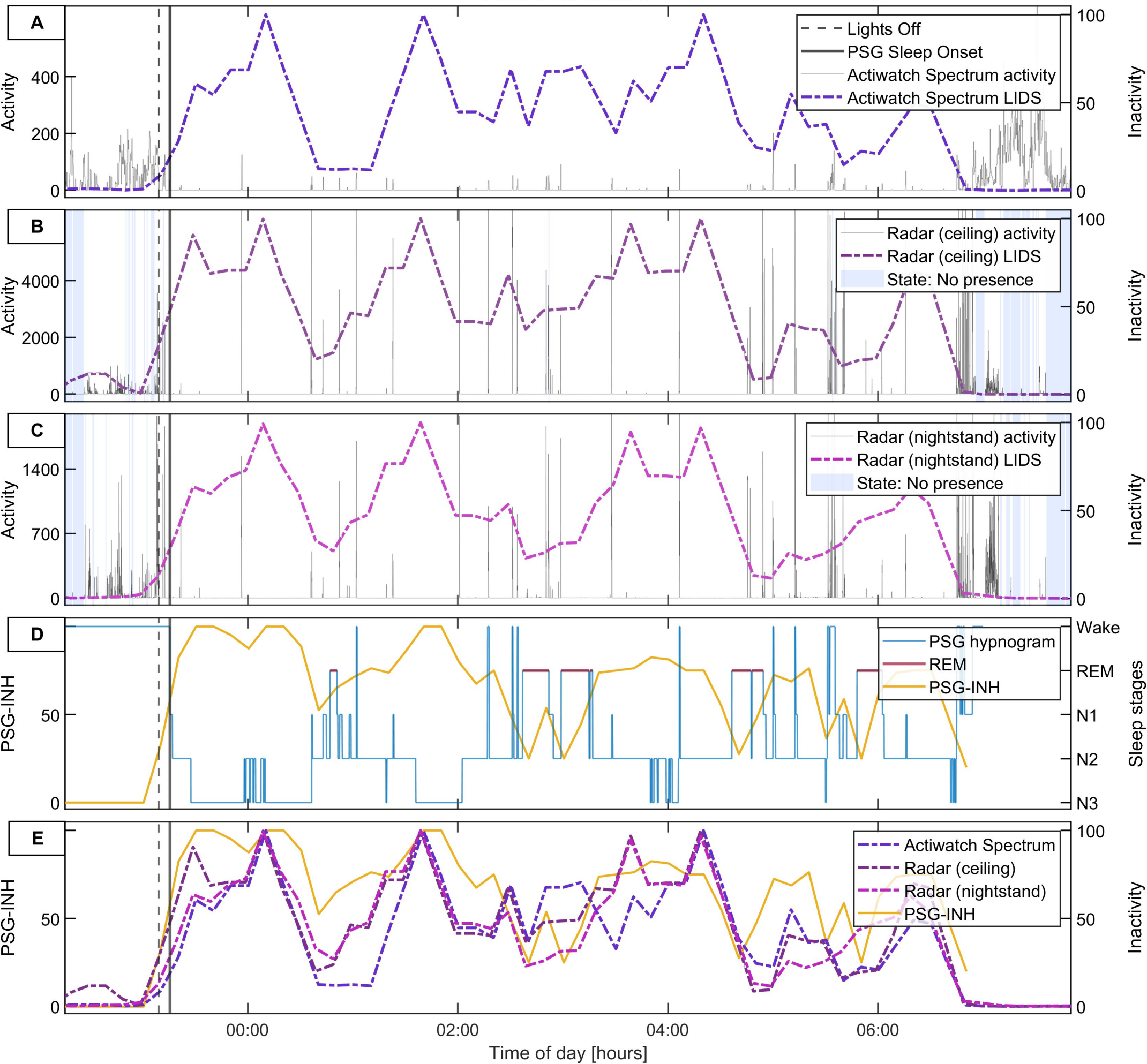

# PID01-20171002

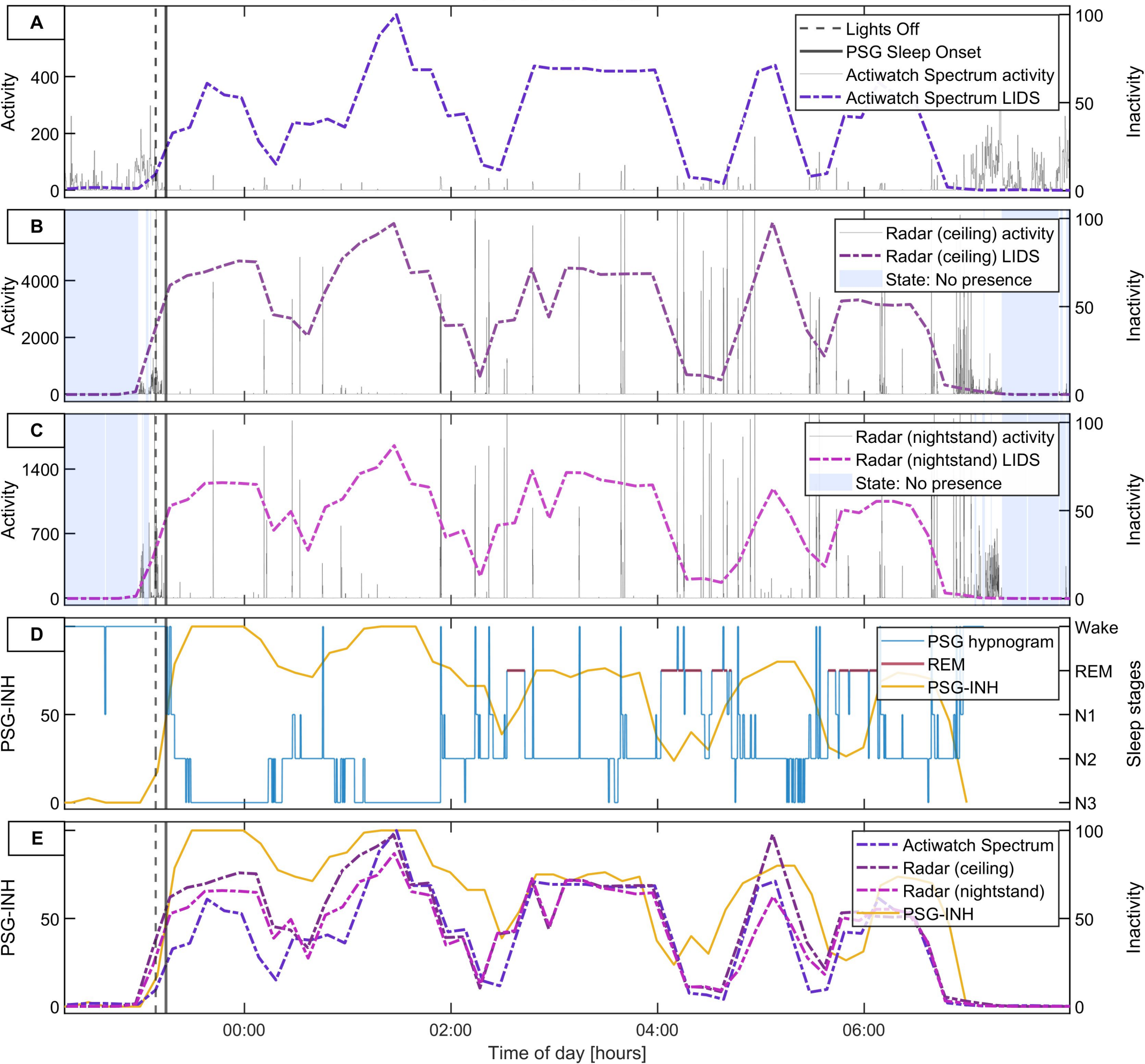

# PID01-20171003

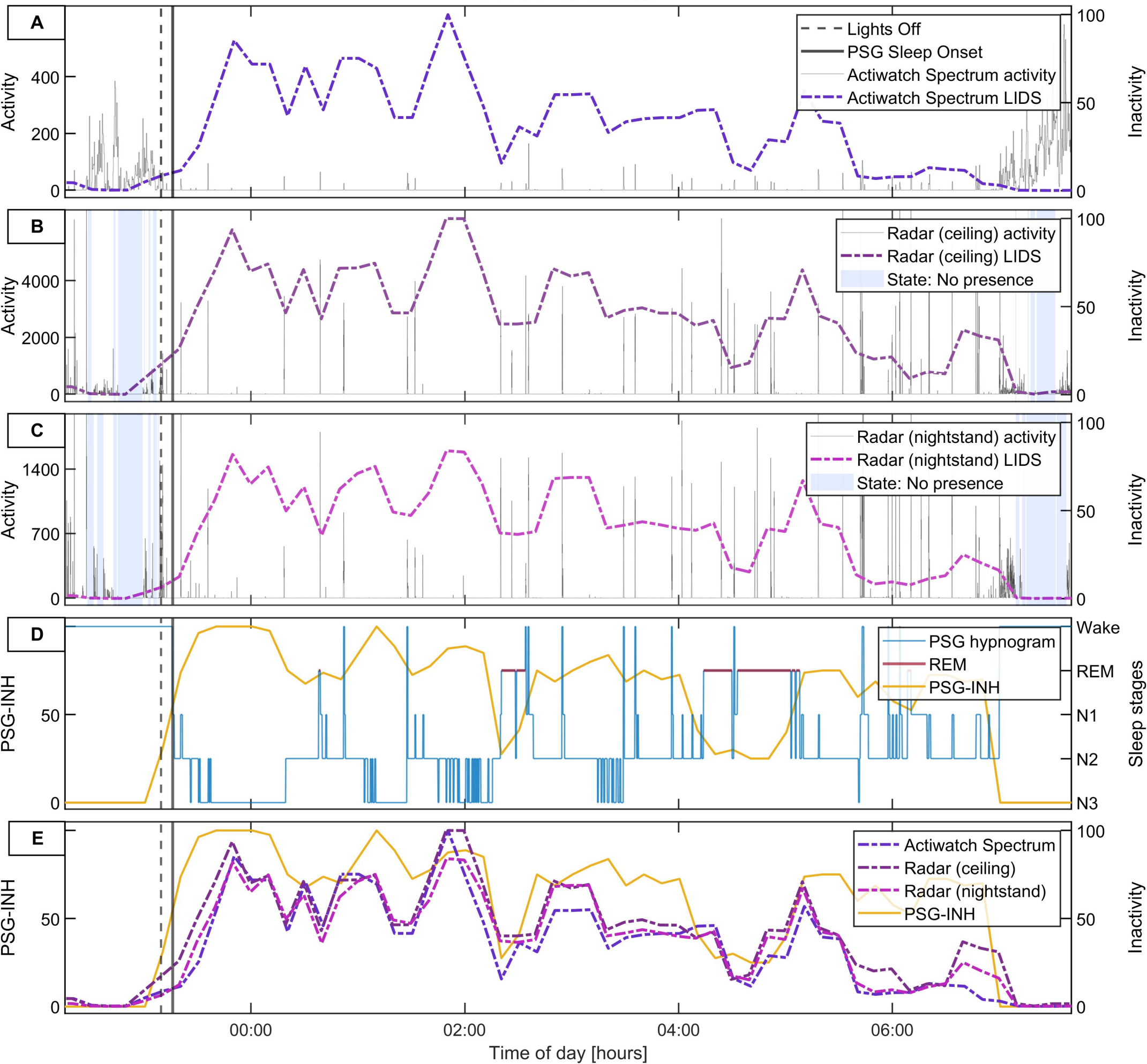

PID02-20170926

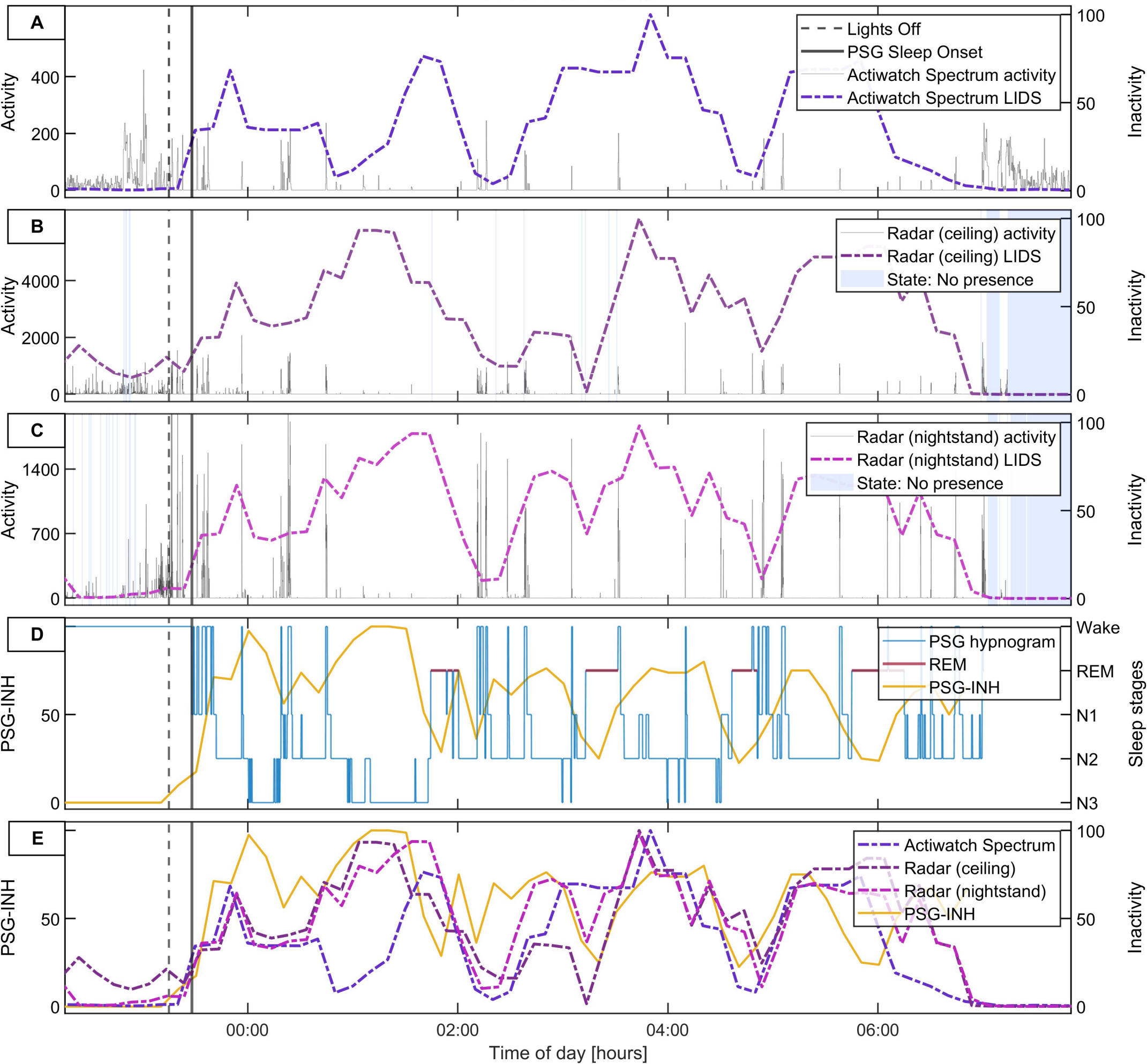

# PID02-20170927

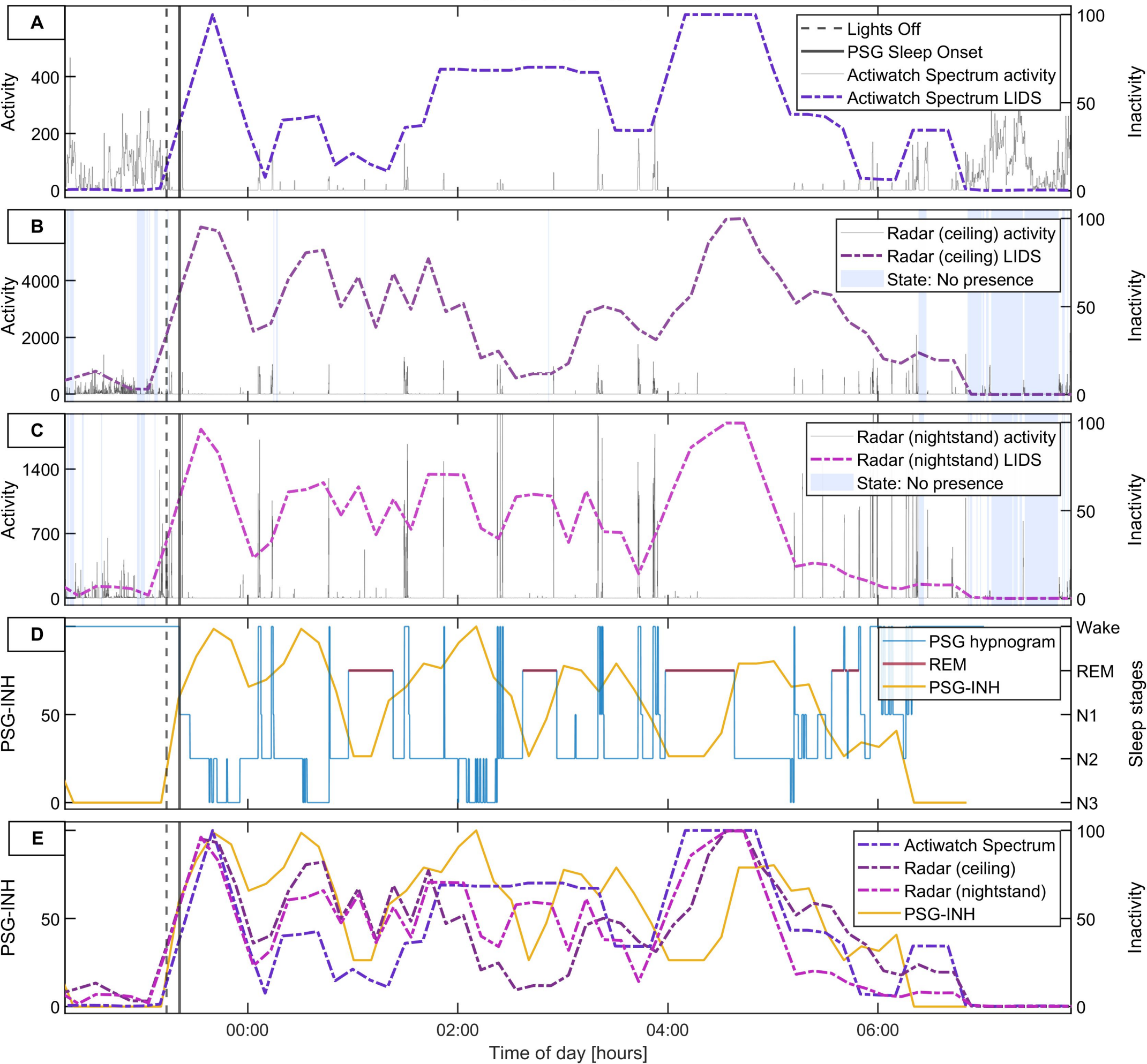

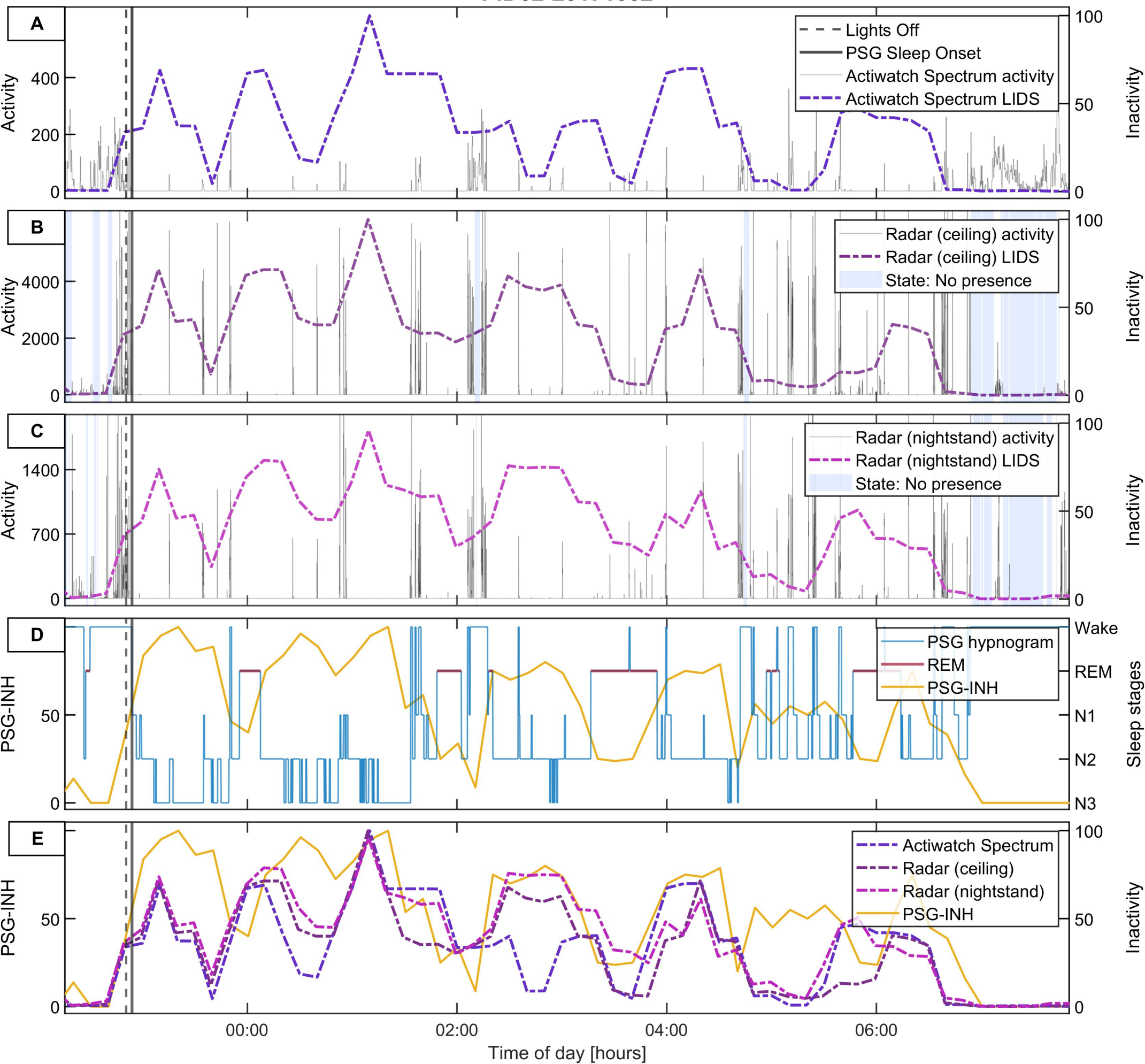

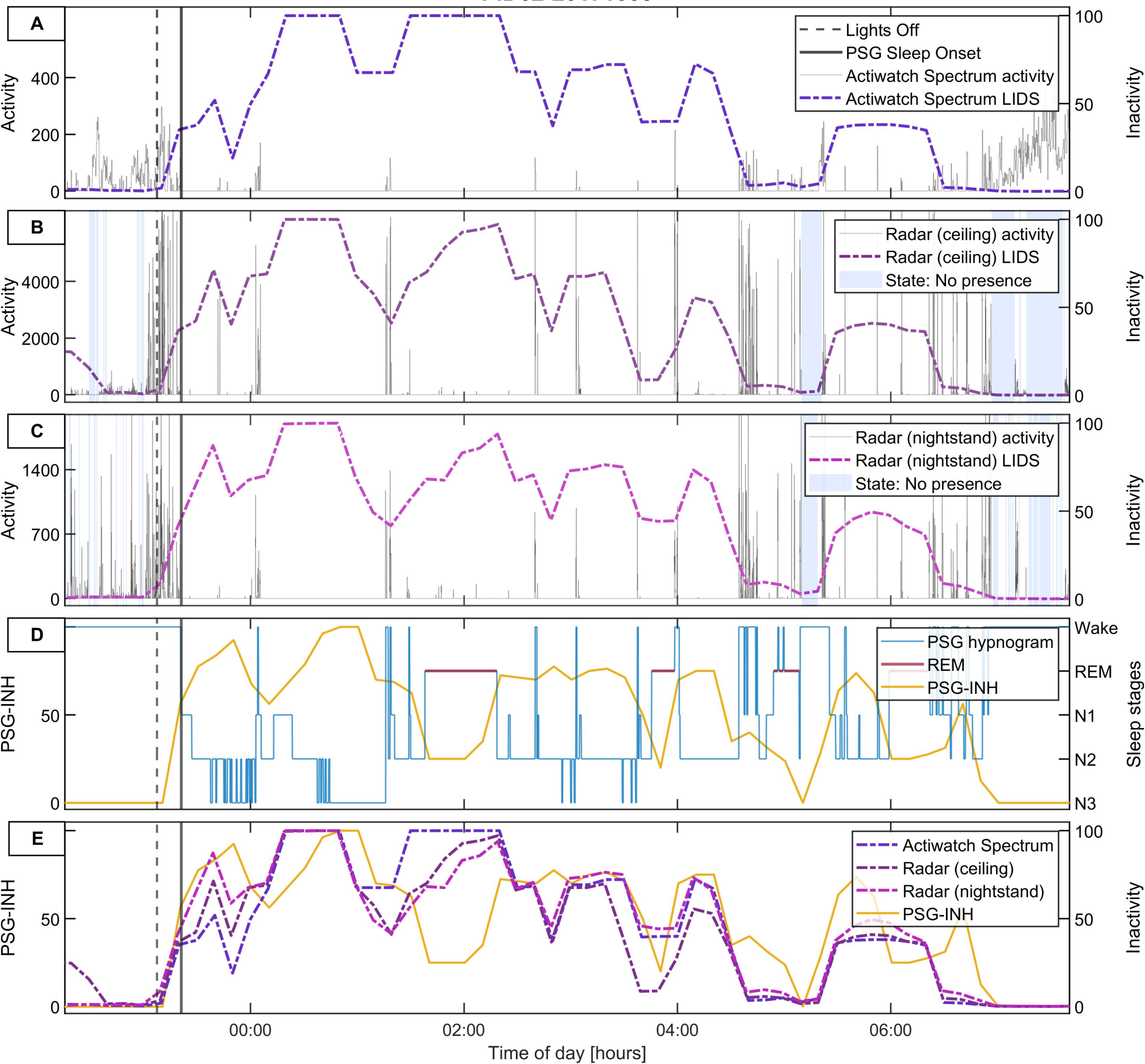

PID03-20170926

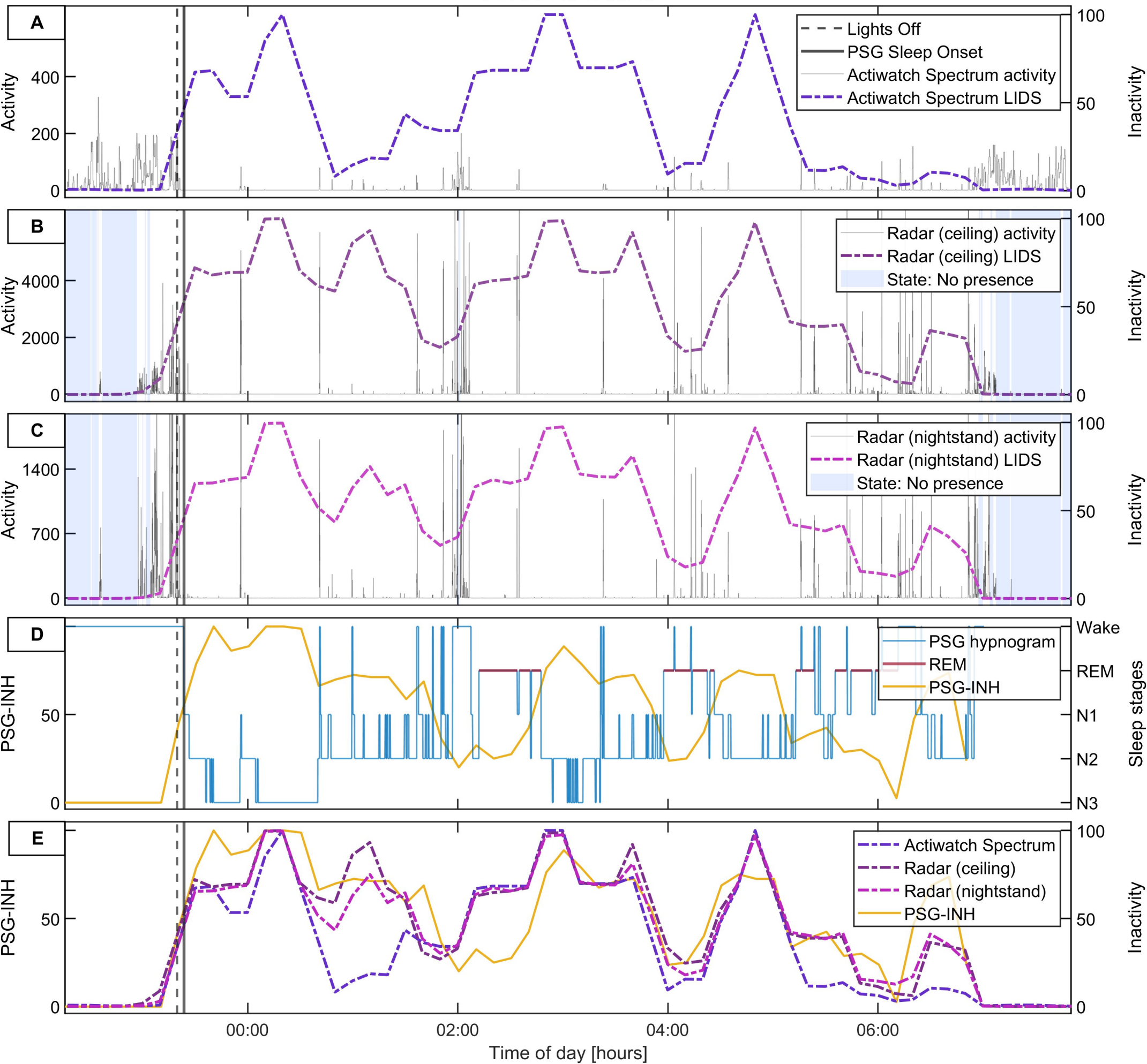

PID03-20170927

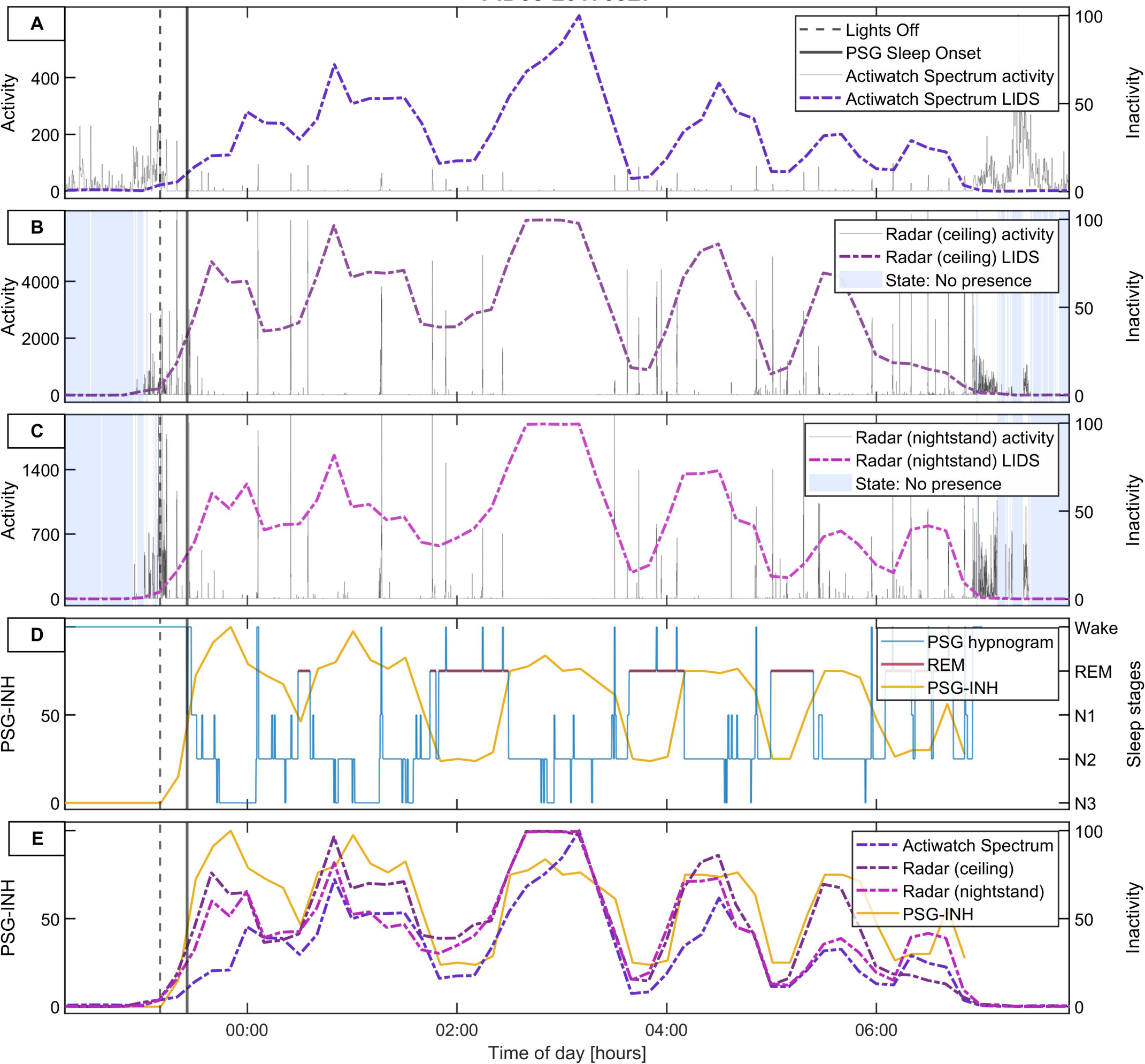

# PID03-20171002

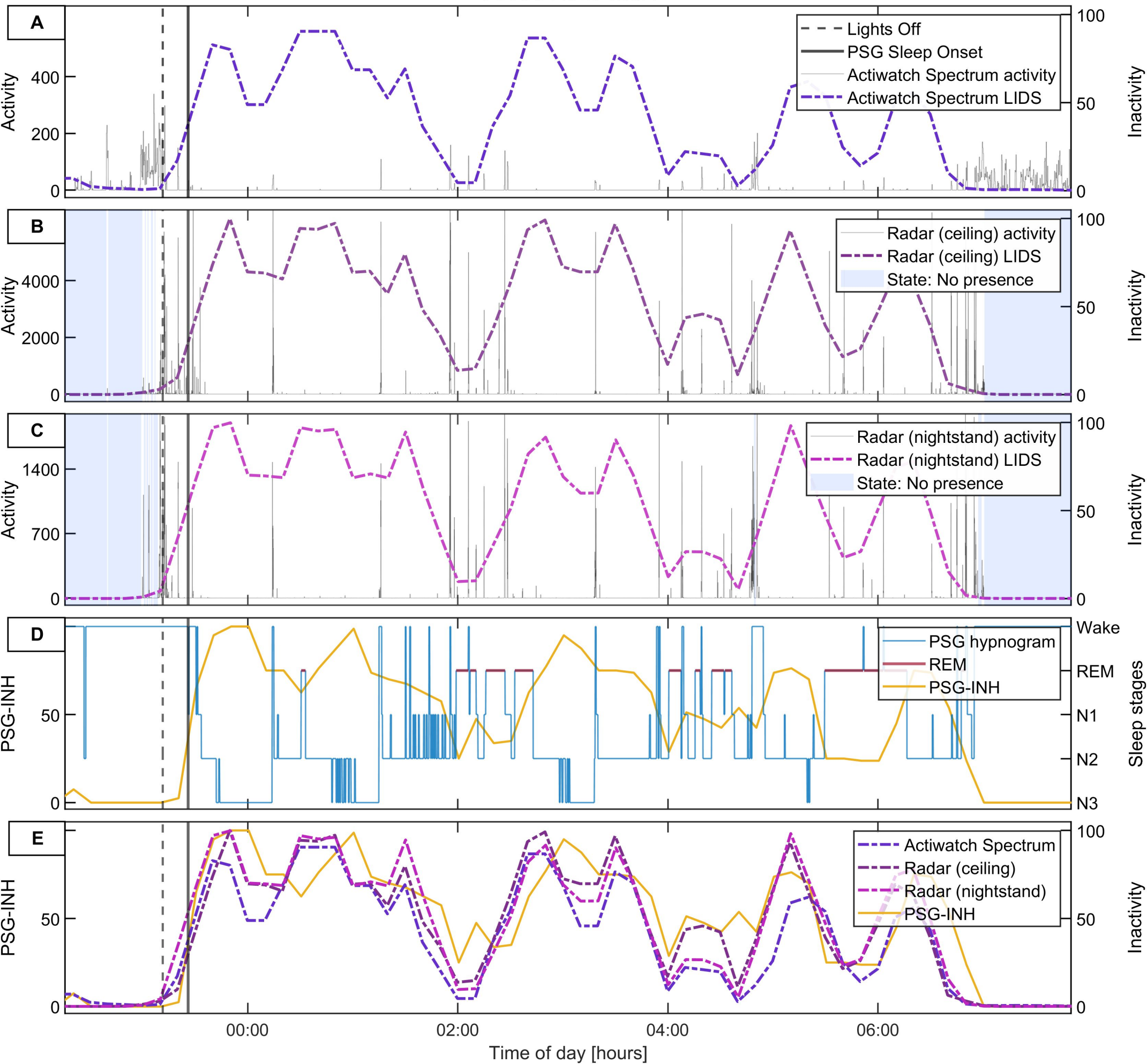

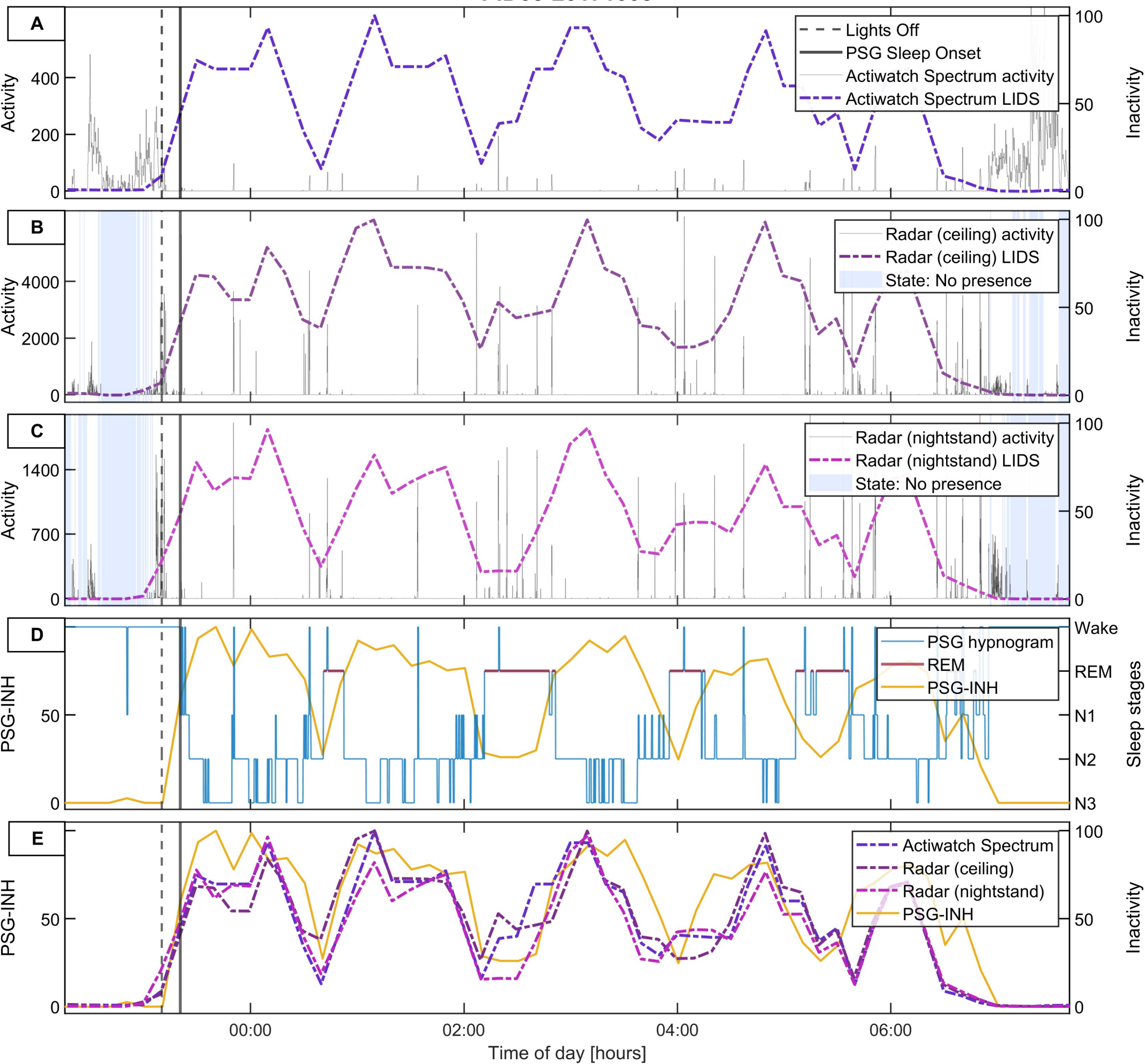

# PID04-20170928

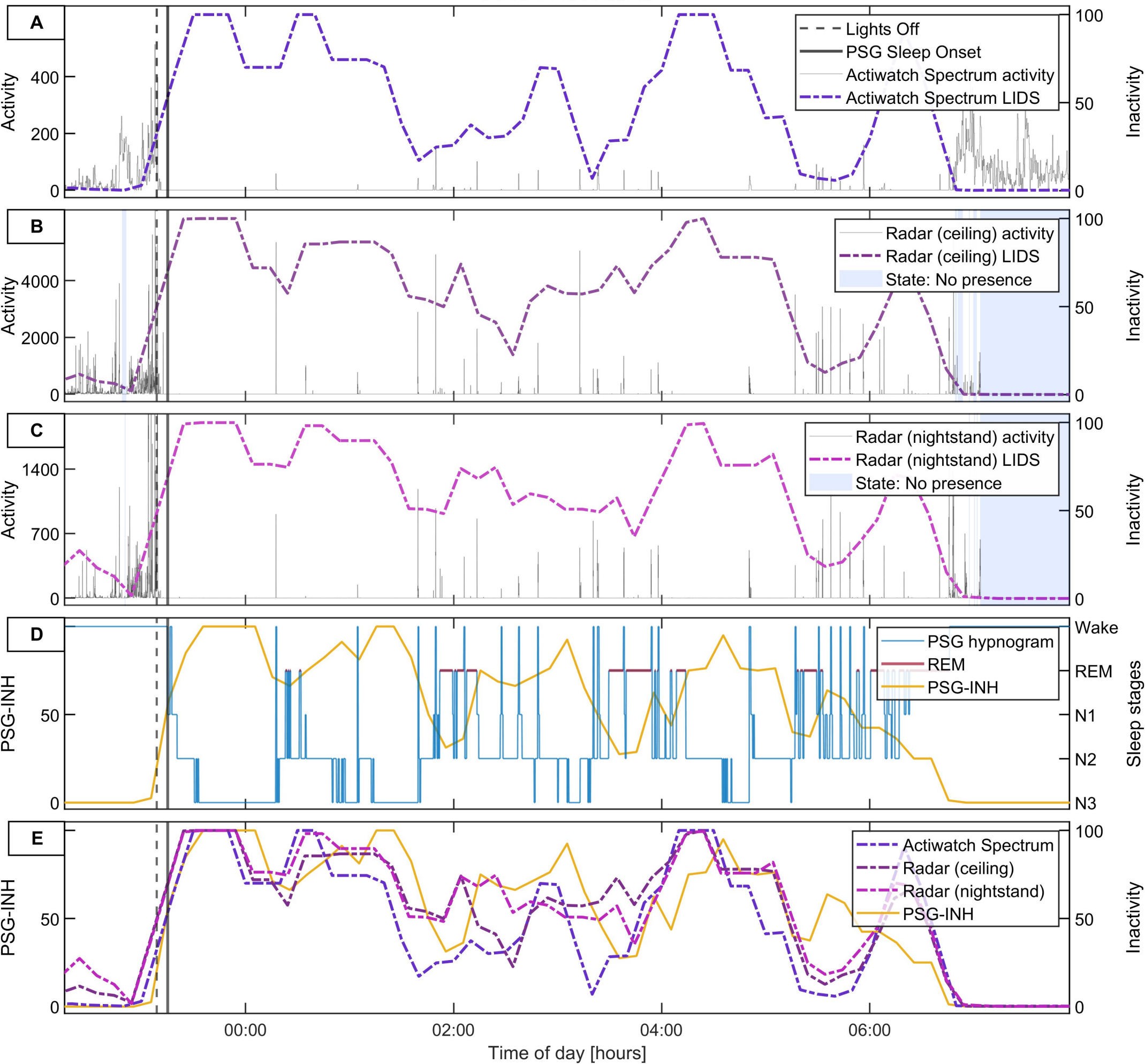

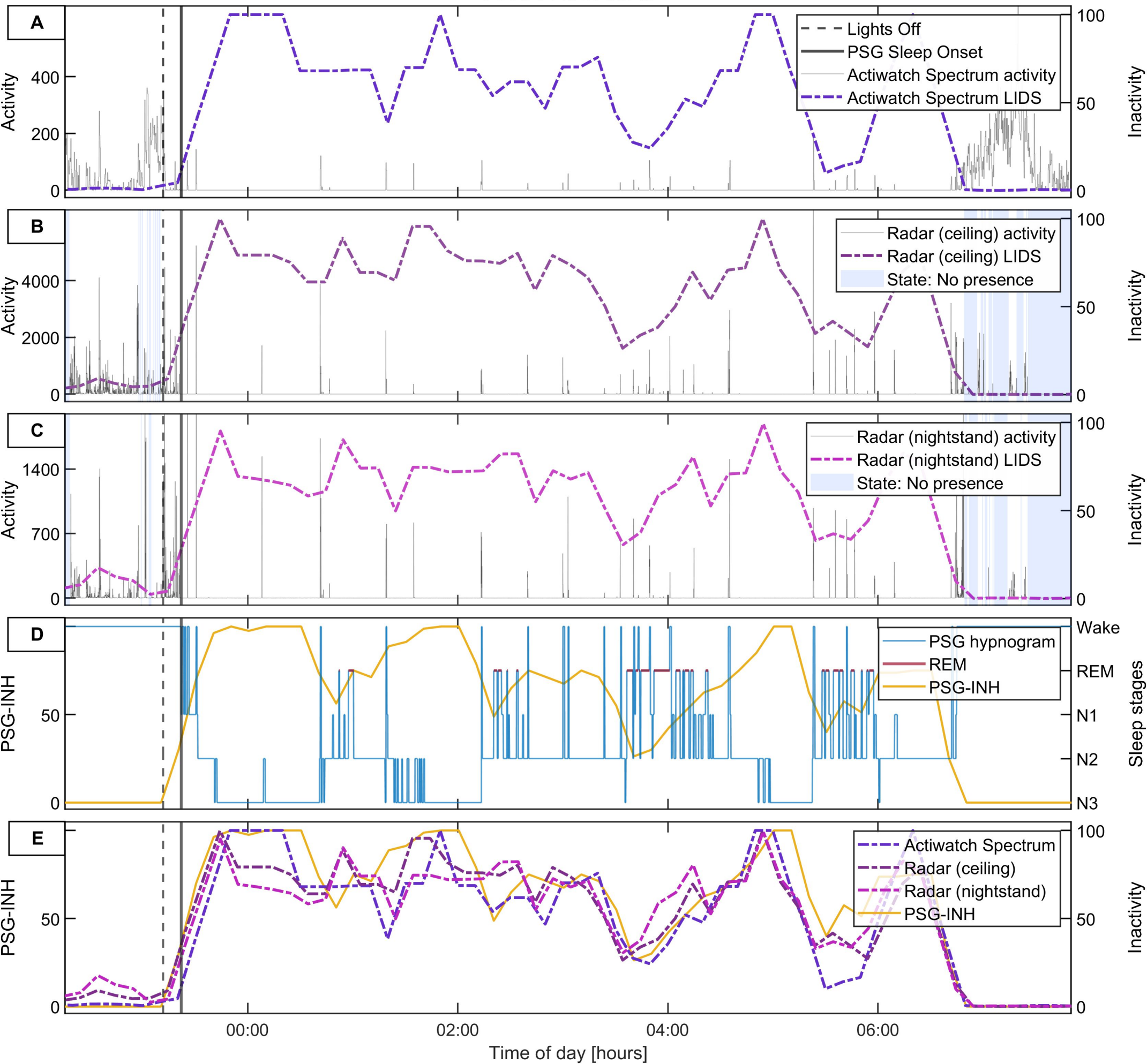

PID04-20171004

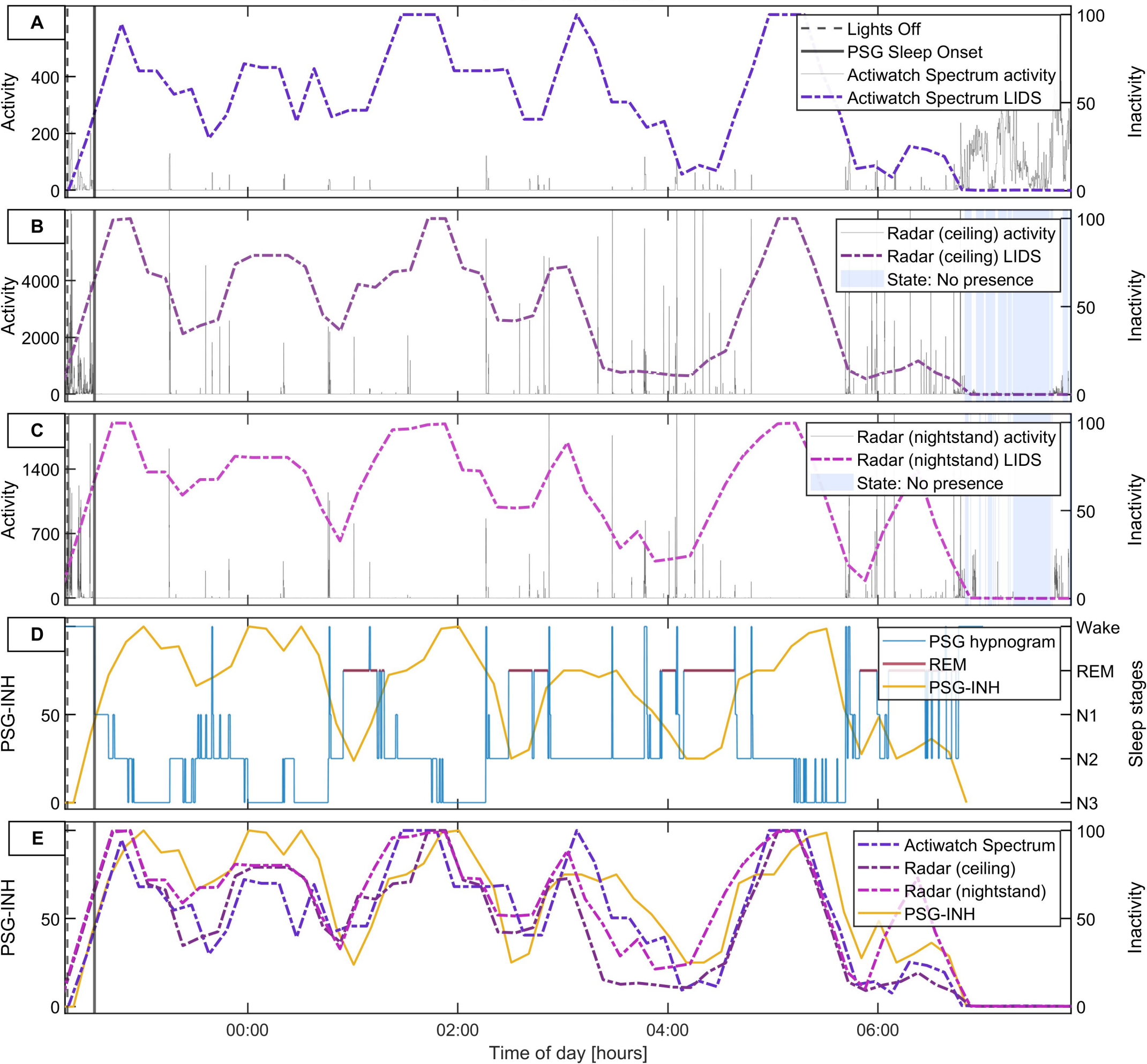

PID04-20171005

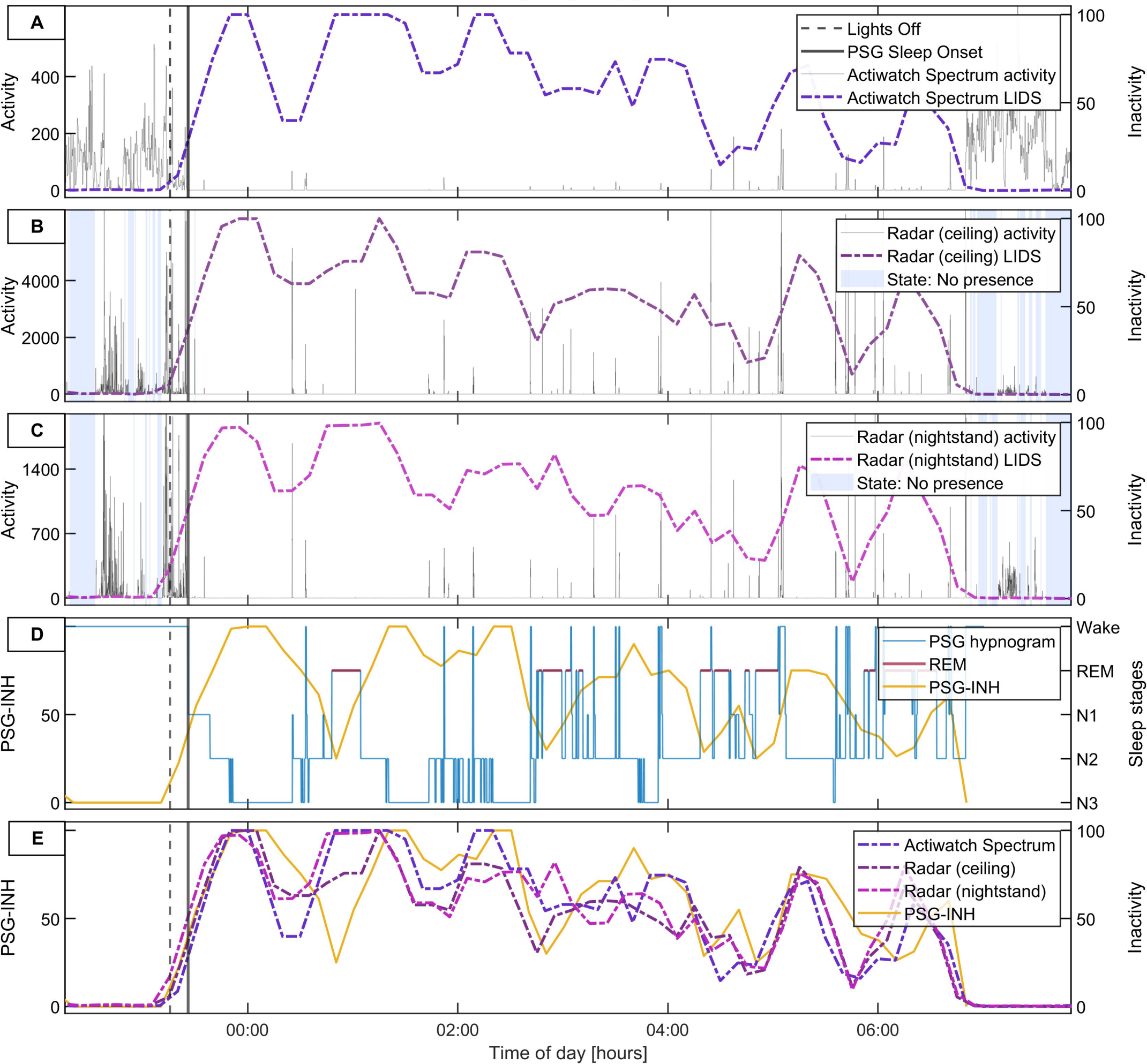

# PID06-20171004

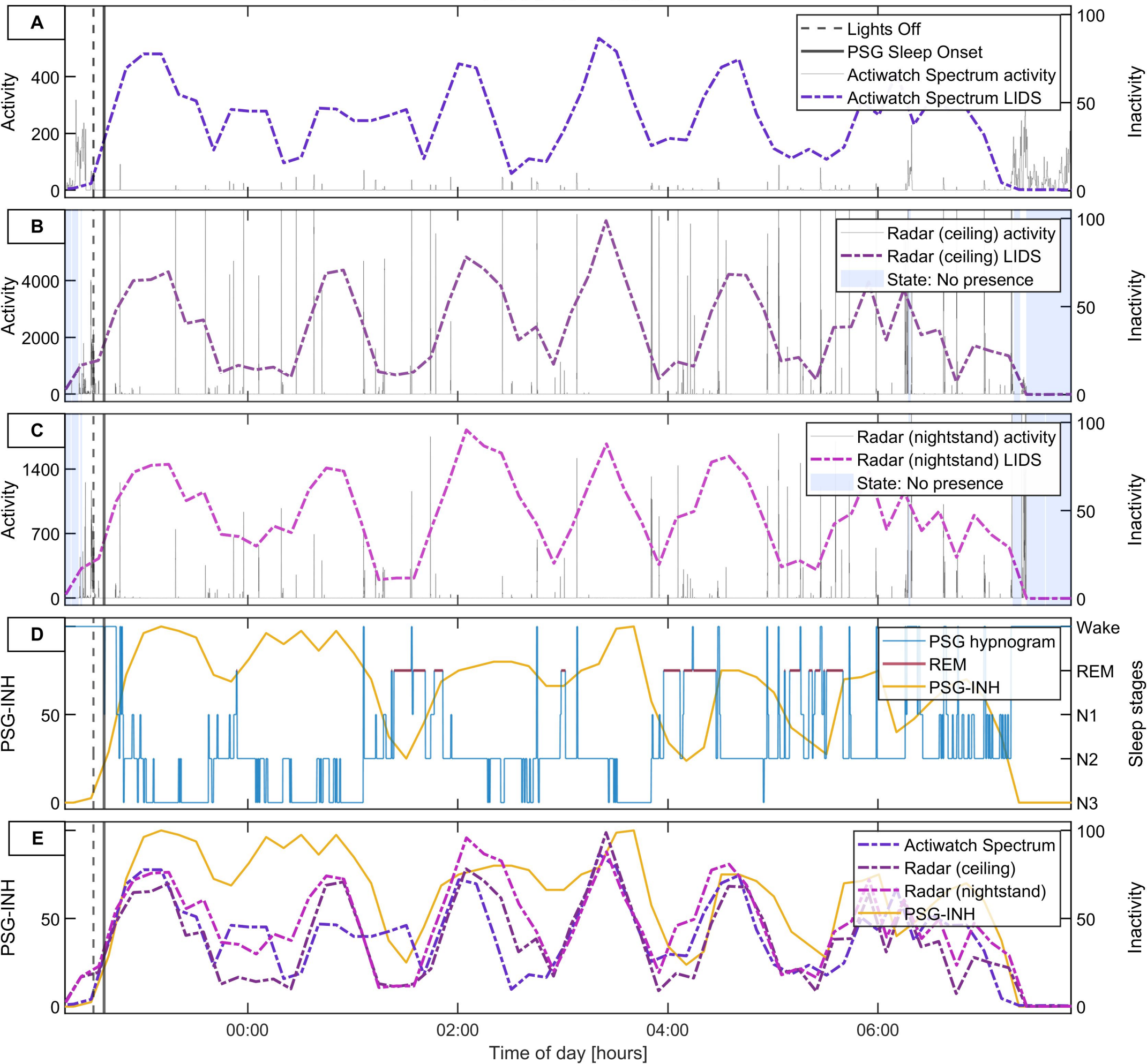

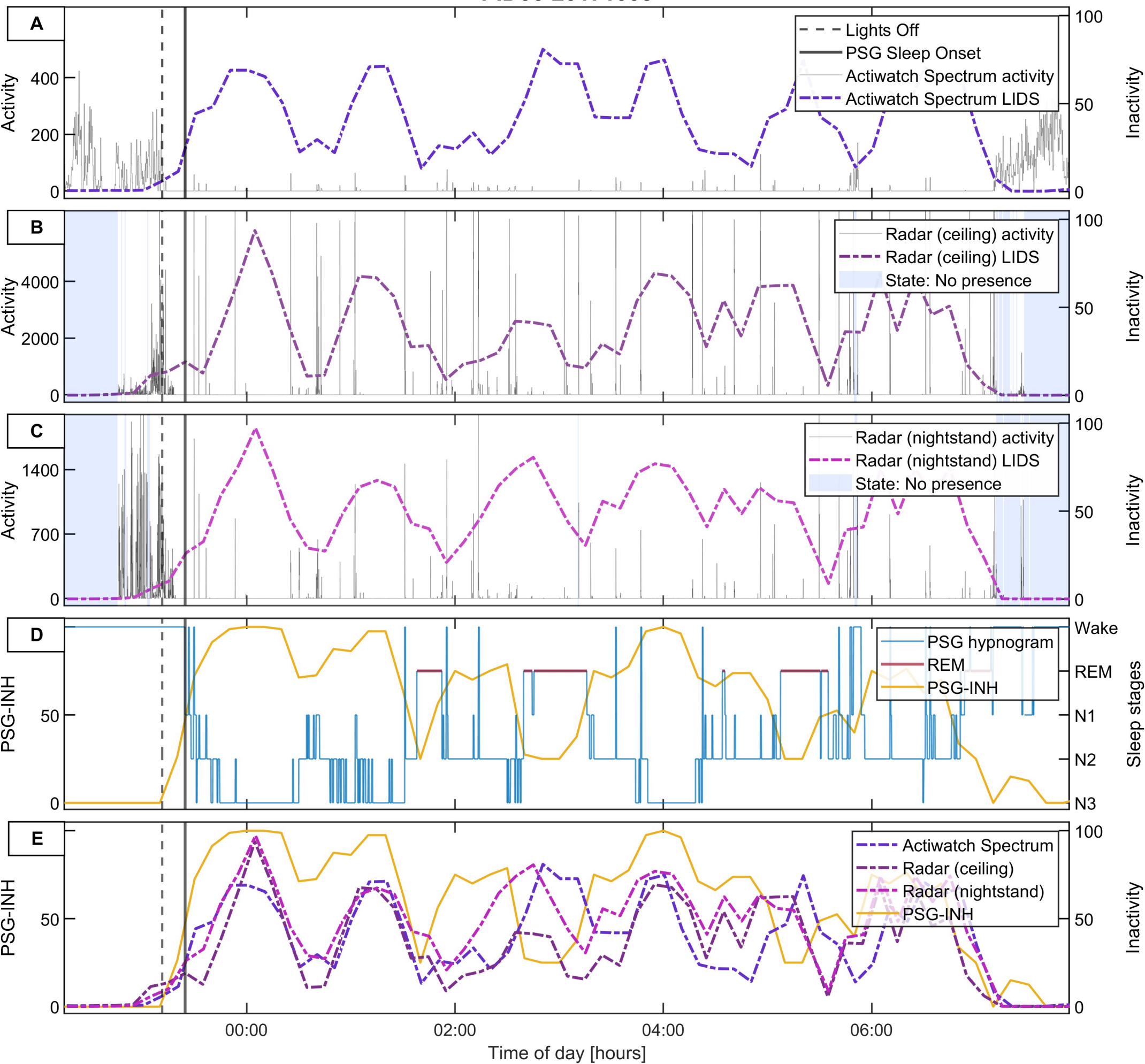

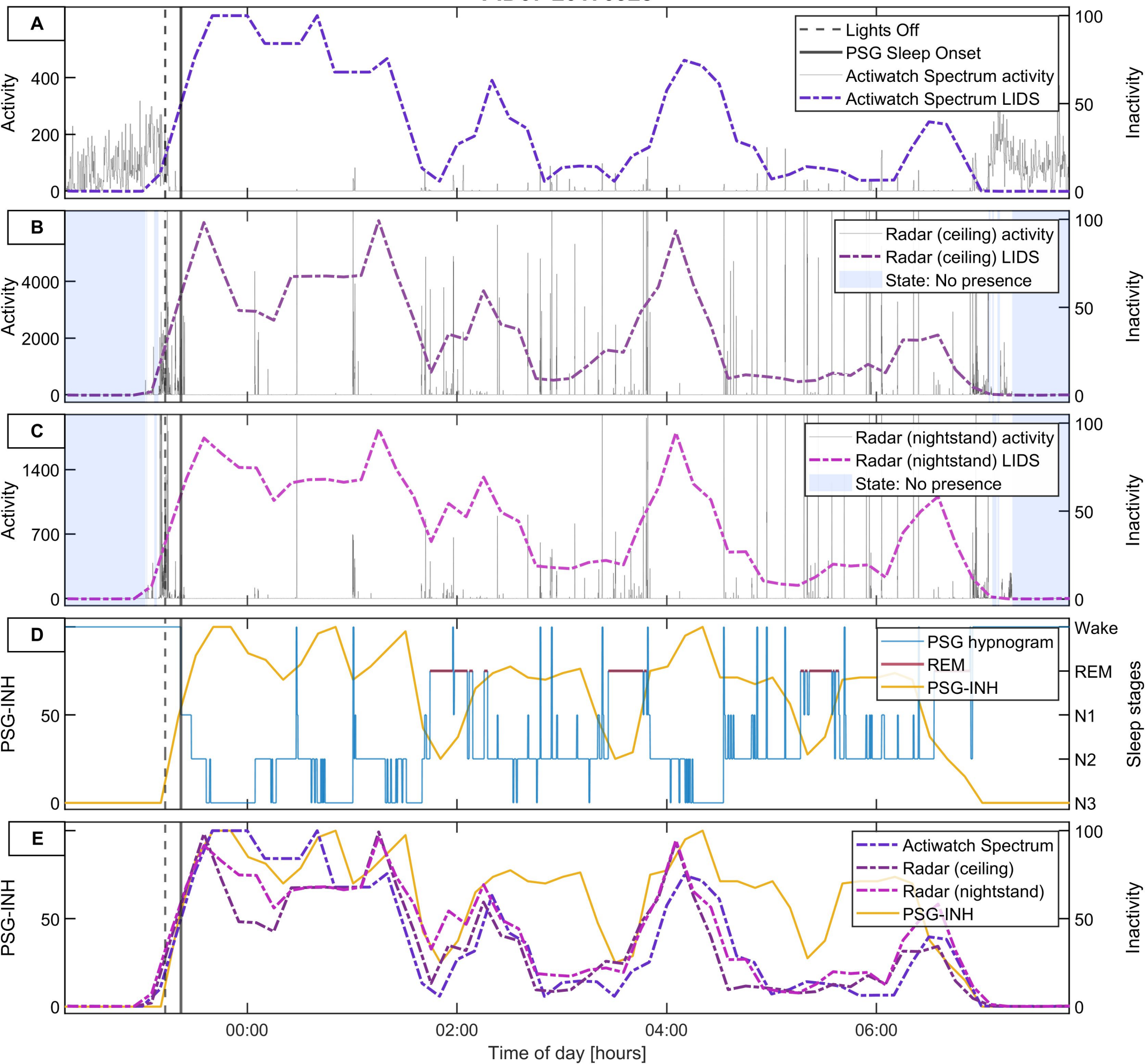

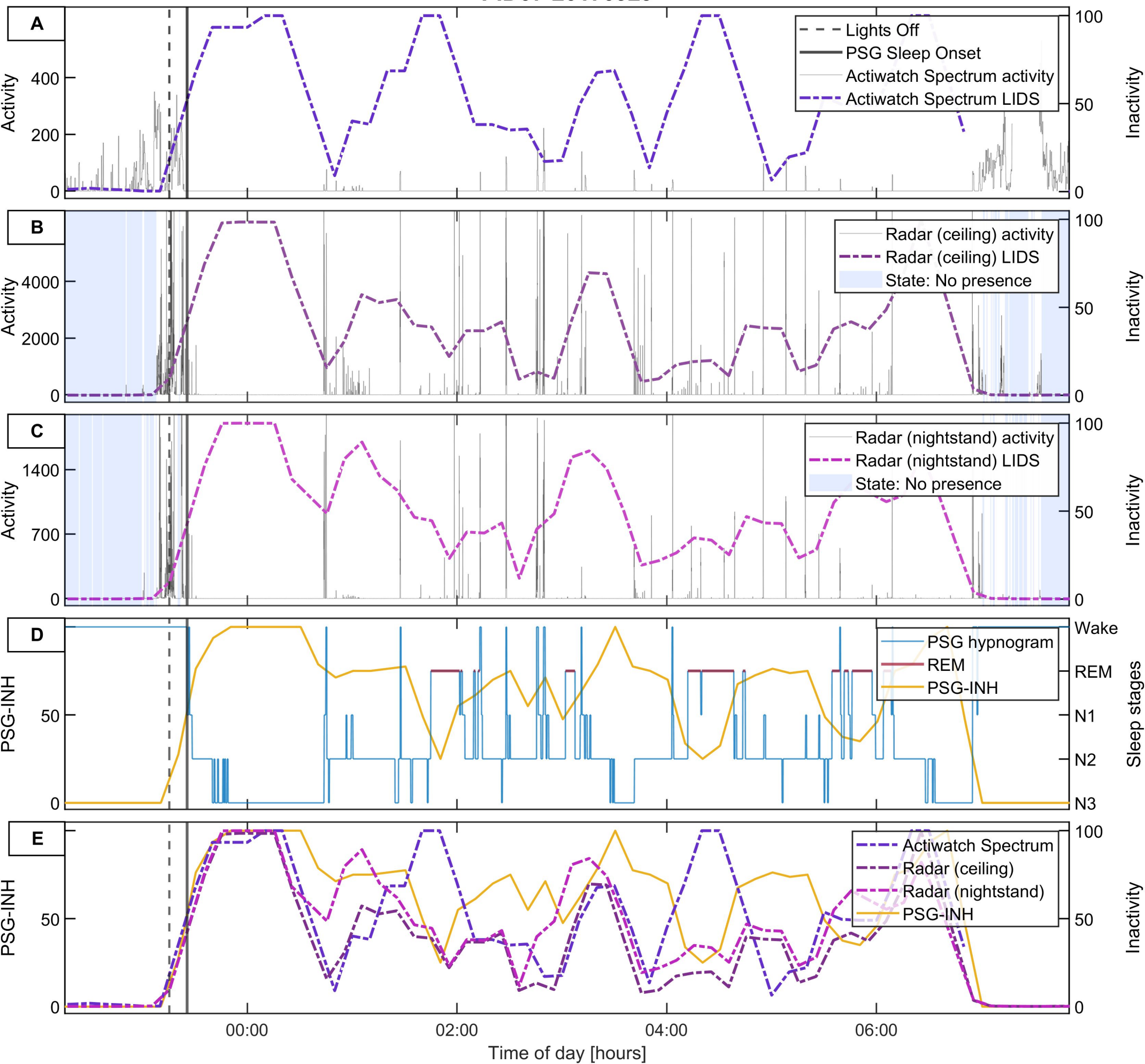

PID08-20170928

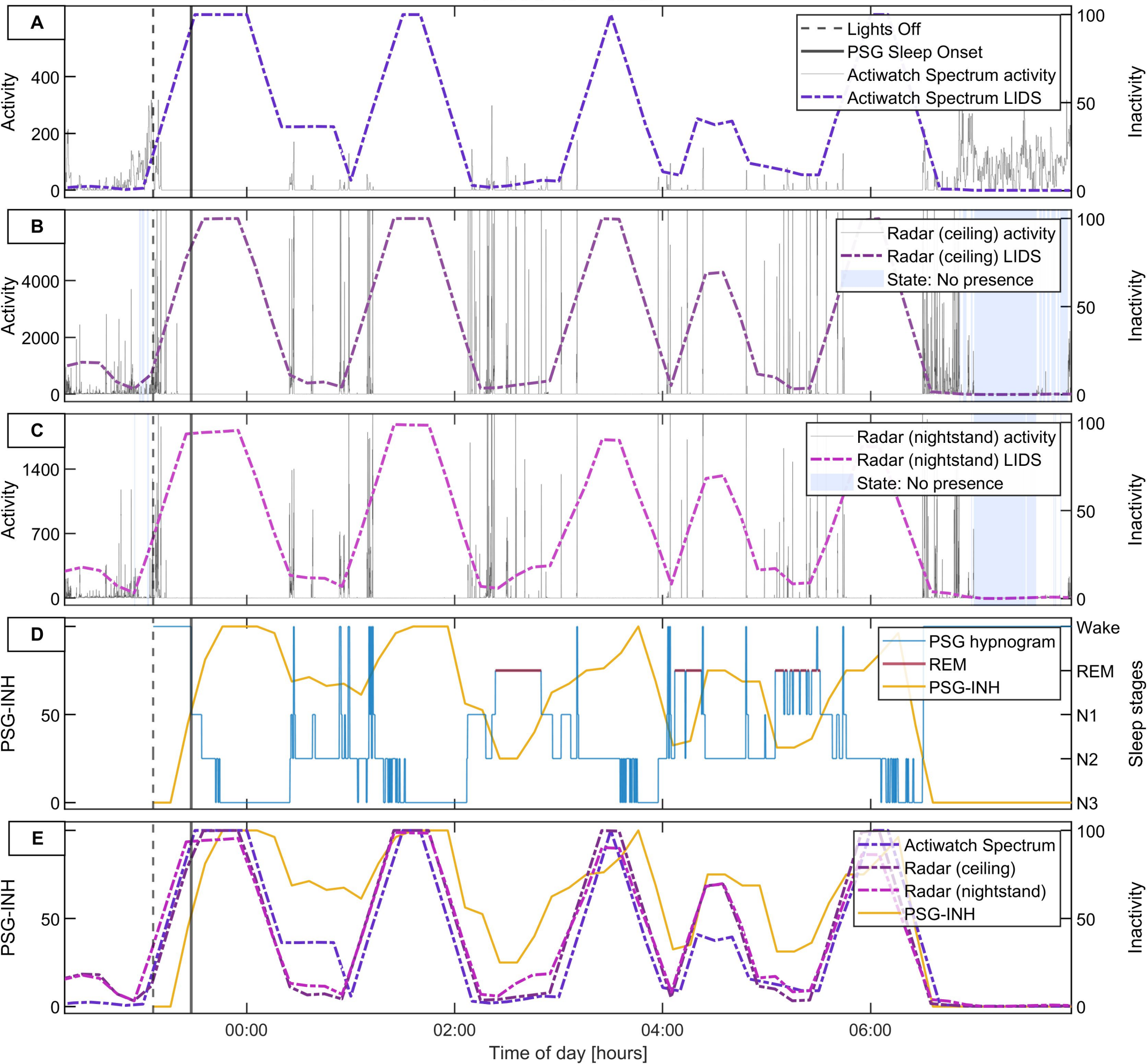

PID08-20170929

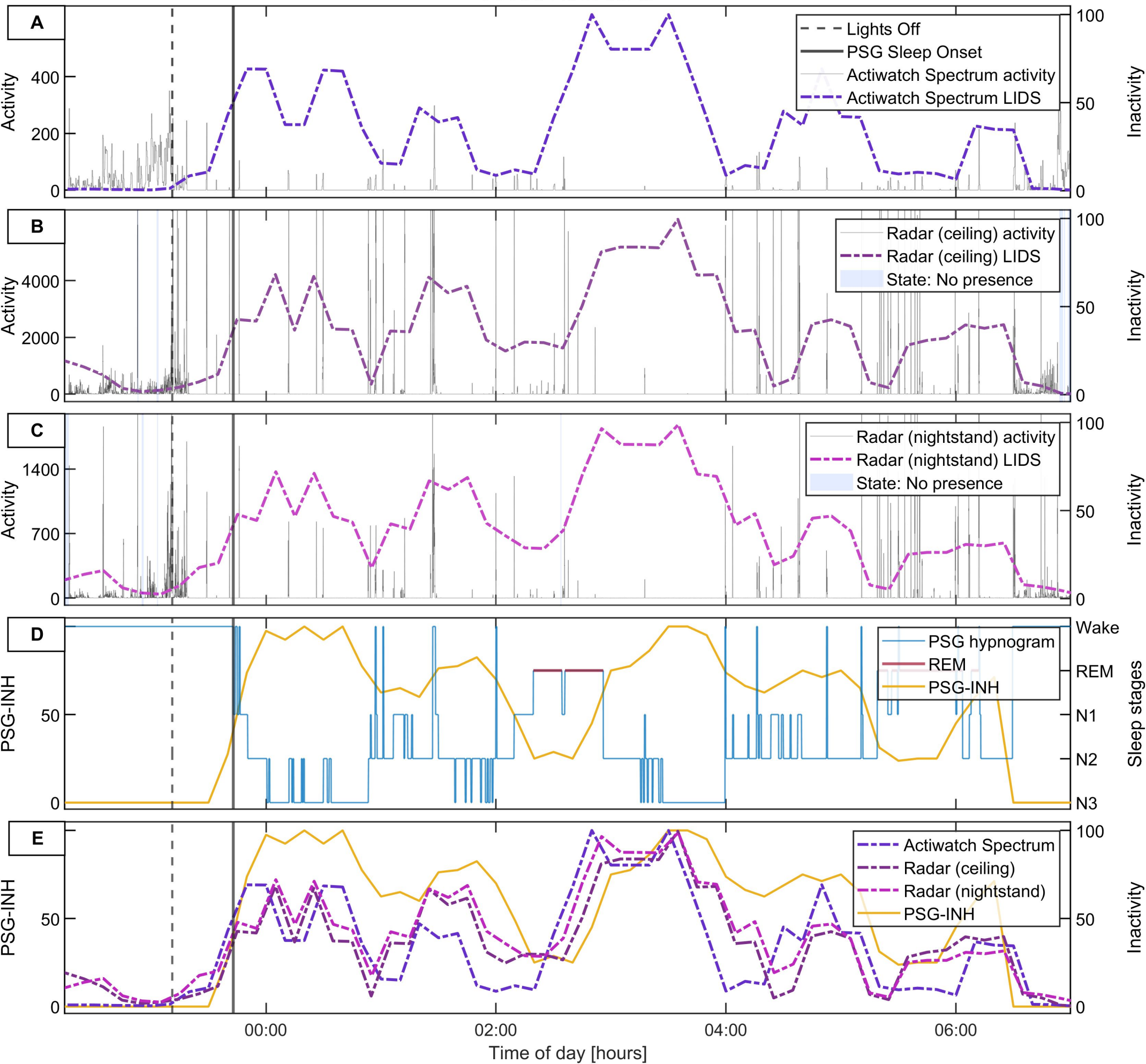

PID08-20171004

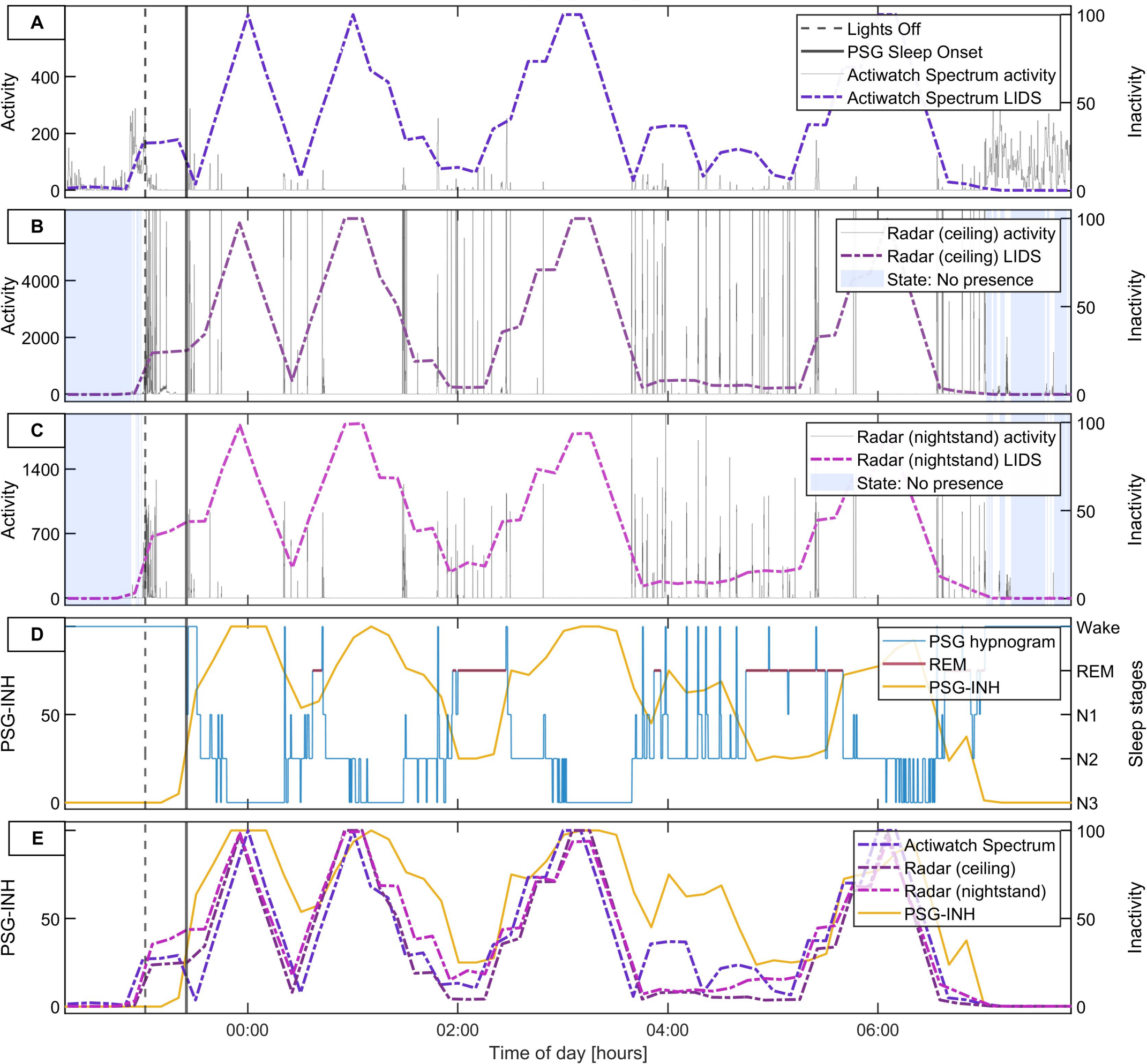

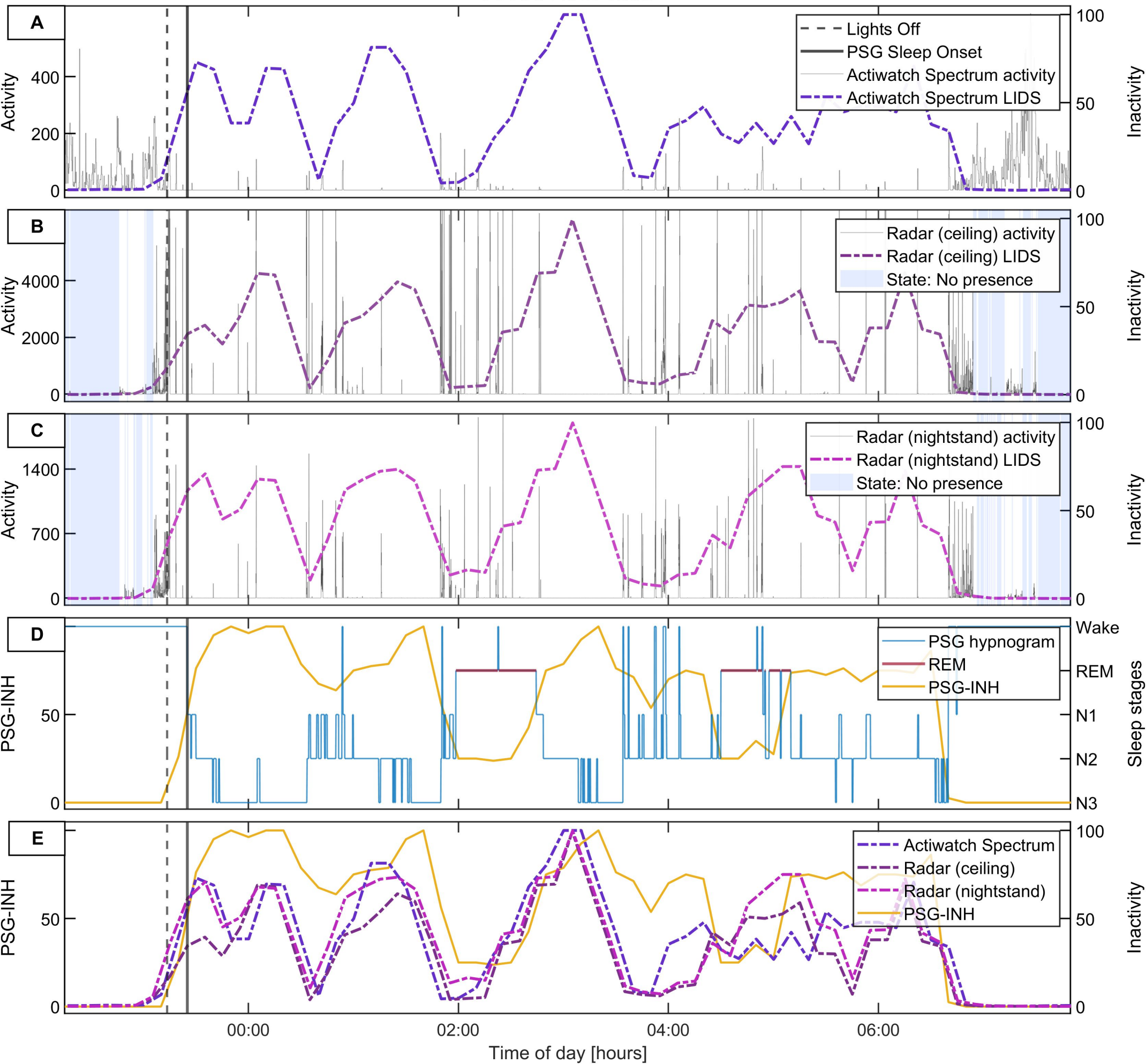

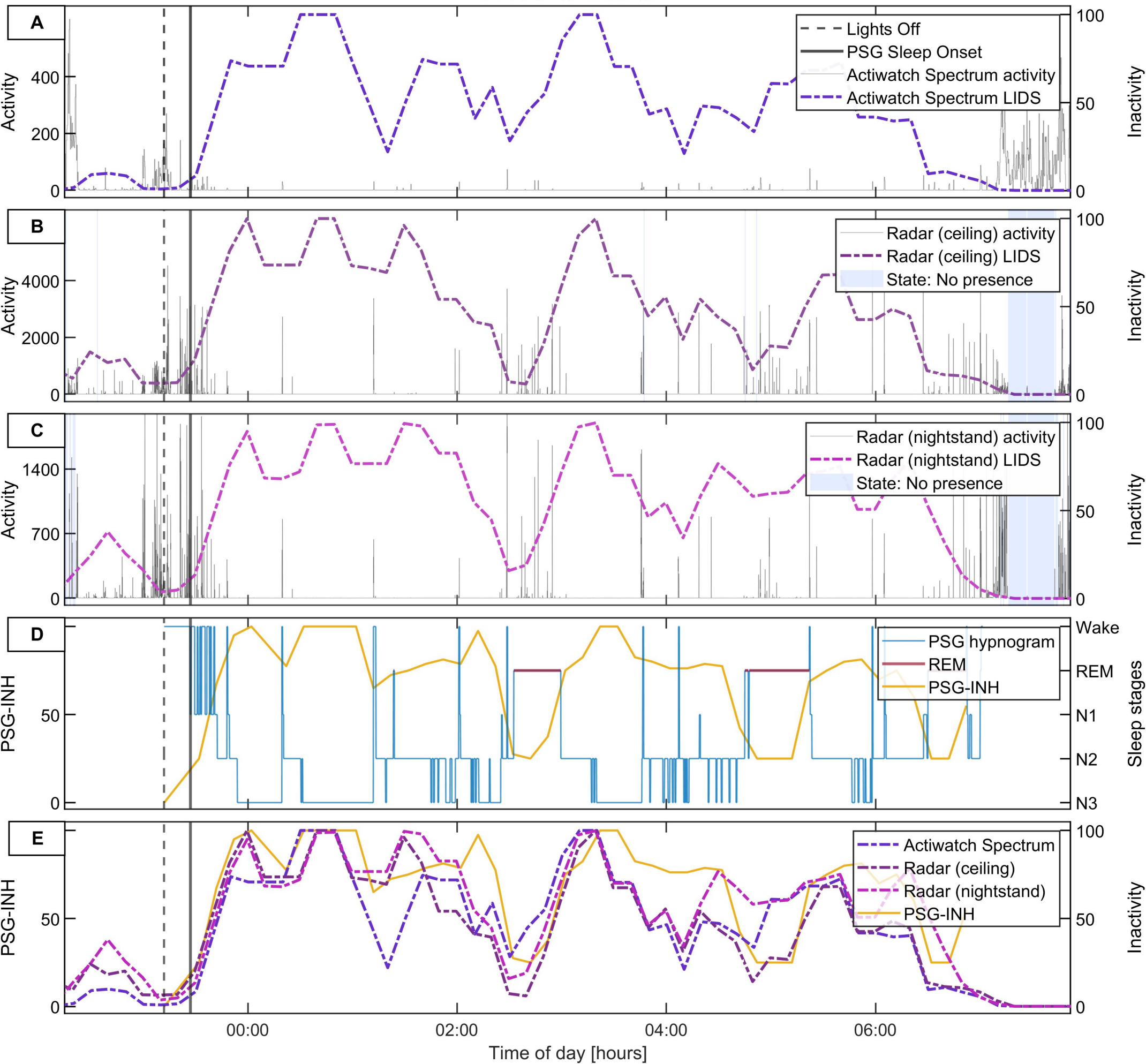

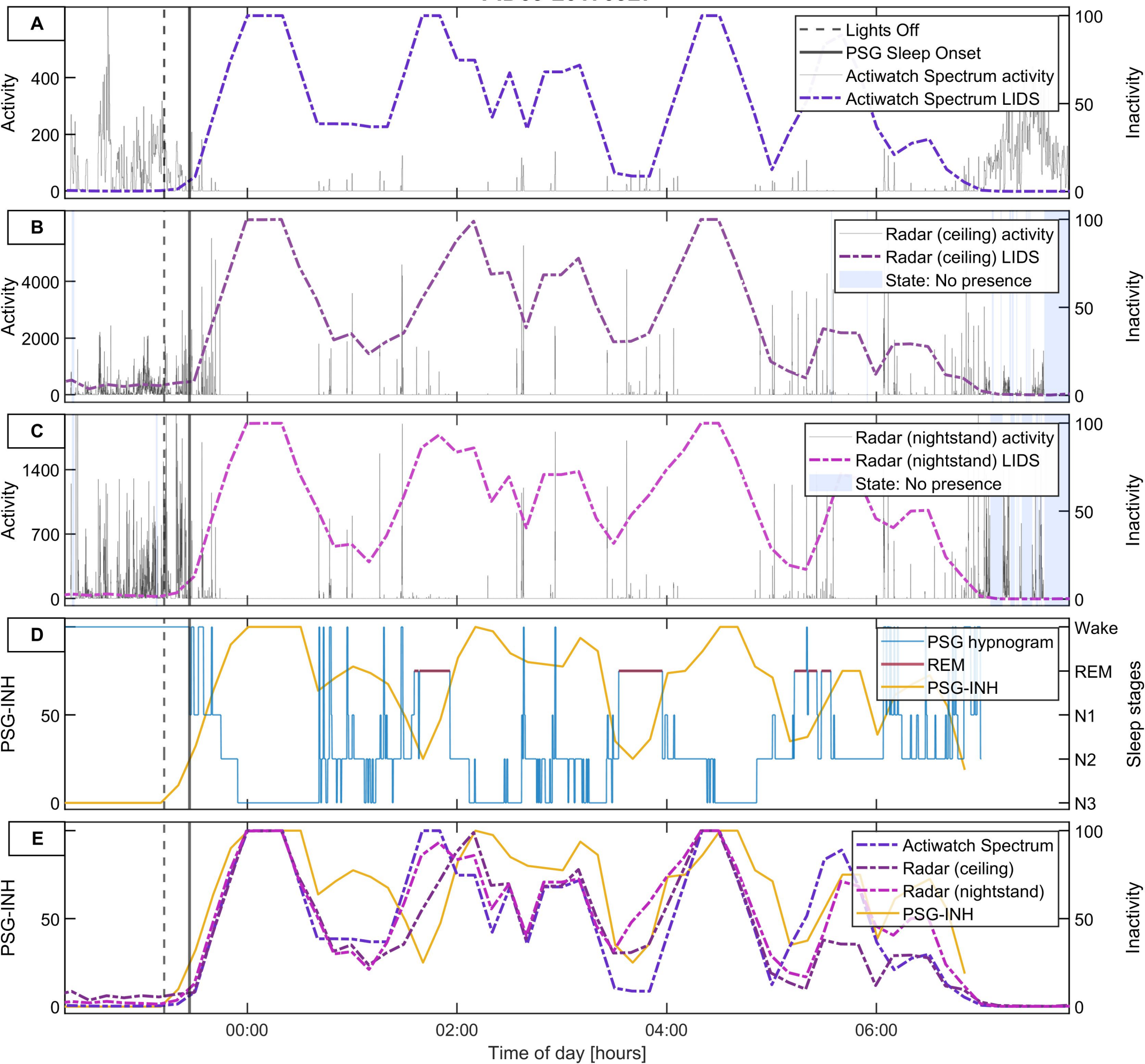

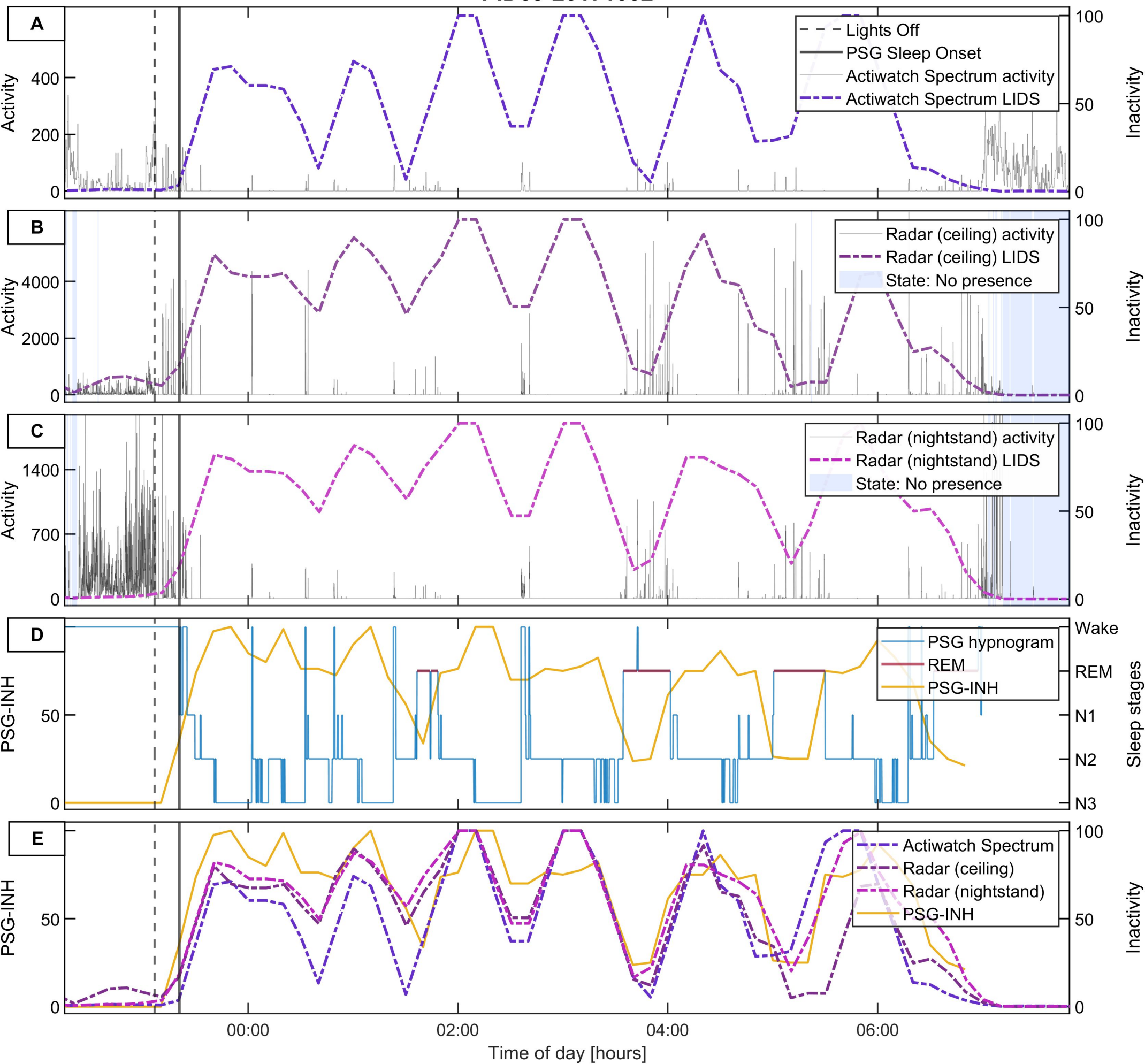

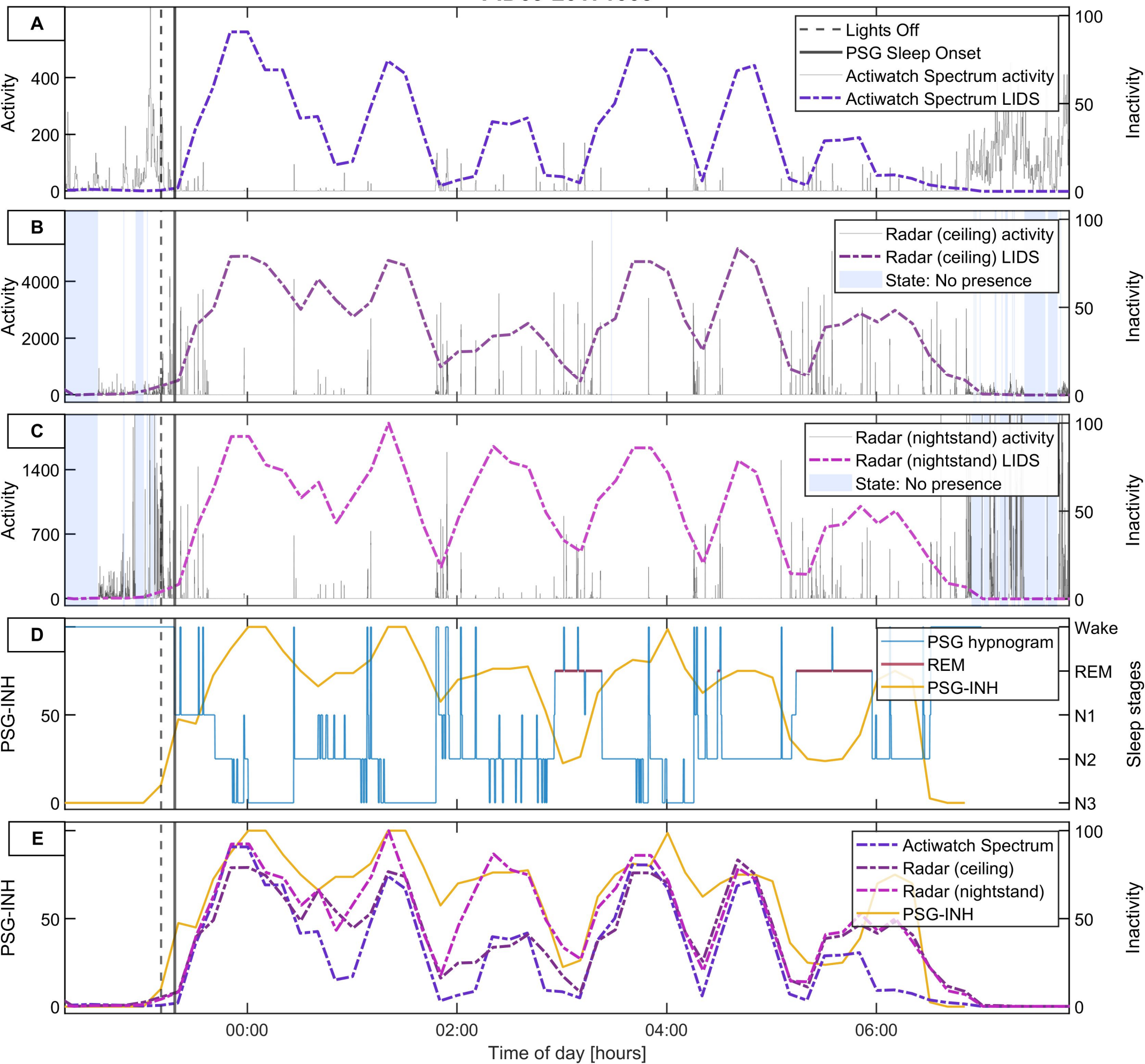

PID10-20170926

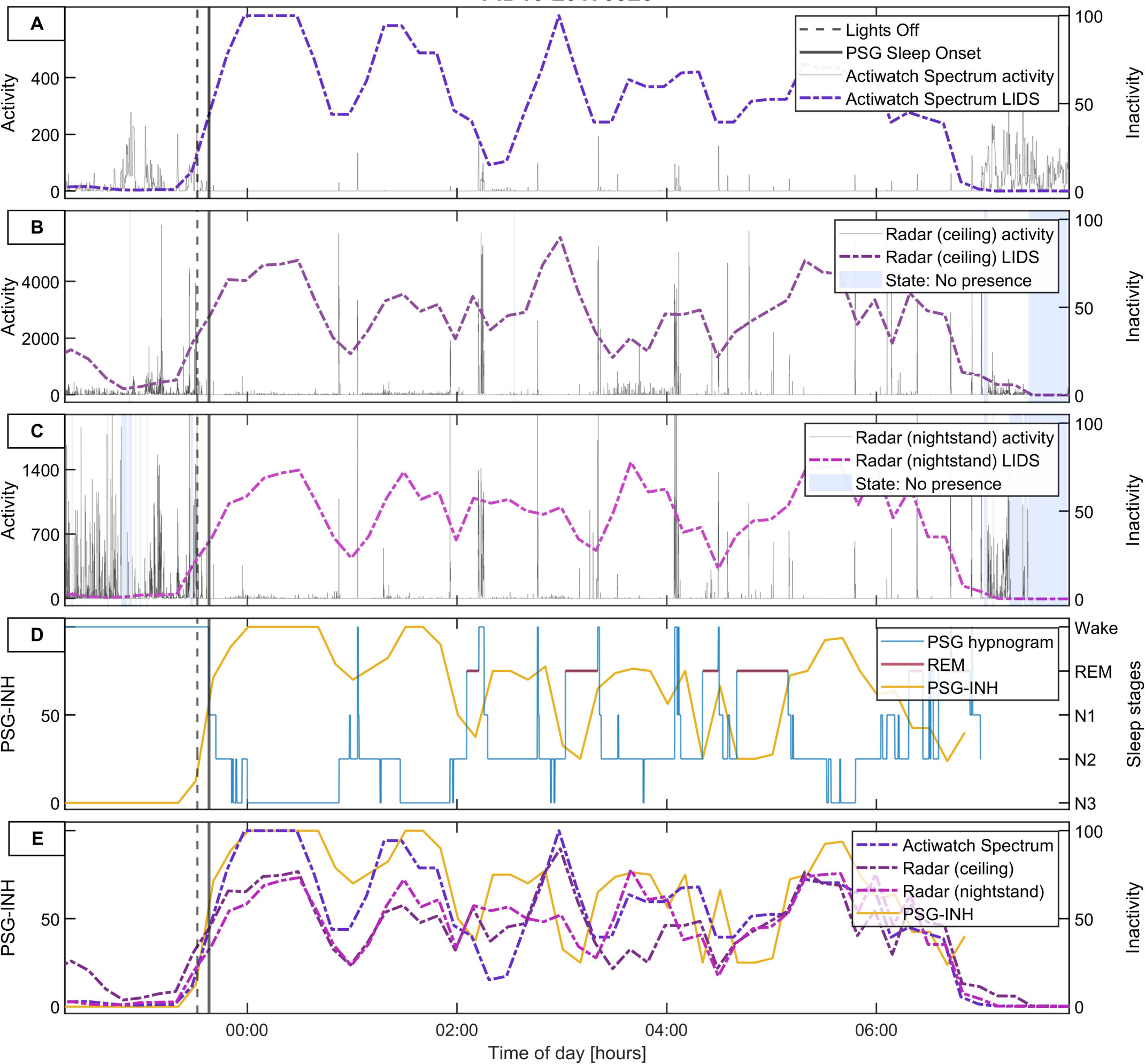

PID10-20170927

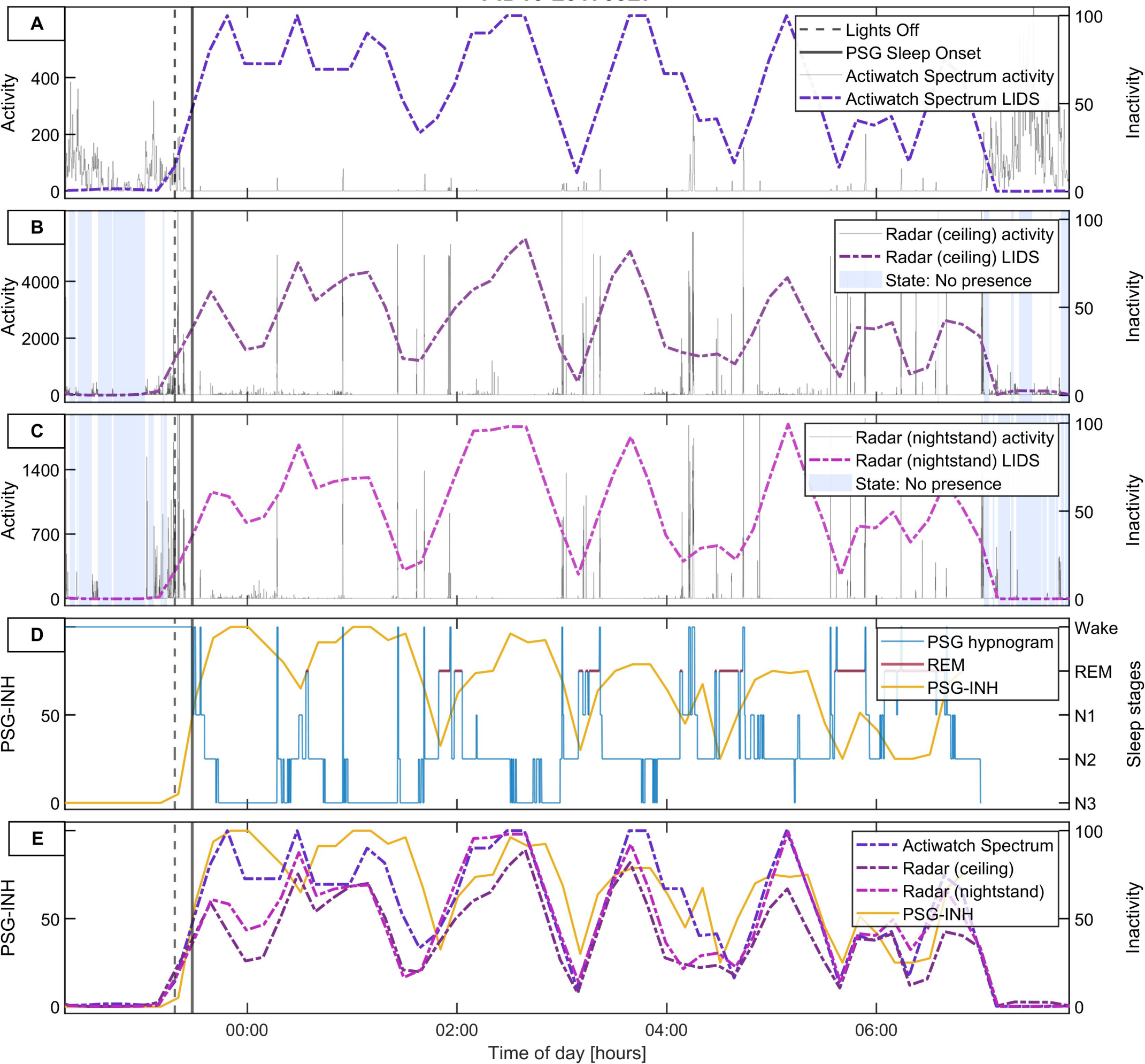

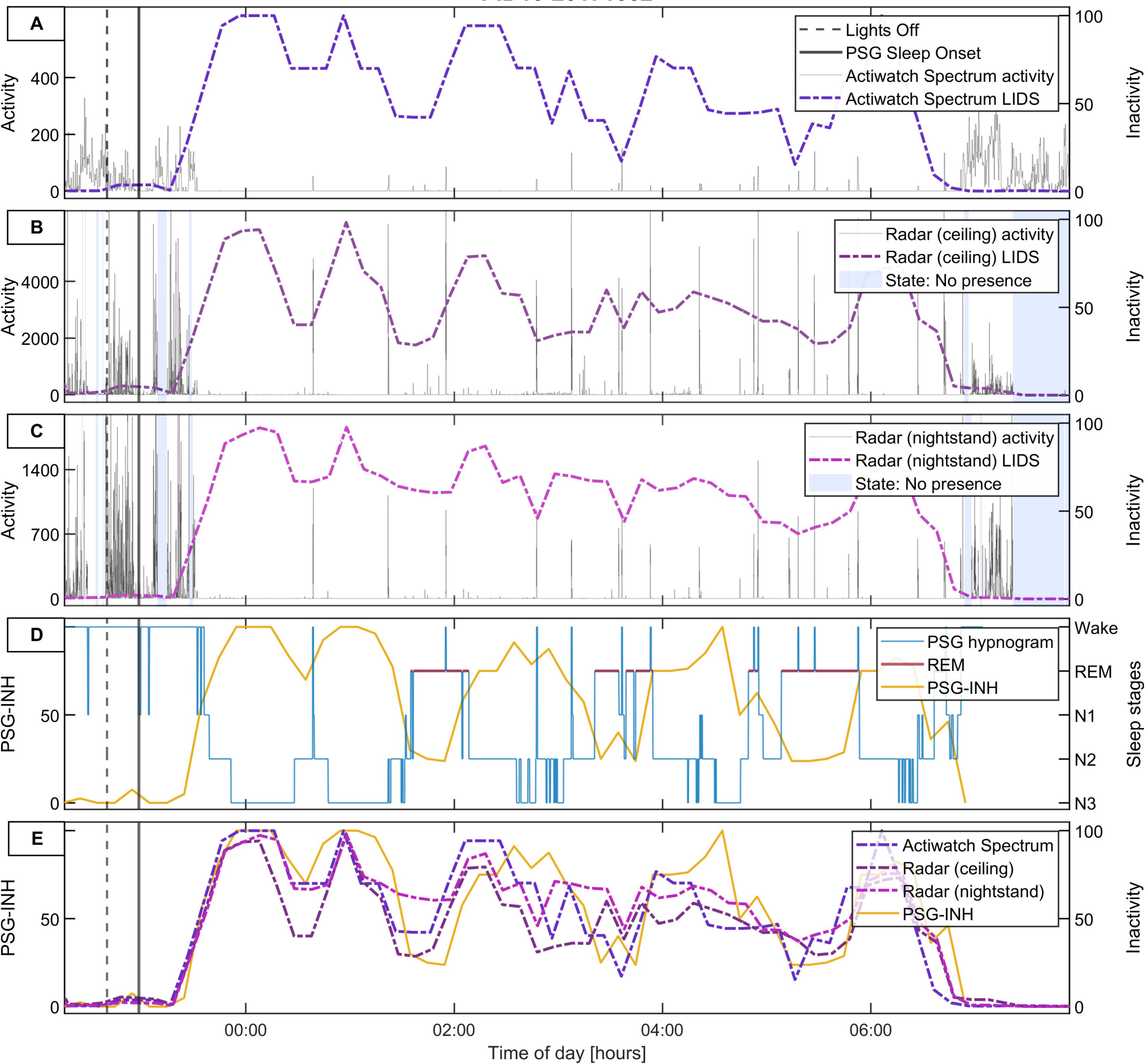

# PID10-20171003

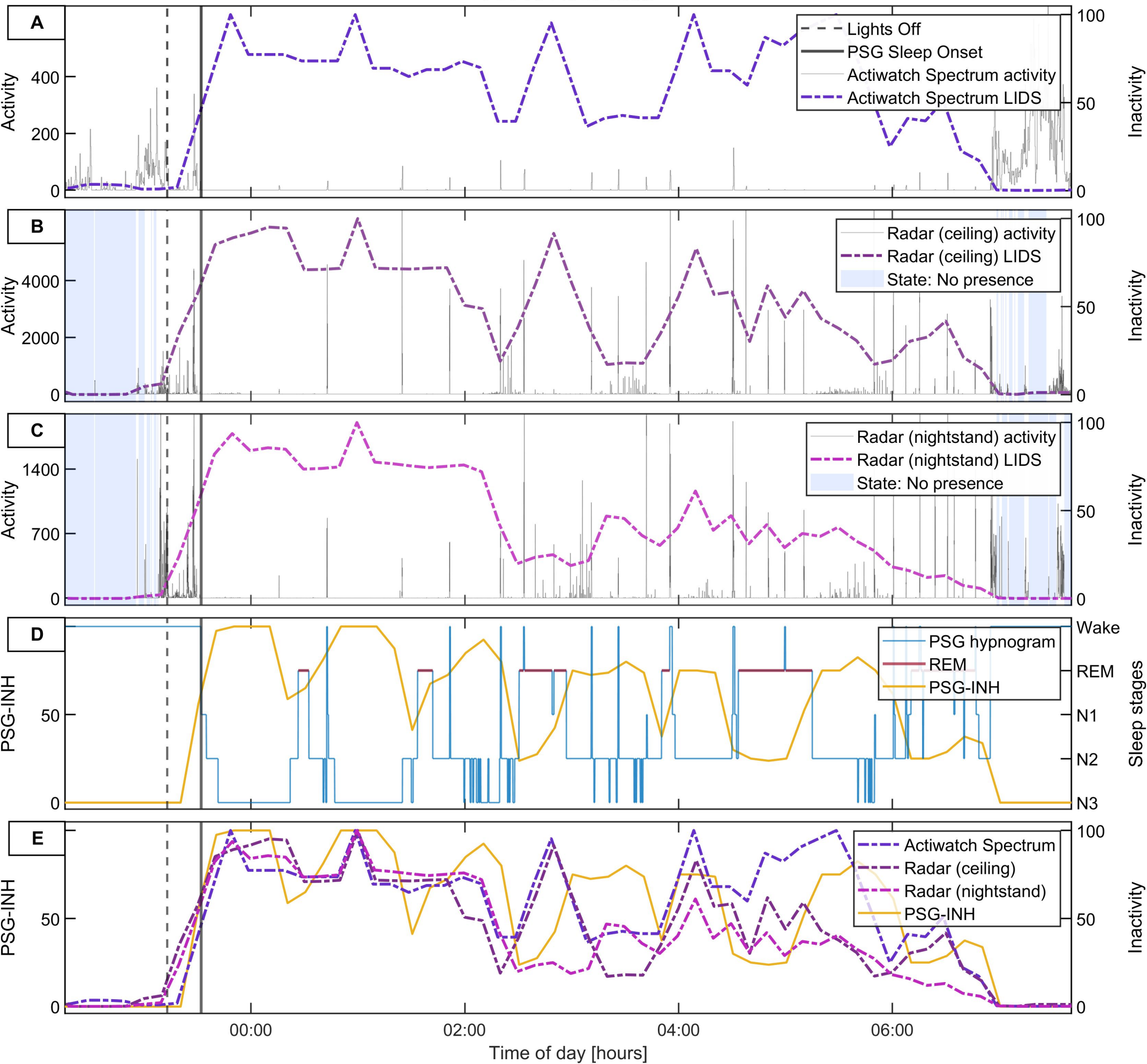

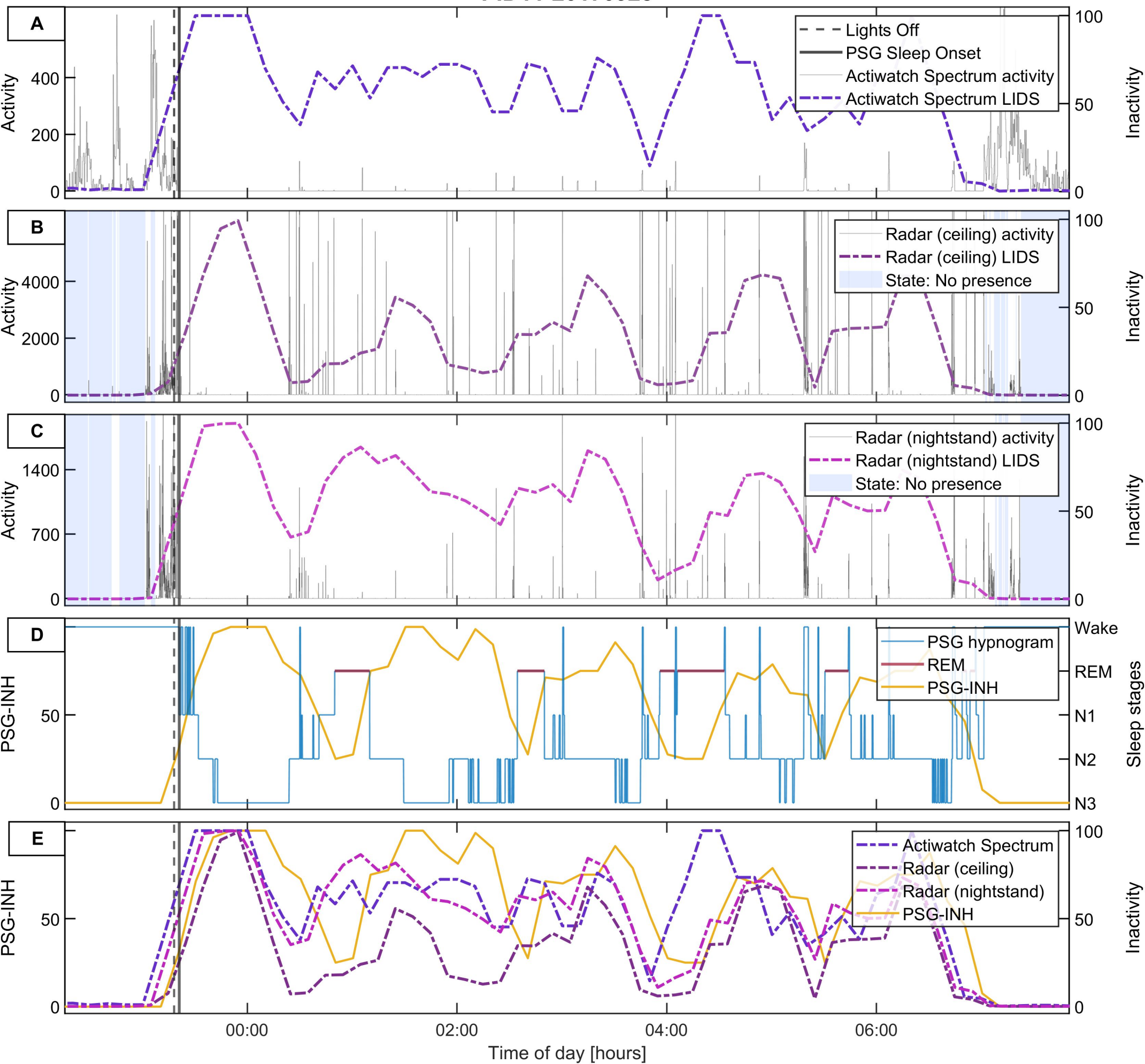

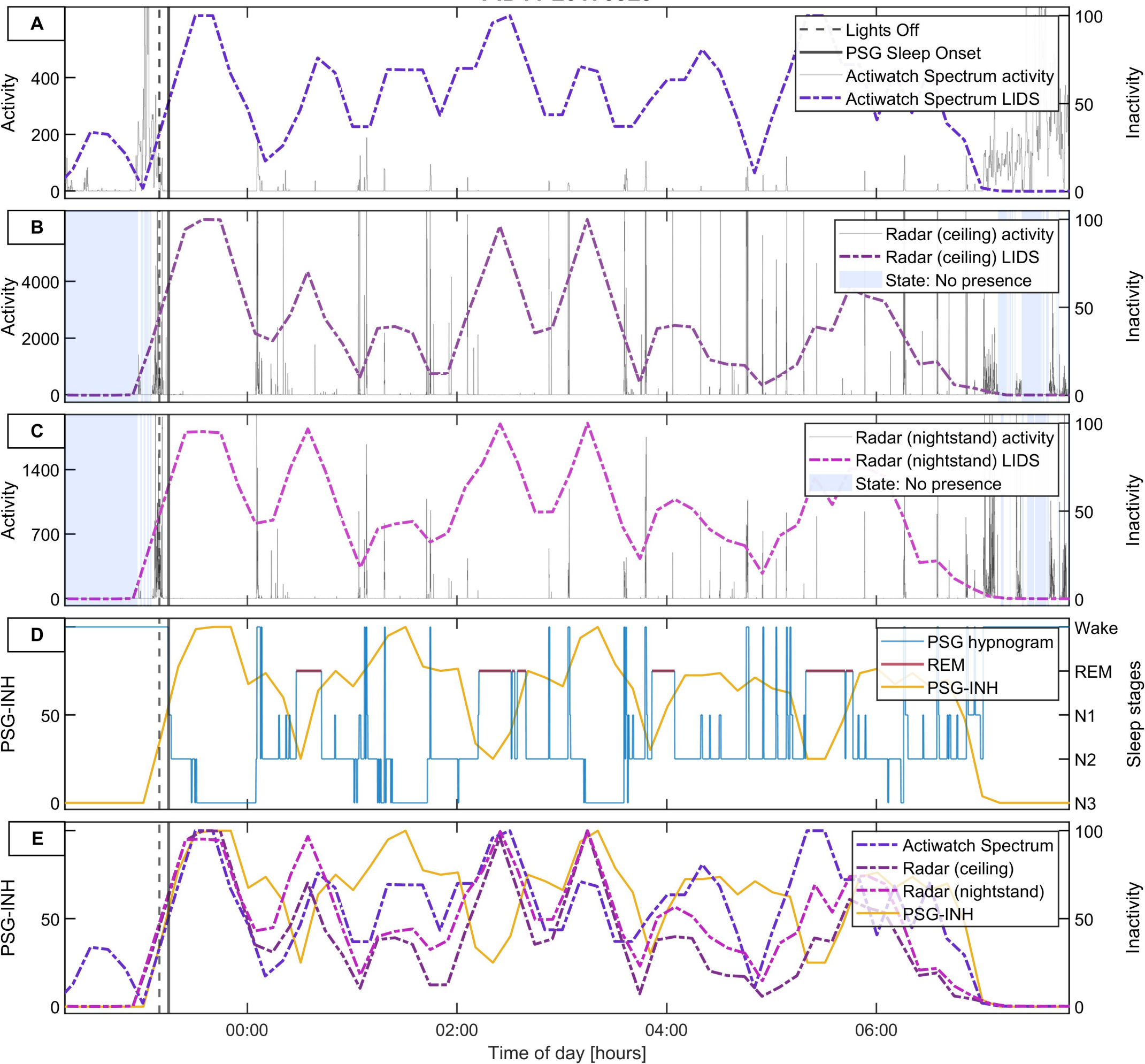

# PID11-20171004

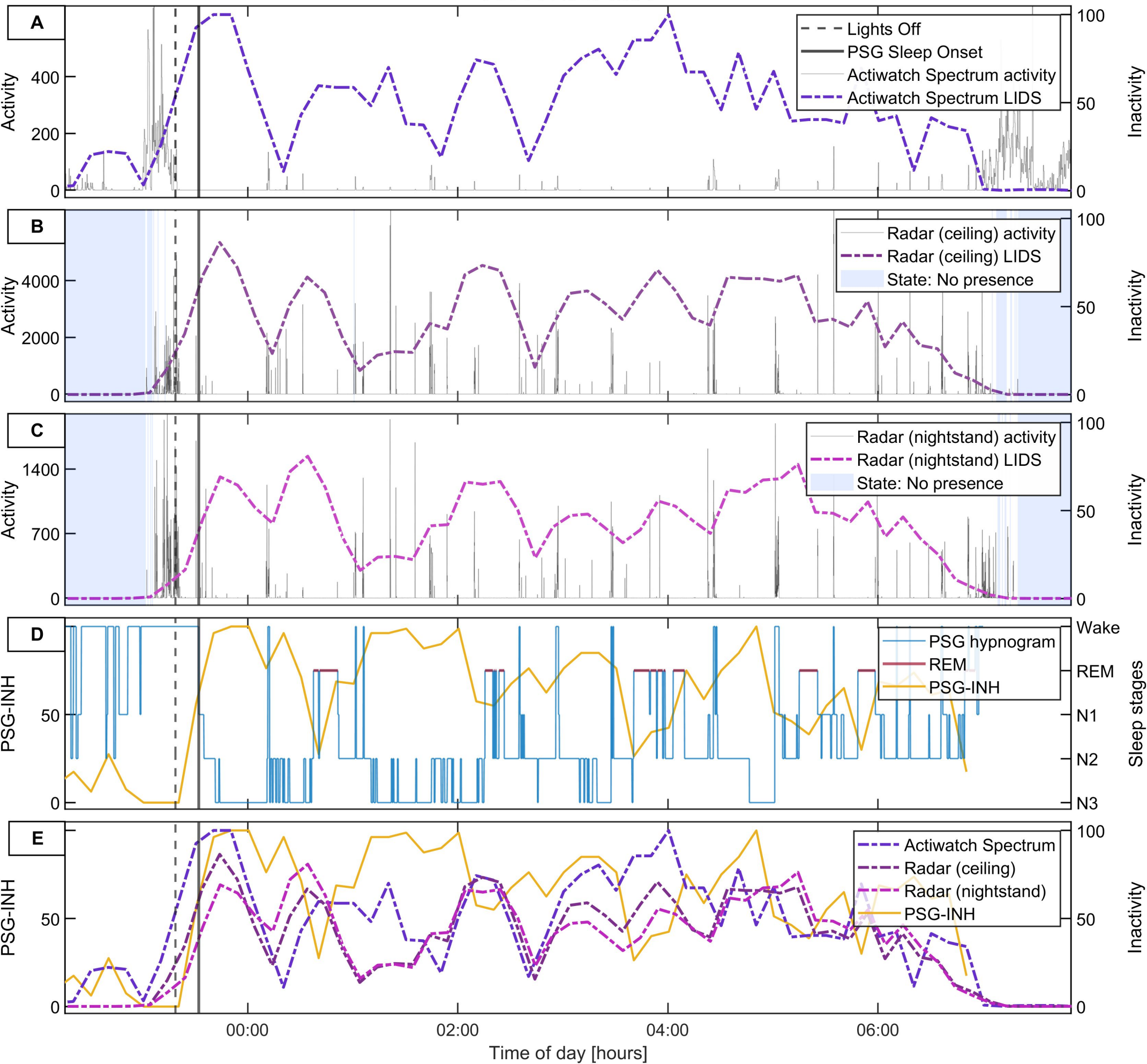

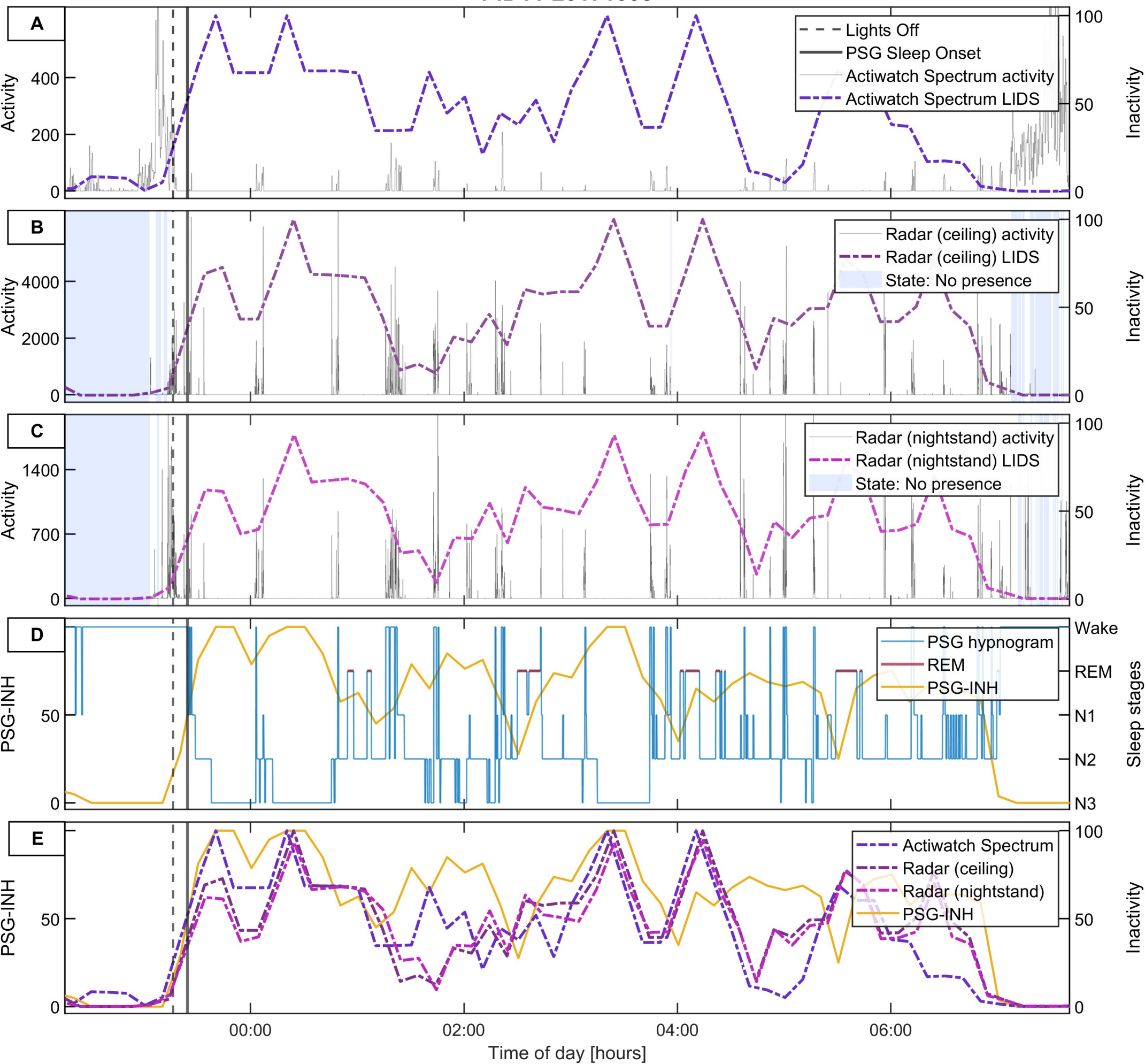

# PID12-20170928

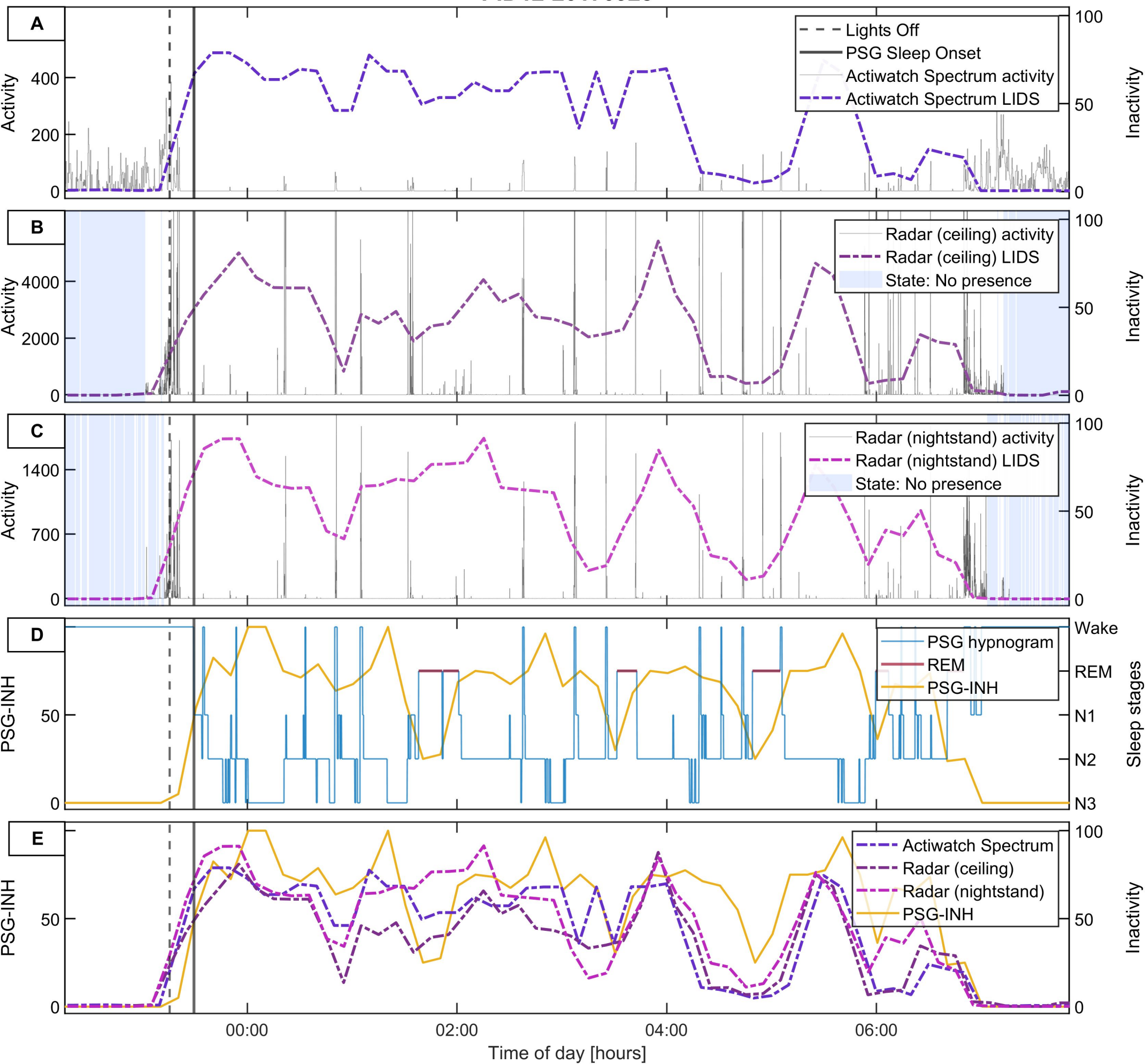

# PID12-20170929

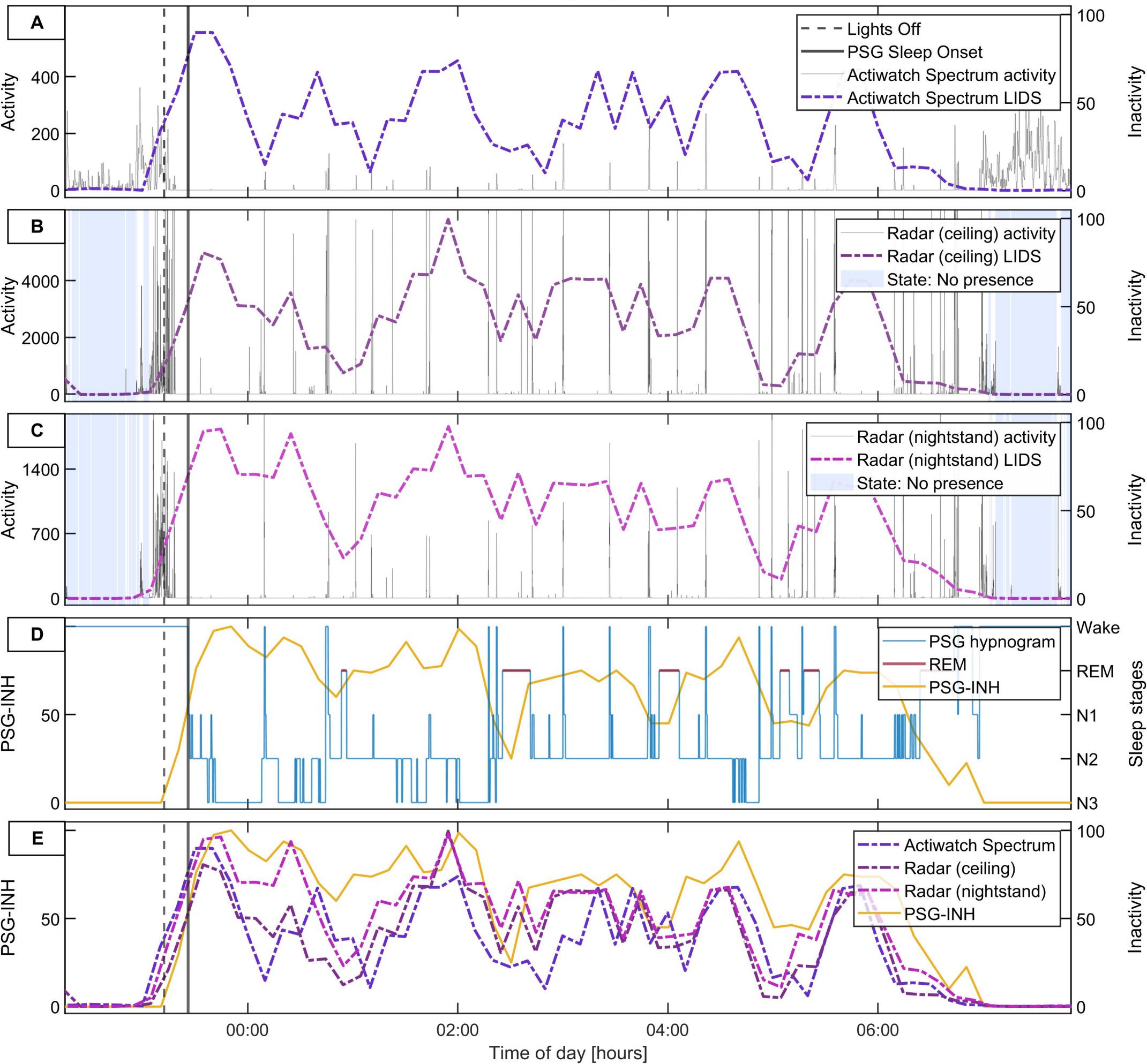

# PID12-20171004

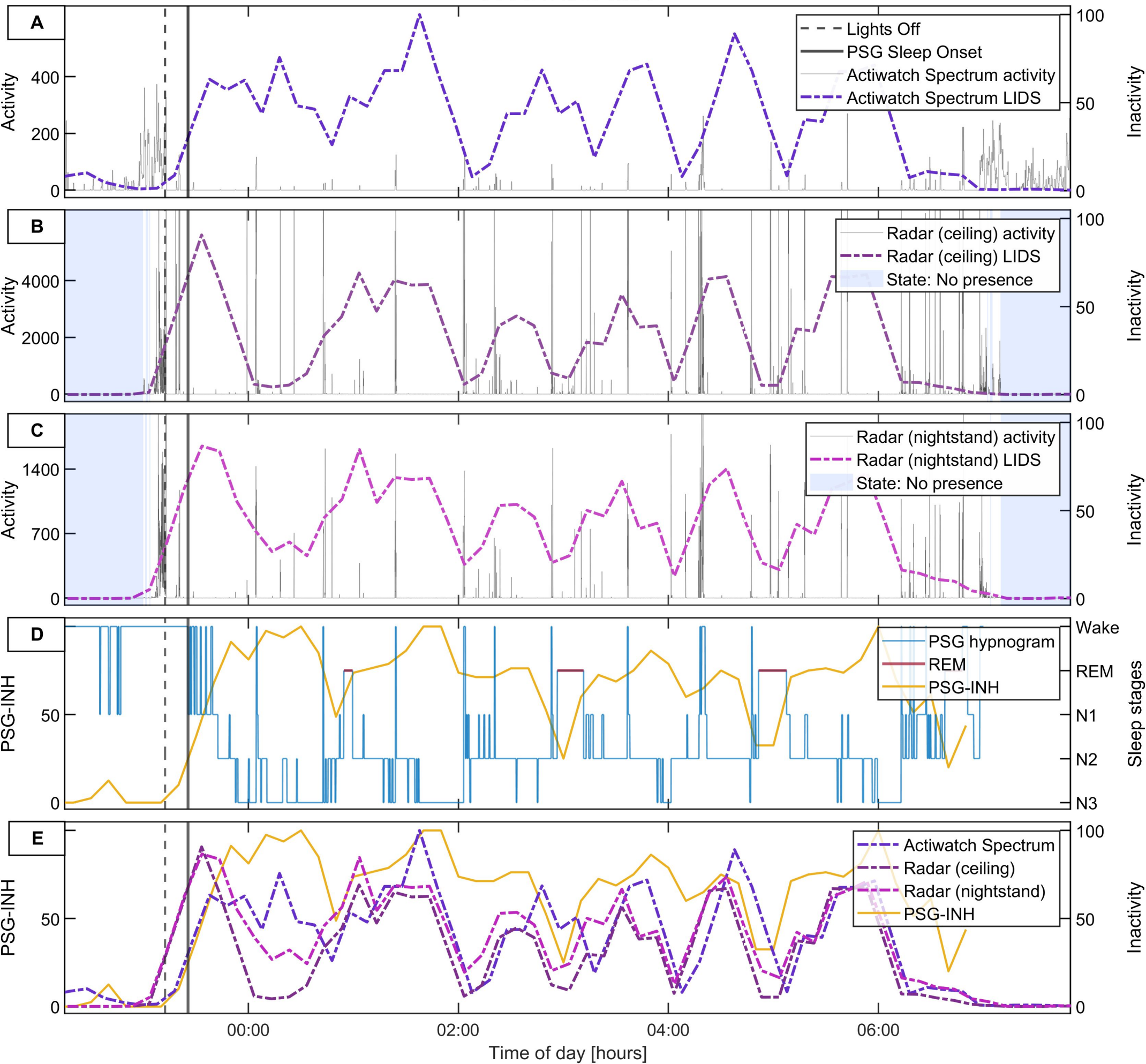

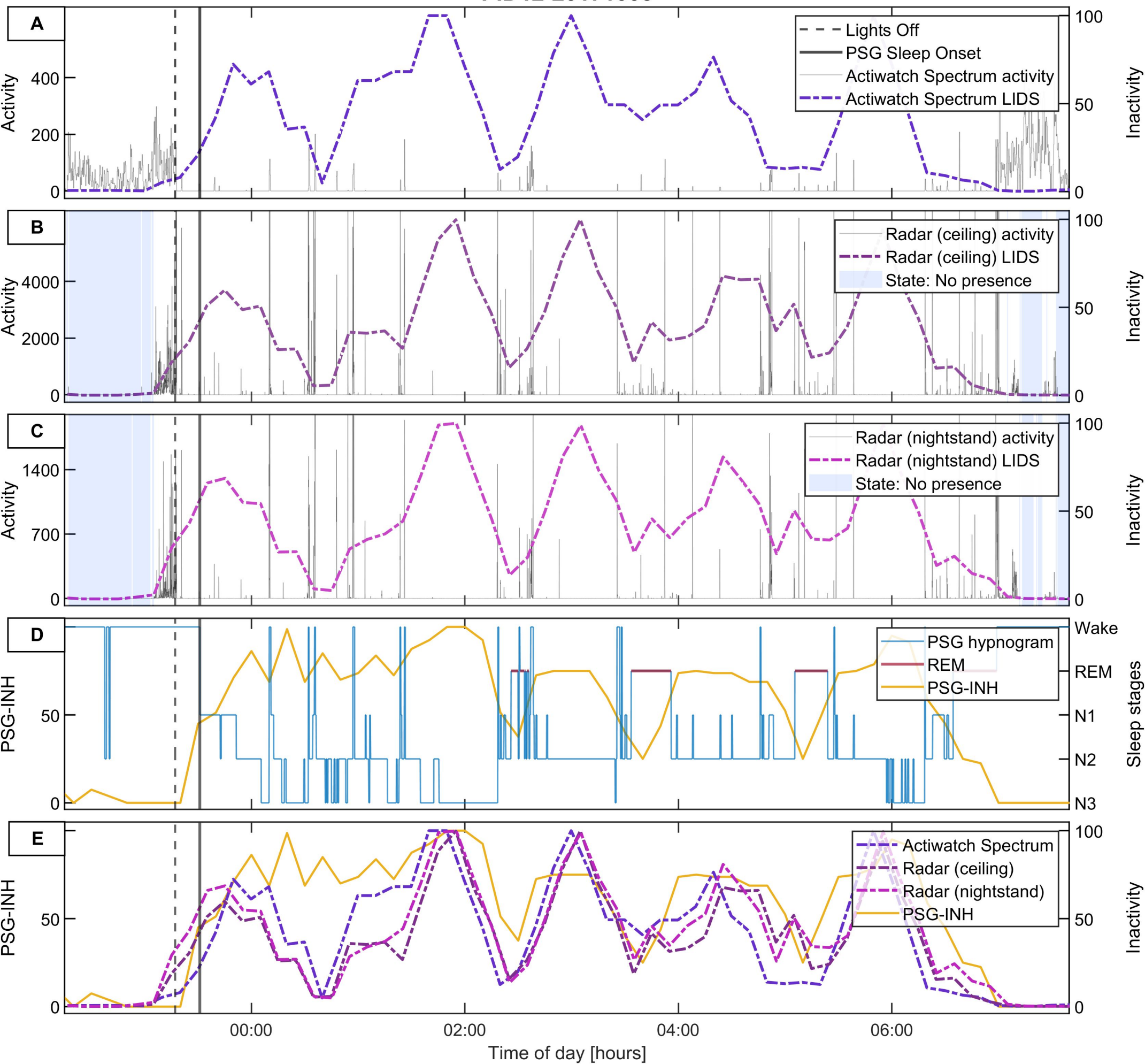

# PID13-20170926

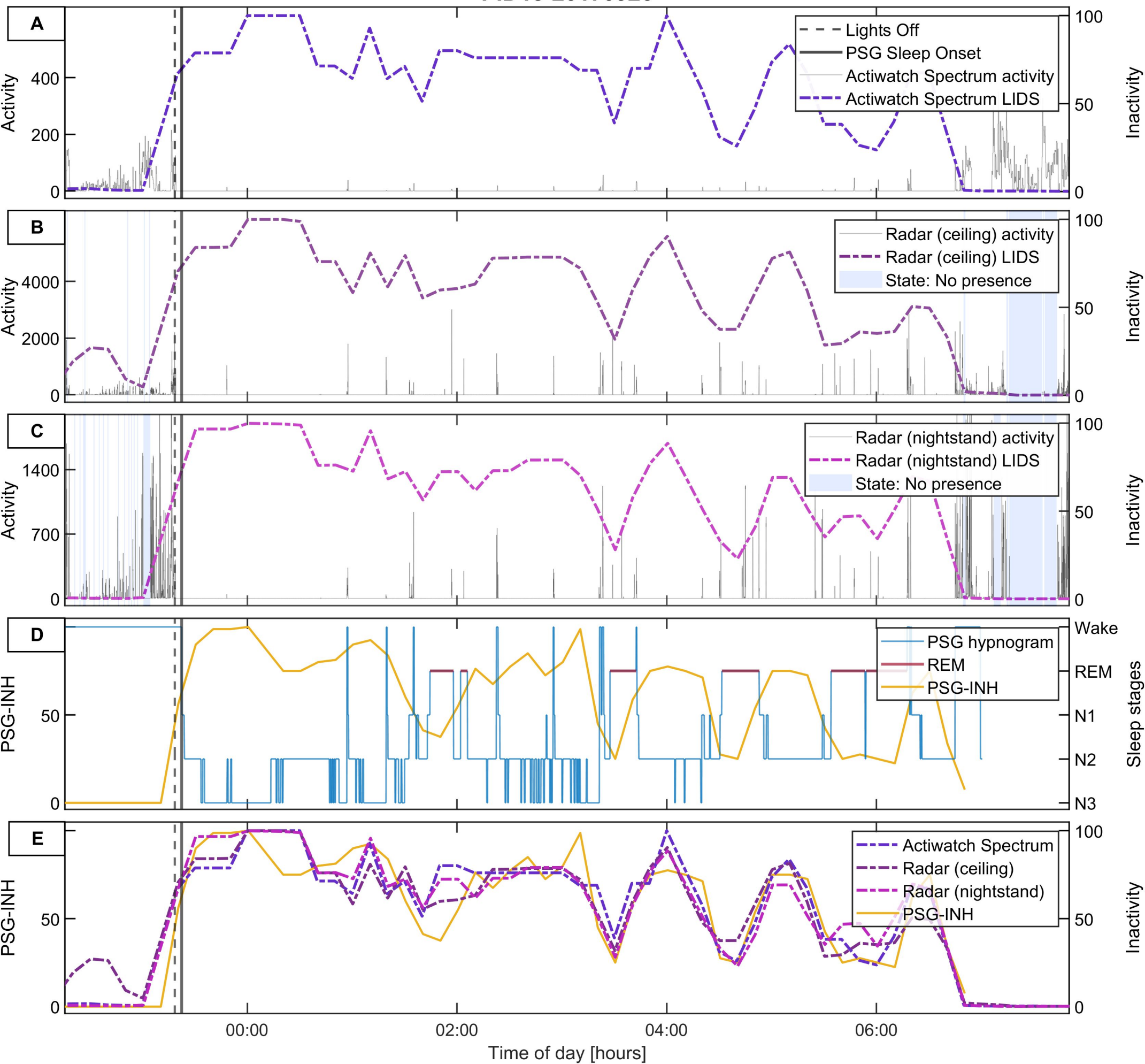

# PID13-20170927

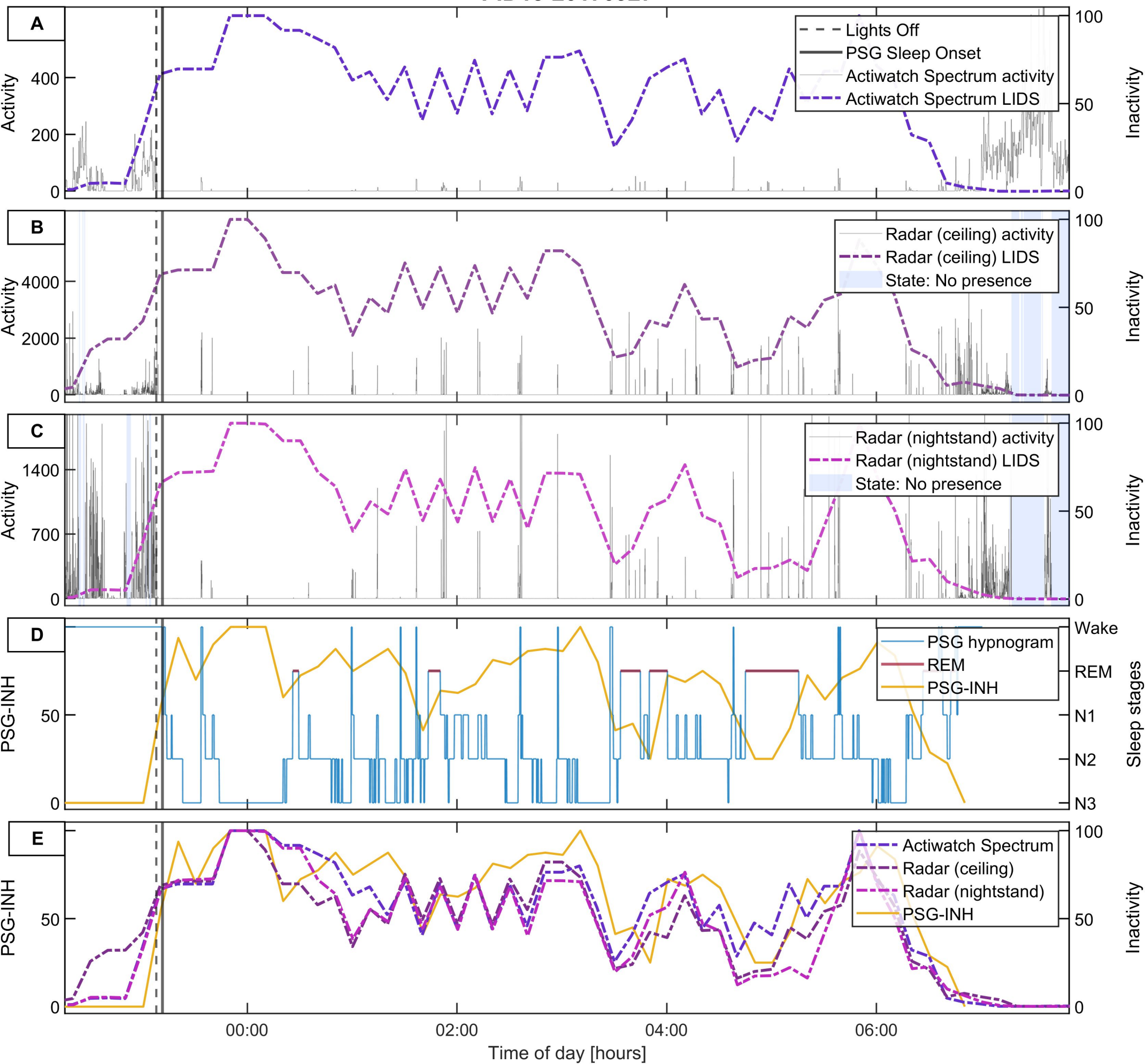

Supplement: Supplementary file 1 — Appendix S1 Supporting Information [file JSR-31-e13687-s001.zip › JSR_13687_Supporting Figures_Figure3.pdf]
